# Supplementary material for: RNA structure prediction using positive and negative evolutionary information
Source: PLoS Comput Biol. 2020 Oct 30;16(10):e1008387. doi: 10.1371/journal.pcbi.1008387 (PMC7657543; doi:10.1371/journal.pcbi.1008387)
Supplement: S1 File — Includes alignments, and source code. (GZ) [file pcbi.1008387.s008.tar.gz › supplemental_material/src/rscape_v1.5.4.tar.gz!/rscape_v1.5.4/lib/infernal/infernal-1.1.2/Userguide.pdf]

INFERNAL User's Guide

Sequence analysis using profiles of RNA sequence and secondary structure consensus

http://eddylab.org/infernal

Version 1.1.2; July 2016

Eric Nawrocki and Sean Eddy

for the INFERNAL development team

github.com/EddyRivasLab/infernal/

Contents

1 Introduction **5**
How to avoid reading this manual 5
What covariance models are 5
Applications of covariance models 6
Infernal and HMMER, CMs and profile HMMs 6
What's new in Infernal 1.1 7
How to learn more about CMs and profile HMMs 7

2 Installation **9**
Quick installation instructions 9
System requirements 9
Multithreaded parallelization for multicores is the default 10
MPI parallelization for clusters is optional 10
Using build directories 11
Makefile targets 11
Why is the output of 'make' so clean? 11
What gets installed by 'make install', and where? 11
Staged installations in a buildroot, for a packaging system 12
Workarounds for some unusual configure/compilation problems 12

3 Tutorial **14**
The programs in Infernal 14
Files used in the tutorial 14
Searching a sequence database with a single covariance model 15
Step 1: build a covariance model with cmbuild 15
Step 2: calibrate the model with cmcalibrate 16
Step 3: search a sequence database with cmsearch 17
Truncated RNA detection 23
Searching a CM database with a query sequence 23
Step 1: create an CM database flatfile 23
Step 2: compress and index the flatfile with cmpress 24
Step 3: search the CM database with cmscan 24
Truncated hit and local end alignment example 26
Searching the Rfam CM database with a query sequence 27
Creating multiple alignments with cmalign 29
cmalign assumes sequences may be truncated 30
Searching a sequence database for RNAs with unknown or no secondary structure 31
Forcing global CM alignment with the -g option 33
Specifying and annotating match positions with cmbuild –hand 33

4 Infernal 1.1's profile/sequence comparison pipeline **36**
Filter thresholds are dependent on database size 37
Manually setting filter thresholds 38
In more detail: profile HMM filter stages 39
Null model. 39
SSV filter. 40
Local Viterbi filter. 40
Biased composition filter. 41
Local Forward filter. 41
Glocal Forward filter. 42

Envelope definition. 43
In more detail: CM stages of the pipeline 44
HMM band definition for CM stages. 44
HMM banded CM CYK filter. 44
HMM banded CM Inside filter/parser. 45
Optimal accuracy alignment. 45
Biased composition CM score correction: the null3 model. 45
Truncated hit detection using variants of the pipeline 48
Differences between the standard pipeline and the truncated variants 48
Modifying how truncated hits are detected using command-line options 49
HMM-only pipeline variant for models without structure 49

5 Profile SCFG construction: the `cmbuild` program 51
Technical description of a covariance model 51
Definition of a stochastic context free grammar 51
SCFG productions allowed in CMs 51
From consensus structural alignment to guide tree 52
From guide tree to covariance model 53
Parameterization 54
Comparison to profile HMMs 54
The `cmbuild` program, step by step 56
Alignment input file 56
Parsing secondary structure annotation 56
Sequence weighting 57
Architecture construction 57
Parameterization 58
Naming the model 58
Saving the model 58

6 Tabular output formats 59
Target hits tables 59
Target hits table format 1 59
Target hits table format 2 60

7 Some other topics 62
How do I cite Infernal? 62
How do I report a bug? 62
Input files 63
Reading from a stdin pipe using - (dash) as a filename argument 63

8 Manual pages 65
`cmalign` - align sequences to a covariance model 65
Synopsis 65
Description 65
Options 66
Options for Controlling the Alignment Algorithm 66
Options for Controlling Speed and Memory Requirements 67
Optional Output Files 68
Other Options 68
`cmbuild` - construct covariance model(s) from structurally annotated 70
Synopsis 70
Description 70

Options 70
Options Controlling Model Construction 71
Other Model Construction Options 71
Options Controlling Relative Weights 71
Options Controlling Effective Sequence Number 72
Options Controlling Filter P7 Hmm Construction 72
Options Controlling Filter P7 Hmm Calibration 73
Options for Refining the Input Alignment 73
`cmcalibrate` - fit exponential tails for covariance model E-value determination 74
Synopsis 74
Description 74
Options 74
Options for Predicting Required Time and Memory 74
Options Controlling Exponential Tail Fits 75
Optional Output Files 75
Other Options 75
`cmconvert` - convert Infernal covariance model files 77
Synopsis 77
Description 77
Options 77
`cmemit` - sample sequences from a covariance model 78
Synopsis 78
Description 78
Options 78
Options for Truncating Emitted Sequences 79
Other Options 79
`cmfetch` - retrieve covariance model(s) from a file 80
Synopsis 80
Description 80
Options 80
`cmpress` - prepare a covariance model database for cmscan 81
Synopsis 81
Description 81
Options 81
`cmscan` - search sequence(s) against a covariance model database 82
Synopsis 82
Description 82
Options 82
Options for Controlling Output 83
Options Controlling Reporting Thresholds 83
Options for Inclusion Thresholds 83
Options for Model-specific Score Thresholding 84
Options Controlling the Acceleration Pipeline 84
Other Options 86
`cmsearch` - search covariance model(s) against a sequence database 88
Synopsis 88
Description 88
Options 88
Options for Controlling Output 89
Options Controlling Reporting Thresholds 89
Options for Inclusion Thresholds 89

Options for Model-specific Score Thresholding . . . 90
Options Controlling the Acceleration Pipeline . . . 90
Other Options . . . 91
`cmstat` - summary statistics for a covariance model file . . . 94
Synopsis . . . 94
Description . . . 94
Options . . . 94

9 File and output formats 96
Infernal CM files . . . 96
CM header section . . . 97
CM main model section . . . 99
HMMER3 filter HMM format . . . 101
HMM header section . . . 101
HMM main model section . . . 103
RNA secondary structures: WUSS notation . . . 104
Full (output) WUSS notation . . . 104
Shorthand (input) WUSS notation . . . 107
Stockholm, the recommended multiple sequence alignment format . . . 107
syntax of Stockholm markup . . . 108
semantics of Stockholm markup . . . 108
recognized #=GF annotations . . . 108
recognized #=GS annotations . . . 109
recognized #=GC annotations . . . 109
recognized #=GR annotations . . . 109
Sequence files: FASTA format . . . 109
Null model file format . . . 110
Clan input file format for cmscan . . . 110
Dirichlet prior files . . . 110

10 Acknowledgements 113

1 Introduction

Infernal is used to search sequence databases for homologs of structural RNA sequences, and to make sequence- and structure-based RNA sequence alignments. Infernal builds a *profile* from a structurally annotated multiple sequence alignment of an RNA family with a position-specific scoring system for substitutions, insertions, and deletions. Positions in the profile that are basepaired in the h consensus secondary structure of the alignment are modeled as dependent on one another, allowing Infernal's scoring system to consider the secondary structure, in addition to the primary sequence, of the family being modeled. Infernal profiles are probabilistic models called "covariance models", a specialized type of stochastic context-free grammar (SCFG) (Lari and Young, 1990).

Compared to other alignment and database search tools based only on sequence comparison, Infernal aims to be significantly more accurate and more able to detect remote homologs because it models sequence and structure. But modeling structure comes at a high computational cost, and the slow speed of CM homology searches has been a serious limitation of previous versions. With version 1.1, typical homology searches are now about 100x faster, thanks to the incorporation of accelerated HMM methods from the HMMER3 software package (`http://hmmer.org`), making Infernal a much more practical tool for RNA sequence analysis.

How to avoid reading this manual

If you're like most people, you don't enjoy reading documentation. You're probably thinking: 113 pages of documentation, you must be joking! I just want to know that the software compiles, runs, and gives apparently useful results, before I read some 113 exhausting pages of someone's documentation. For cynics that have seen one too many software packages that don't work:

- Follow the quick installation instructions on page 9. An automated test suite is included, so you will know immediately if something went wrong.[1]
- Go to the tutorial section on page 14, which walks you through some examples of using Infernal on real data.

Everything else, you can come back and read later.

What covariance models are

Covariance models (CMs) are statistical models of structurally annotated RNA multiple sequence alignments, or even of single sequences and structures. CMs are a specific formulation of profile stochastic context-free grammars (profile SCFG), which were introduced independently by Yasu Sakakibara in David Haussler's group (Sakakibara et al., 1994) and by Sean Eddy and Richard Durbin (Eddy and Durbin, 1994). CMs are closely related to profile hidden Markov models (profile HMMs) commonly used for protein sequence analysis, but are more complex. CMs and profile HMMs both capture position-specific information about how conserved each column of the alignment is, and which residues are likely. However, in a profile HMM each position of the profile is treated independently, while in a CM basepaired positions are dependent on one another. The dependency between paired positions in a CM enables the profile to model *covariation* at these positions, which often occurs between basepaired columns of structural RNA alignments. For many of these basepairs, it is not the specific nucleotides that make up the pair that is conserved by evolution, but rather that the pair maintain Watson-Crick basepairing. The added signal from covariation can be significant when using CMs for homology searches in large databases. Section 5 of this guide explains how a CM is constructed from a structurally annotated alignment using a toy example.

CMs do have important limitations though. For example, a CM can only model what is called a "well-nested" set of basepairs. Formally, in a well-nested set of basepairs there are no two basepairs between

[1] Nothing should go wrong.

positions $i : j$ and $k : l$ such that $i < k < j < l$. CMs cannot model pseudoknots in RNA secondary structures. Additionally, a CM only models a single consensus structure for the family it models.

Applications of covariance models

Infernal can be useful if you're intereseted in a particular RNA family. Imagine that you've carefully collected and aligned a set of homologs and have a predicted (or known) secondary structure for the family. Homology searches with BLAST using single sequences from your set of homologs may not reveal any additional homologs in sequence databases. You can build a CM from your alignment and redo your search using Infernal (this time only a single search) and you may find new homologs thanks to the added power of the profile-based sequence and structure scoring system of CMs. The Rfam database (Gardner et al., 2011) essentially does just this, but on a much larger scale. The Rfam curators maintain about 2000 RNA families, each represented by a multiple sequence alignment (called a *seed* alignment) and a CM built from that alignment. Each Rfam release involves a search through a large EMBL-based nucleotide sequence database with each of the CMs which identifies putative structural RNAs in the database. The annotations of these RNAs, as well as the CMs and seed alignments are freely available.

Automated genome annotation of structural RNAs can be performed with Infernal and a collection of CMs from Rfam, by searching through the genome of interest with each CM and collecting information on high-scoring hits. Previous versions of Infernal were too slow to be incorporated into many genome annotation pipelines, but we're hoping the improved speed of version 1.1 changes this.

Another application is the automated construction and maintenance of large sequence- and structure-based multiple alignment databases. For example, the Ribosomal Database Project uses CMs of 16S small subunit ribosomal RNA (16S SSU rRNA) to maintain alignments of millions of 16S sequences (Cole et al., 2009). The CMs (one archaeal 16S and one bacterial 16S model) were built from training alignments of only a few hundred representative sequences. The manageable size of the training alignments means that they can be manually curated prior to building the model. Rfam is another example of this application too because Rfam creates and makes available multiple alignments (called *full* alignments) of all of the hits from the database its curators believe to be real RNA homologs.

Infernal can also be used to determine what types of RNAs exist in a particular sequence dataset. Suppose you're performing a metagenomics analysis and have collected sequences from an exotic environmental sample. You can download all the CMs from Rfam and use Infernal to search through all your sequences for high-scoring hits to the models. The types of structural RNAs identified in your sample can be informative as to what types of organisms are in your sample, and what types of biological processes they're carrying out. Version 1.1 includes a new program called `cmscan` which is designed for just this type of analysis.

Infernal and HMMER, CMs and profile HMMs

Infernal is closely related to HMMER. In fact, HMMER is used as a library within the Infernal codebase. This allows Infernal to use the highly optimized profile HMM dynamic programming implementations in HMMER to greatly accelerate its homology searches. Also, the design and organization of the Infernal programs (e.g. `cmbuild`, `cmsearch`, `cmalign`) follows that in HMMER (`hmmbuild`, `hmmsearch`, `hmmalign`). And there are many functions in Infernal that are based on analogous ones in HMMER. The formatting of output is often very similar between these two software packages, and the user guide's are even organized and written in a similar (and, in some places, identical) way.

This is, of course, on purpose. Since both packages are developed in the same lab, consistency simplifies the development and maintenance of the code, but we also do it to make the software (hopefully) easier to use (someone familiar with using HMMER should be able to pick up and use Infernal very easily, and vice versa). However, Infernal development tends to lag behind HMMER development as new ideas and algorithms are applied to the protein or DNA world with profile HMMs, and then later extended to CMs for use on RNAs.

This consistency is possible because profile HMMs and covariance models are related models with related applications. Profile HMMs are profiles of the conserved sequence of a protein or DNA family and CMs are profiles of the conserved sequence *and* well-nested secondary structure of a structural RNA family. Applications of profile HMMs include annotating protein sequences in proteomes or protein sequence database and creating multiple alignments of protein domain families. And similarly applications of CMs include annotating structural RNAs in genomes or nucleotide sequence databases and creating sequence- and structure-based multiple alignments of RNA. The crucial difference is that CMs are able to model dependencies between a set of well-nested (non-pseudoknotted) basepaired positions in a structural RNA family. The statistical signal inherent in these dependencies is often significant enough to make modeling the family with a CM a noticeably more powerful approach than modeling the family with a profile HMM.

What's new in Infernal 1.1

The most important difference between version 1.1 and the previous version (1.0.2) is the improved search speed that results from a new filter pipeline. The pipeline is explained more in section 4. Another important change is the introduction of the `cmscan` program, for users who want to know what structural RNAs are present in a collection of sequences, such as a metagenomics dataset[2]. Another new feature of version 1.1 is better handling of truncated RNAs, for which part of one or both ends of the RNA is missing due to a premature end of the sequence (Kolbe and Eddy, 2009). These types of fragmentary sequences are common in whole genome shotgun sequencing datasets. While previous versions of Infernal were prone to misalignment of these sequences, version 1.1 includes implementations of CM search and alignment algorithms specialized for truncated sequences (Kolbe and Eddy, 2009) in `cmsearch`, `cmscan` and `cmalign`.

Model parameterization has changed in several minor ways. Mixture Dirichlet priors for emissions and single component Dirichlet priors for transitions have been reestimated using larger and more diverse datasets than the ones the previous priors were derived from (discussed in (Nawrocki and Eddy, 2007)). Also, the definition of match and insert columns, previously determined by a simple majority rule using absolute counts (columns in which $\geq 50\%$ of columns include residues were match, all others were insert), now use *weighted* counts (and same $>= 50\%$ rule) after a sequence weighting algorithm is applied. And inserts before the first and after the final match position of alignments are now ignored by the CM construction procedure and thus no longer contribute to parameterizing the transition probabilities of the model (specifically, the `ROOT_IL` and `ROOT_IR` states). These changes mean that for a given input alignment a model built with version 1.1 may have different numbers of states and nodes, and will have (usually) slightly different parameters, than a model built from the same alignment with version 1.0.2. Finally, the important `cmbuild` command line options `--rf` and `--gapthresh` have been renamed to `--hand` and `--symfrac`[3].

The formatting of `cmsearch` output has also changed. It mirrors the output format of the `hmmsearch` program from HMMER3, for examples see the tutorial section of this guide. Another change is that the most compute-intensive programs in Infernal 1.1 (`cmcalibrate`, `cmsearch`, `cmscan` and `cmalign`) support multicore parallelization using threads.

How to learn more about CMs and profile HMMs

Section 5 of this guide may be a good place to start. That section walks through an example of how a CM is constructed from a structurally annotated multiple sequence alignment. The tutorial section is also recommended for all users.

As for other available publications: two papers published in 1994 introduced profile SCFGs in computational biology (Sakakibara et al., 1994; Eddy and Durbin, 1994), and our lab has published several papers (Eddy, 2002; Klein and Eddy, 2003; Nawrocki and Eddy, 2007; Nawrocki et al., 2009; Kolbe and Eddy, 2009, 2011), book chapters (Eddy, 2006; **?**), and a few doctoral theses (Klein, 2003; Nawrocki, 2009;

[2] `cmscan` is similar to `cmsearch` but is more convenient for some applications. One difference between the two programs is that results from `cmscan` are organized per-sequence instead of per-model.

[3] To reproduce the behavior obtained in previous versions with `--gapthresh <x>` use `--symfrac <1-x>`.

Kolbe, 2010) related to CMs[4]. The book *Biological Sequence Analysis: Probabilistic Models of Proteins and Nucleic Acids* (Durbin et al., 1998) has several chapters devoted to HMMs and CMs. Profile HMM filtering for CMs was introduced by Weinberg and Ruzzo (Weinberg and Ruzzo, 2004a,b, 2006). There are two papers from our lab on HMMER3 profile HMMs that are directly related to Infernal's accelerated filter pipeline (Eddy, 2008, 2011).

Since CMs are closely related to, but more complex than, profile HMMs, readers seeking to understand CMs who are unfamiliar with profile HMMs may want to start there. Reviews of the profile HMM literature have been written by our lab (Eddy, 1996, 1998) and by Anders Krogh (Krogh, 1998). And to learn more about HMMs from the perspective of the speech recognition community, an excellent tutorial introduction has been written by Rabiner (Rabiner, 1989). For details on how profile HMMs and probabilistic models are used in computational biology, see the pioneering 1994 paper from Krogh et al. (Krogh et al., 1994) and again the *Biological Sequence Analysis* book (Durbin et al., 1998).

Finally, Sean Eddy writes about HMMER, Infernal and other lab projects in his blog **Cryptogenomicon** `http://cryptogenomicon.org/`).

> ▷ ***How do I cite Infernal?*** *The Infernal 1.1 paper (Infernal 1.1: 100-fold faster RNA homology searches, EP Nawrocki and SR Eddy. Bioinformatics, 29:2933-2935, 2013.) is the most appropriate paper to cite. If youre writing for an enlightened (url-friendly) journal, you may want to cite the webpage* `http://eddylab.org/infernal/` *because it is kept up-to-date.*

[4]Eddy lab publications are available from `http://eddylab.org/publications.html`

2 Installation

Quick installation instructions

Download `infernal-1.1.2.tar.gz` from `http://eddylab.org/infernal/`, or directly from `eddylab.org/infernal/infernal-1.1.2.tar.gz`; unpack it, configure, and make:

```
> wget eddylab.org/infernal/infernal-1.1.2.tar.gz
> tar xf infernal-1.1.2.tar.gz
> cd infernal-1.1.2
> ./configure
> make
```

To compile and run a test suite to make sure all is well, you can optionally do:

```
> make check
```

All these tests should pass.

You don't have to install Infernal programs to run them. The newly compiled binaries are now in the `src` directory. You can run them from there. To install the programs and man pages somewhere on your system, do:

```
> make install
```

By default, programs are installed in `/usr/local/bin` and man pages in `/usr/local/share/man/man1/`. You can change the `/usr/local` prefix to any directory you want using the `./configure --prefix` option, as in `./configure --prefix /the/directory/you/want`.

Optionally, you can install the Easel library package as well, including its various "miniapplications", in addition to its library and header files. We don't do this by default, in case you already have a copy of Easel separately installed:

```
> cd easel; make install
```

That's it. You can keep reading if you want to know more about customizing a Infernal installation, or you can skip ahead to the next chapter, the tutorial.

System requirements

Operating system: Infernal is designed to run on POSIX-compatible platforms, including UNIX, Linux and MacOS/X. The POSIX standard essentially includes all operating systems except Microsoft Windows. We have tested most extensively on Linux and on MacOS/X, because these are the machines we develop on.

Processor: Infernal depends on vector parallelization methods that are supported on most modern processors. Infernal requires either an x86-compatible (IA32, IA64, or Intel64) processor that supports the SSE2 vector instruction set, or a PowerPC processor that supports the Altivec/VMX instruction set. SSE2 is supported on Intel processors from Pentium 4 on, and AMD processors from K8 (Athlon 64) on; we believe this includes almost all Intel processors since 2000 and AMD processors since 2003. Altivec/VMX is supported on Motorola G4, IBM G5, and IBM PowerPC processors starting with the Power6, which we believe includes almost all PowerPC-based desktop systems since 1999 and servers since 2007.

If your platform does not support one of these vector instruction sets, you won't be able to install and run Infernal 1.1 on it.

We do aim to be portable to all modern processors. The acceleration algorithms are designed to be portable despite their use of specialized SIMD vector instructions. We hope to add support for the Sun SPARC VIS instruction set, for example. We believe that the code will be able to take advantage of GP-GPUs and FPGAs in the future.

Compiler: The source code is C conforming to POSIX and ANSI C99 standards. It should compile with any ANSI C99 compliant compiler, including the GNU C compiler `gcc`. We test the code using both the `gcc` and `icc` compilers.

Libraries and other installation requirements: Infernal includes two software libraries, HMMER and Easel, which it will automatically compile during its installation process. By default, Infernal does not require any additional libraries to be installed by you, other than standard ANSI C99 libraries that should already be present on a system that can compile C code. Bundling HMMER and Easel instead of making them separate installation requirements is a deliberate design decision to simplify the installation process.[1]

Configuration and compilation use several UNIX utilities. Although these utilities are available on all UNIX/Linux/MacOS systems, old versions may not support all the features the `./configure` script and Makefiles are hoping to find. We aim to build on anything, even old Ebay'ed junk, but if you have an old system, you may want to hedge your bets and install up-to-date versions of GNU tools such as GNU make and GNU grep.

Multithreaded parallelization for multicores is the default

The main workhorse Infernal programs `cmalign`, `cmcalibrate`, `cmsearch` and `cmscan` support multicore parallelization using POSIX threads. By default, the configure script will identify whether your platform supports POSIX threads (almost all platforms do), and will automatically compile in multithreading support.

If you want to disable multithreading at compile time, recompile from source after giving the `--disable-threads` flag to `./configure`.

By default, our multithreaded programs will use all available cores on your machine. You can control the number of cores each Infernal process will use for computation with the `--cpu <x>` command line option or the `INFERNAL_NCPU` environment variable. Even with a single processing thread (`--cpu 1`), INFERNAL will devote a second execution thread to database input, resulting in speedup over serial execution.

If you specify `--cpu 0`, the program will run in serial-only mode, with no threads. This might be useful if you suspect something is awry with the threaded parallel implementation.

MPI parallelization for clusters is optional

The `cmalign`, `cmcalibrate`, `cmsearch` and `cmscan` programs also support MPI (Message Passing Interface) parallelization on clusters. To use MPI, you first need to have an MPI library installed, such as OpenMPI (`www.open-mpi.org`).

MPI support is not enabled by default, and it is not compiled into the precompiled binaries that we supply with Infernal. To enable MPI support at compile time, give the `--enable-mpi` option to the `./configure` command.

To use MPI parallelization, each program that has an MPI-parallel mode has an `--mpi` command line option. This option activates a master/worker parallelization mode. (Without the `--mpi` option, if you run a program under `mpirun` on N nodes, you'll be running N independent duplicate commands, not a single MPI-enabled command. Don't do that.)

The MPI implementation for `cmcalibrate` scales well up to 161 processors.[2] `cmalign` scales pretty well up to a couple hundred processors. `cmsearch` scales all right, but the scaling performance will vary on

[1] If you install standalone HMMER (which also bundles Easel), this may become an annoyance; you'll have multiple instantiations of HMMER and Easel lying around. Unfortunately this is necessary as Infernal requires the specific versions of HMMER and Easel bundled within it. Also, the Easel API is not yet stable enough to decouple it from the applications that use it.

[2] By default, `cmcalibrate` searches 160 random sequences of length 10 Kb (1.6 total Mb), so there's no reason to use more than 160 workers plus 1 master - unless you use the `-L <x>` option to increase the total Mb searched (see the `cmcalibrate` man page for more information).

different inputs[3] cmscan scales poorly, and probably shouldn't be used on more than tens of processors at most. Improving MPI scaling is one of our goals.

Using build directories

The configuration and compilation process from source supports using separate build directories, using the GNU-standard VPATH mechanism. This allows you to maintain separate builds for different processors or with different configuration/compilation options. All you have to do is run the configure script from the directory you want to be the root of your build directory. For example:

```
> mkdir my-infernal-build
> cd my-infernal-build
> /path/to/infernal/configure
> make
```

This assumes you have a make that supports VPATH. If your system's make does not, you can always install GNU make.

Makefile targets

all Builds everything. Same as just saying make.

check Runs automated test suites in Infernal, and the HMMER and Easel libraries.

clean Removes all files generated by compilation (by make). Configuration (files generated by ./configure) is preserved.

distclean Removes all files generated by configuration (by ./configure) and by compilation (by make).

Note that if you want to make a new configuration (for example, to try an MPI version by ./configure --enable-mpi; make) you should do a make distclean (rather than a make clean), to be sure old configuration files aren't used accidentally.

Why is the output of 'make' so clean?

Because we're hiding what's really going on with the compilation with a pretty wrapper. If you want to see what the command lines really look like, in all their ugly glory, pass a V=1 option (V for "verbose") to make, as in:

```
> make V=1
```

What gets installed by 'make install', and where?

Infernal's 'make install' generally follows the GNU Coding Standards and the Filesystem Hierarchy Standard. The top-level Makefile has variables that specify three directories where make install will install things:

Variable	What
bindir	All Infernal programs
man1dir	All Infernal man pages
pdfdir	Userguide.pdf

These variables are constructed from some other variables, in accordance with the GNU Coding Standards. All of these variables are at the top of the top-level Makefile. Their defaults are as follows:

[3]A database in which many high-scoring hits are clustered in sequences or exist in many sequences with similar names (that sort close together alphabetically) may show especially poor scaling performance.

Variable	Default
prefix	/usr/local
exec_prefix	$prefix
bindir	$exec_prefix/bin
libdir	$exec_prefix/lib
includedir	$prefix/include
datarootdir	$prefix/share
mandir	$datarootdir/man
man1dir	$mandir/man1

The best way to change these defaults is when you use `./configure`, and the most important variable to consider changing is `--prefix`. For example, if you want to install Infernal in a directory hierarchy all of its own, you might want to do something like:

```
> ./configure --prefix /usr/local/infernal
```

That would keep Infernal out of your system-wide directories like `/usr/local/bin`, which might be desirable. Of course, if you do it that way, you'd also want to add `/usr/local/infernal/bin` to your `$PATH`, `/usr/local/infernal/share/man` to your `$MANPATH`, etc.

These variables only affect `make install`. Infernal executables have no pathnames compiled into them.

Staged installations in a buildroot, for a packaging system

Infernal's `make install` supports staged installations, accepting the traditional `DESTDIR` variable that packagers use to specify a buildroot. For example, you can do:

```
> make DESTDIR=/rpm/tmp/buildroot install
```

Workarounds for some unusual configure/compilation problems

Configuration or compilation fails when trying to use a separate build directory. If you try to build in a build tree (other than the source tree) and you have any trouble in configuration or compilation, try just building in the source tree instead. Some `make` versions don't support the VPATH mechanism needed to use separate build trees. Another workaround is to install GNU make.

Configuration fails, complaining that the CFLAGS don't work. Our configure script uses an Autoconf macro, `AX_CC_MAXOPT`, that tries to guess good optimization flags for your compiler. In very rare cases, we've seen it guess wrong. You can always set `CFLAGS` yourself with something like:

```
> ./configure CFLAGS=-O
```

Configuration fails, complaining "no acceptable grep could be found". We've seen this happen on our Sun Sparc/Solaris machine. It's a known issue in GNU autoconf. You can either install GNU grep, or you can insist to `./configure` that the Solaris grep (or whatever grep you have) is ok by explicitly setting `GREP`:

```
> ./configure GREP=/usr/xpg4/bin/grep
```

Configuration fails with an error message saying that no SSE or VMX capability exists. This is what you get if your system has a processor that we don't yet support the fast vector-parallel implementation of HMM filters that Infernal uses. We currently only support Intel/AMD compatible processors and PowerPC compatible processors. You'll have to install an older version of (version 1.0.2) if you want to use Infernal on other processors.

Many 'make check' tests fail. We have one report of a system that failed to link multithread-capable system C libraries correctly, and instead linked to one or more serial-only libraries.[4] We've been unable to reproduce the problem here, and are not sure what could cause it – we optimistically believe it's a messed-up system instead of our fault. If it does happen, it screws all kinds of things up with the multithreaded implementation. A workaround is to shut threading off:

```
> ./configure --disable-threads
```

This will compile code that won't parallelize across multiple cores, of course, but it will still work fine on a single processor at a time (and MPI, if you build with MPI enabled).

[4]The telltale phenotype of this failure is to configure with debugging flags on and recompile, run one of the failed unit test drivers (such as `easel/easel_utest`) yourself and let it dump core; and use a debugger to examine the stack trace in the core. If it's failed in `__errno_location()`, it's linked a non-thread-capable system C library.

3 Tutorial

Here's a tutorial walk-through of some small projects with Infernal. This should suffice to get you started on work of your own, and you can (at least temporarily) skip the rest of the Guide, such as all the nitty-gritty details of available command line options.

The programs in Infernal

Core programs

**cmbuild**	Build a covariance model from an input multiple alignment.
**cmcalibrate**	Calibrate E-value parameters for a covariance model.
**cmsearch**	Search a covariance model against a sequence database.
**cmscan**	Search a sequence against a covariance model database.
**cmalign**	Make a multiple alignment of many sequences to a common covariance model.

Other utilities

**cmconvert**	Convert CM formats to/from Infernal v1.1 format.
**cmemit**	Generate (sample) sequences from a covariance model.
**cmfetch**	Get a covariance model by name or accession from a CM database.
**cmpress**	Format a CM database into a binary format for `cmscan`.
**cmstat**	Show summary statistics for each model in a CM database.

In this section, we'll show examples of running each of these programs, using examples in the `tutorial/` subdirectory of the distribution.

Files used in the tutorial

The subdirectory `/tutorial` in the Infernal distribution contains the files used in the tutorial, as well as a number of examples of various file formats that Infernal reads. The important files for the tutorial are:

`tRNA5.sto` A multiple alignment of five tRNA sequences. This file is a simple example of *Stockholm format* that Infernal uses for structurally-annotated alignments.

`tRNA5.c.cm` An example CM file. Built with `cmbuild` from `tRNA5.sto` and calibrated using `cmcalibrate`. Included so you don't need to calibrate your own model file, which takes about 20 minutes.

`mrum-genome.fa` The 3 Mb genome of the methanogenic archeaon *Methanobrevibacter ruminantium*, in FASTA format, downloaded from the NCBI Nucleotide database (accession: NC_13790.1).

`tRNA5-mrum.out` An example `cmsearch` output file, obtained by searching `tRNA5.c.cm` against `mrum-genome.fa`.

`5S_rRNA.sto` The Rfam 10.1 5S ribosomal RNA (RF00001) "seed" alignment.

`5S_rRNA.c.cm` A CM file built from `5S_rRNA.sto` using `cmbuild` and calibrated using `cmcalibrate`.

`Cobalamin.sto` The Rfam 10.1 Cobalamin riboswitch (RF00174) "seed" alignment.

`Cobalamin.c.cm` A CM file built from `Cobalamin.sto` using `cmbuild` and calibrated using `cmcalibrate`.

minifam.cm A CM file including three calibrated CMs. This is actually just a concatenation of the files tRNA5.c.cm, 5S_rRNA.c.cm and Cobalamin.c.cm.

minifam.i1{m,i,f,p} Binary compressed files corresponding to minifam.cm, produced by cmpress.

metag-example.fa A FASTA sequence file containing 3 sequences hand-selected from a metagenomics dataset (Tringe et al., 2005), used for demonstrating cmscan.

minifam-metag.out An example cmscan output file, obtained by searching minifam.cm against metag-example.fa.

mrum-tRNAs10.fa A FASTA sequence file containing 10 tRNAs predicted using cmsearch in the *M. ruminantium* genome.

mrum-tRNAs10.out An example cmalign output alignment, obtained by aligning the sequences in mrum-tRNAs10.fa to the model in tRNA5.c.cm with cmalign.

Cobalamin.fa A FASTA sequence file containing 1 cmscan-predicted Cobalamin riboswitch extracted from metag-example.fa.

tRNA5-noss.sto A Stockholm alignment file identical to tRNA5.sto except without secondary structure annotation. Used to demonstrate HMM searches for models without secondary structure.

tRNA5-hand.sto A Stockholm alignment file identical to tRNA5.sto except it includes column reference annotation. Used to demonstrate expert annotation of model positions with cmbuild --hand.

tRNA5-hand.c.cm A CM file built from tRNA5-hand.sto with cmbuild and calibrated with cmcalibrate.

Searching a sequence database with a single covariance model

Step 1: build a covariance model with cmbuild

Infernal starts with a multiple sequence alignment file that you provide. It must be in Stockholm format and must include consensus secondary structure annotation. The file tutorial/tRNA5.sto is an example of a simple Stockholm file. It is shown below, with a secondary structure of the first sequence shown to the right for reference (yeast Phe tRNA, labeled as "tRNA1" in the file):

```
# STOCKHOLM 1.0

tRNA1            GCGGAUUUAGCUCAGUUGGG.AGAGCGCCAGACUGAAGAUCUGGAGGUCC
tRNA2            UCCGAUAUAGUGUAAC.GGCUAUCACAUCACGCUUUCACCGUGGAGA.CC
tRNA3            UCCGUGAUAGUUUAAU.GGUCAGAAUGGGCGCUUGUCGCGUGCCAGA.UC
tRNA4            GCUCGUAUGGCGCAGU.GGU.AGCGCAGCAGAUUGCAAAUCUGUUGGUCC
tRNA5            GGGCACAUGGCGCAGUUGGU.AGCGCGCUUCCCUUGCAAGGAAGAGGUCA
#=GC SS_cons     <<<<<<<..<<<<.........>>>>.<<<<<.......>>>>>.....<

tRNA1            UGUGUUCGAUCCACAGAAUUCGCA
tRNA2            GGGGUUCGACUCCCCGUAUCGGAG
tRNA3            GGGGUUCAAUUCCCCGUCGCGGAG
tRNA4            UUAGUUCGAUCCUGAGUGCGAGCU
tRNA5            UCGGUUCGAUUCCGGUUGCGUCCA
#=GC SS_cons     <<<<.......>>>>>>>>>>>>.
//
```

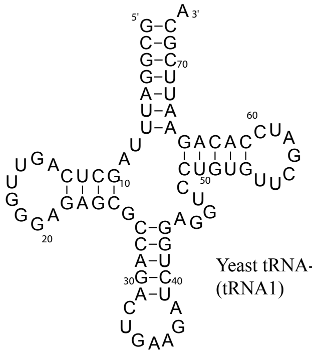

Yeast tRNA-Phe (tRNA1)

This is a simple example of a multiple RNA sequence alignment with secondary structure annotation, in *Stockholm format*. Stockholm format, the native alignment format used by HMMER and Infernal and the Pfam and Rfam databases, is documented in detail later in the guide in section 9.

For now, what you need to know about the key features of the input file is:

- The alignment is in an interleaved format. Lines consist of a name, followed by an aligned sequence; long alignments are split into blocks separated by blank lines.
- Each sequence must have a unique name that has zero spaces in it. (This is important!)
- For residues, any one-letter IUPAC nucleotide code is accepted, including ambiguous nucleotides. Case is ignored; residues may be either upper or lower case.
- Gaps are indicated by the characters `.`, `_`, -, or `~`. (Blank space is not allowed.)
- A special line starting with `#=GC SS_cons` indicates the secondary structure consensus. Gap characters annotate unpaired (single-stranded) columns. Base pairs are indicated by any of the following pairs: `<>`, `()`, `[]`, or {}. No pseudoknots are allowed; the open/close-brackets notation is only unambiguous for strictly nested base-pairing interactions. For more on secondary structure annotation see the WUSS format description in section 9.
- The alignment begins with the special tag line `# STOCKHOLM 1.0`, and ends with `//`. Stockholm alignments can be concatenated to create an alignment database flatfile containing many alignments.

The `cmbuild` command builds a covariance model from an alignment (or CMs for each of many alignments in a Stockholm file), and saves the CM(s) in a file. For example, type:

`> cmbuild tRNA5.cm tutorial/tRNA5.sto`

and you'll see some output that looks like:

```
# cmbuild :: covariance model construction from multiple sequence alignments
# INFERNAL 1.1.2 (June 2016)
# Copyright (C) 2016 Howard Hughes Medical Institute.
# Freely distributed under a BSD open source license.
# - - - - - - - - - - - - - - - - - - - - - - - - - - - - - - - - - - - -
# CM file:                                    tRNA5.cm
# alignment file:                             tutorial/tRNA5.sto
# - - - - - - - - - - - - - - - - - - - - - - - - - - - - - - - - - - - -
#                                                               rel entropy
#                                                               -----------
# idx    name                     nseq eff_nseq   alen  clen  bps bifs    CM   HMM description
# ------ -------------------- -------- -------- ------ ----- ---- ---- ----- ----- -----------
       1 tRNA5                       5     3.73     74    72   21    2 0.783 0.489
#
# CPU time: 0.29u 0.00s 00:00:00.28 Elapsed: 00:00:00.30
```

If your input file had contained more than one alignment, you'd get one line of output for each model. The information on these lines is almost self-explanatory. The `tRNA5` alignment consisted of 5 sequences with 74 aligned columns. Infernal turned it into a model of 72 consensus positions, which means it defined 2 gap-containing alignment columns to be insertions relative to consensus. The 5 sequences were only counted as an "effective" total sequence number (`eff_nseq`) of 3.73. The model includes 21 basepairs and 2 bifurcations. The model ended up with a relative entropy per position (`rel entropy, CM`; information content) of 0.783 bits. If the secondary structure information of the model were ignored the relative entropy per position (`rel entropy, HMM`) would be 0.489 bits. This output format is rudimentary. Infernal knows quite a bit more information about what it's done to build this CM, but it's not displaying it. You don't need to know more to be able to use the model, so we can press on here. Model construction is described in more detail in section 5.

The new CM was saved to `tRNA5.cm`. You can look at it (e.g. `> more tRNA5.cm`) if you like, but it isn't really designed to be human-interpretable. You can treat `.cm` files as compiled models of your RNA alignment. The Infernal ASCII save file format is defined in Section 9.

Step 2: calibrate the model with cmcalibrate

The next step is to "calibrate" the model. This step must be performed prior to using your model for database searches with `cmsearch` or `cmscan`. In this step, statistical parameters necessary for reporting E-values

(expectation values) are estimated and stored in the CM file. When `cmsearch` or `cmscan` is later used for a database search and a hit with score x is found, the E-value of that hit is the number of hits expected to score x or more just by chance (given the size of the search you're performing).

Importantly, if you're not going to use a model for database search, there is no need to calibrate it. For example, if you are only going to use a model to create structurally annotated multiple alignments of a large family like small subunit ribosomal RNA, don't waste time calibrating it. `cmsearch` and `cmscan` are the only Infernal programs that use E-values, so if you're not going to use them then don't calibrate your model.

Unfortunately, CM calibration takes a long time because fairly long random sequences must be searched to determine the expected distribution of hit scores against nonhomologous sequences, and none of the search acceleration heuristics described in section 4 can be used because they rely on primary sequence similarity which is absent in random sequence.

The amount of time required for calibration varies widely, but depends mainly on the size of the RNA family being modeled. So you can know what kind of a wait you're in for, the `cmcalibrate` has a `--forecast` option which reports an estimate of the running time. To get an estimate for the tRNA model, do:

`> cmcalibrate --forecast tRNA5.cm`

You should see something like this:

```
# cmcalibrate :: fit exponential tails for CM E-values
# INFERNAL 1.1.2 (June 2016)
# Copyright (C) 2016 Howard Hughes Medical Institute.
# Freely distributed under a BSD open source license.
# - - - - - - - - - - - - - - - - - - - - - - - - - - - - - - - - - - - -
# CM file:                                    tRNA5.cm
# forecast mode (no calibration):             on
# - - - - - - - - - - - - - - - - - - - - - - - - - - - - - - - - - - - -
#
# Forecasting running time for CM calibration(s) on 24 cpus:
#
#                          predicted
#                       running time
# model name            (hr:min:sec)
# --------------------  ------------
  tRNA5                     00:01:00
#
# CPU time: 0.15u 0.00s 00:00:00.15 Elapsed: 00:00:00.16
[ok]
```

The header comes first, telling you what program you ran, on what file and with what options. This calibration will use 24 CPUs, your output may vary depending on how many cores you have available on the machine you're using. (If you are planning to use MPI to parallelize the calibration (see the Installation section), you can specify the number of CPUs for the time estimate as `<n>` with the `--nforecast <n>` option.) Using 24 CPUs, `cmcalibrate` estimates the time required for calibration on the machine I'm using at about one minute.

Feel free to perform the calibration yourself if you'd like (with the command `cmcalibrate tRNA5.cm`). However, we've included the file `tRNA5.c.cm`, an already calibrated version of `tRNA5.cm`, for you to use if you don't want to wait. To use this calibrated model, copy over the `tRNA5.cm` file you just made with the calibrated version:

`> cp tutorial/tRNA5.c.cm tRNA5.cm`

Step 3: search a sequence database with cmsearch

You can now use your tRNA model to search for tRNA homologs in a database. The file `mrum-genome.fa` is the genome sequence of the archaeon *Methanobrevibacter rumanitium* (accession: `NC_13790.1`). We'll use this file as our database. To perform the search:

`> cmsearch tRNA5.cm tutorial/mrum-genome.fa`

As before, the first section is the header, telling you what program your ran, on what, and with what options:

```
# cmsearch :: search CM(s) against a sequence database
# INFERNAL 1.1.2 (June 2016)
# Copyright (C) 2016 Howard Hughes Medical Institute.
# Freely distributed under a BSD open source license.
# - - - - - - - - - - - - - - - - - - - - - - - - - - - - - - - - - - - -
# query CM file:                          tRNA5.cm
# target sequence database:               tutorial/mrum-genome.fa
# number of worker threads:               24
# - - - - - - - - - - - - - - - - - - - - - - - - - - - - - - - - - - - -
```

The second section is a list of ranked top hits (sorted by E-value, most significant hit first):

```
 rank     E-value  score  bias  sequence      start     end   mdl trunc   gc  description
 ----   --------- ------ -----  ----------- ------- -------   --- ----- ----  -----------
  (1) !   1.3e-18   71.5   0.0  NC_013790.1  362026  361955 -  cm    no 0.50  Methanobrevibacter ruminantium M1 chromosome
  (2) !   3.3e-18   70.2   0.0  NC_013790.1 2585265 2585193 -  cm    no 0.60  Methanobrevibacter ruminantium M1 chromosome
  (3) !     9e-18   68.8   0.0  NC_013790.1  762490  762562 +  cm    no 0.67  Methanobrevibacter ruminantium M1 chromosome
  (4) !     9e-18   68.8   0.0  NC_013790.1 2041704 2041632 -  cm    no 0.67  Methanobrevibacter ruminantium M1 chromosome
  (5) !   2.5e-17   67.4   0.0  NC_013790.1 2351254 2351181 -  cm    no 0.62  Methanobrevibacter ruminantium M1 chromosome
  (6) !     3e-17   67.2   0.0  NC_013790.1  735136  735208 +  cm    no 0.59  Methanobrevibacter ruminantium M1 chromosome
  (7) !   5.2e-17   66.4   0.0  NC_013790.1 2186013 2185941 -  cm    no 0.53  Methanobrevibacter ruminantium M1 chromosome
  (8) !   1.6e-16   64.8   0.0  NC_013790.1 2350593 2350520 -  cm    no 0.66  Methanobrevibacter ruminantium M1 chromosome
  (9) !   2.8e-16   64.1   0.0  NC_013790.1 2585187 2585114 -  cm    no 0.59  Methanobrevibacter ruminantium M1 chromosome
 (10) !   9.1e-16   62.5   0.0  NC_013790.1  662185  662259 +  cm    no 0.61  Methanobrevibacter ruminantium M1 chromosome
 (11) !   1.2e-15   62.1   0.0  NC_013790.1  360887  360815 -  cm    no 0.55  Methanobrevibacter ruminantium M1 chromosome
 (12) !   1.6e-15   61.7   0.0  NC_013790.1 2350984 2350911 -  cm    no 0.53  Methanobrevibacter ruminantium M1 chromosome
 (13) !   3.3e-15   60.7   0.0  NC_013790.1 2186090 2186019 -  cm    no 0.54  Methanobrevibacter ruminantium M1 chromosome
 (14) !   4.1e-15   60.4   0.0  NC_013790.1 2680159 2680233 +  cm    no 0.67  Methanobrevibacter ruminantium M1 chromosome
 (15) !   7.9e-15   59.5   0.0  NC_013790.1 2749945 2749874 -  cm    no 0.53  Methanobrevibacter ruminantium M1 chromosome
 (16) !   7.9e-15   59.5   0.0  NC_013790.1 2749839 2749768 -  cm    no 0.53  Methanobrevibacter ruminantium M1 chromosome
 (17) !   9.8e-15   59.2   0.0  NC_013790.1  361676  361604 -  cm    no 0.51  Methanobrevibacter ruminantium M1 chromosome
 (18) !     1e-14   59.2   0.0  NC_013790.1 2585073 2584999 -  cm    no 0.60  Methanobrevibacter ruminantium M1 chromosome
 (19) !   1.1e-14   59.1   0.0  NC_013790.1 2130422 2130349 -  cm    no 0.59  Methanobrevibacter ruminantium M1 chromosome
 (20) !   1.2e-14   58.9   0.0  NC_013790.1  546056  545947 -  cm    no 0.61  Methanobrevibacter ruminantium M1 chromosome
 (21) !   3.9e-14   57.3   0.0  NC_013790.1  361915  361844 -  cm    no 0.42  Methanobrevibacter ruminantium M1 chromosome
 (22) !   5.1e-14   57.0   0.0  NC_013790.1   97724   97795 +  cm    no 0.49  Methanobrevibacter ruminantium M1 chromosome
 (23) !   6.1e-14   56.7   0.0  NC_013790.1 2350717 2350646 -  cm    no 0.68  Methanobrevibacter ruminantium M1 chromosome
 (24) !     8e-14   56.3   0.0  NC_013790.1 1873887 1873815 -  cm    no 0.64  Methanobrevibacter ruminantium M1 chromosome
 (25) !   1.4e-13   55.6   0.0  NC_013790.1  360730  360659 -  cm    no 0.40  Methanobrevibacter ruminantium M1 chromosome
 (26) !   3.5e-13   54.3   0.0  NC_013790.1 2680310 2680384 +  cm    no 0.52  Methanobrevibacter ruminantium M1 chromosome
 (27) !   3.6e-13   54.3   0.0  NC_013790.1 2664806 2664732 -  cm    no 0.60  Methanobrevibacter ruminantium M1 chromosome
 (28) !   3.6e-13   54.3   0.0  NC_013790.1  361061  360989 -  cm    no 0.41  Methanobrevibacter ruminantium M1 chromosome
 (29) !   7.5e-13   53.3   0.0  NC_013790.1 2130335 2130262 -  cm    no 0.55  Methanobrevibacter ruminantium M1 chromosome
 (30) !   7.6e-13   53.3   0.0  NC_013790.1 2151672 2151745 +  cm    no 0.65  Methanobrevibacter ruminantium M1 chromosome
 (31) !   2.9e-12   51.4   0.0  NC_013790.1  319297  319370 +  cm    no 0.62  Methanobrevibacter ruminantium M1 chromosome
 (32) !   3.7e-12   51.1   0.0  NC_013790.1  361753  361679 -  cm    no 0.55  Methanobrevibacter ruminantium M1 chromosome
 (33) !   3.8e-12   51.1   0.0  NC_013790.1  360983  360912 -  cm    no 0.50  Methanobrevibacter ruminantium M1 chromosome
 (34) !   5.9e-12   50.5   0.0  NC_013790.1  361456  361383 -  cm    no 0.50  Methanobrevibacter ruminantium M1 chromosome
 (35) !   7.4e-12   50.1   0.0  NC_013790.1  362798  362727 -  cm    no 0.51  Methanobrevibacter ruminantium M1 chromosome
 (36) !   8.7e-12   49.9   0.0  NC_013790.1  917722  917793 +  cm    no 0.61  Methanobrevibacter ruminantium M1 chromosome
 (37) !   1.1e-11   49.7   0.0  NC_013790.1 2583869 2583798 -  cm    no 0.51  Methanobrevibacter ruminantium M1 chromosome
 (38) !   1.3e-11   49.4   0.0  NC_013790.1  362324  362252 -  cm    no 0.51  Methanobrevibacter ruminantium M1 chromosome
 (39) !   1.3e-11   49.3   0.0  NC_013790.1  360811  360740 -  cm    no 0.42  Methanobrevibacter ruminantium M1 chromosome
 (40) !   4.3e-11   47.7   0.0  NC_013790.1 1160526 1160609 +  cm    no 0.60  Methanobrevibacter ruminantium M1 chromosome
 (41) !   9.8e-11   46.6   0.0  NC_013790.1  362403  362331 -  cm    no 0.49  Methanobrevibacter ruminantium M1 chromosome
 (42) !   1.1e-10   46.5   0.0  NC_013790.1 2327124 2327042 -  cm    no 0.63  Methanobrevibacter ruminantium M1 chromosome
 (43) !   1.2e-10   46.4   0.0  NC_013790.1  995344  995263 -  cm    no 0.49  Methanobrevibacter ruminantium M1 chromosome
 (44) !   2.3e-10   45.5   0.0  NC_013790.1  256772  256696 -  cm    no 0.57  Methanobrevibacter ruminantium M1 chromosome
 (45) !   2.5e-10   45.3   0.0  NC_013790.1 2584830 2584758 -  cm    no 0.64  Methanobrevibacter ruminantium M1 chromosome
 (46) !   6.1e-10   44.1   0.0  NC_013790.1 2351071 2350997 -  cm    no 0.59  Methanobrevibacter ruminantium M1 chromosome
 (47) !   6.5e-10   44.0   0.0  NC_013790.1  362552  362482 -  cm    no 0.55  Methanobrevibacter ruminantium M1 chromosome
 (48) !   5.2e-09   41.2   0.0  NC_013790.1 1064775 1064858 +  cm    no 0.63  Methanobrevibacter ruminantium M1 chromosome
 (49) !   1.2e-08   40.0   0.0  NC_013790.1  361222  361150 -  cm    no 0.45  Methanobrevibacter ruminantium M1 chromosome
 (50) !   1.2e-08   40.0   0.0  NC_013790.1  361369  361297 -  cm    no 0.60  Methanobrevibacter ruminantium M1 chromosome
 (51) !   4.8e-08   38.1   0.0  NC_013790.1  361596  361513 -  cm    no 0.61  Methanobrevibacter ruminantium M1 chromosome
 (52) !   3.2e-07   35.5   0.0  NC_013790.1 1913310 1913227 -  cm    no 0.64  Methanobrevibacter ruminantium M1 chromosome
 (53) !   2.6e-06   32.7   0.0  NC_013790.1  363464  363381 -  cm    no 0.51  Methanobrevibacter ruminantium M1 chromosome
 (54) !     3e-06   32.5   0.0  NC_013790.1 2584954 2584872 -  cm    no 0.58  Methanobrevibacter ruminantium M1 chromosome
 ------ inclusion threshold ------
 (55) ?     0.027   20.0   0.0  NC_013790.1  363803  363716 -  cm    no 0.50  Methanobrevibacter ruminantium M1 chromosome
 (56) ?       3.4   13.4   0.0  NC_013790.1  984373  984304 -  cm    no 0.53  Methanobrevibacter ruminantium M1 chromosome
```

The first number is the rank of each hit[1]. Next comes either a `!` or `?` symbol and then the *E-value* of the hit. The E-value is the statistical significance of the hit: the number of hits we'd expect to score this highly in a database of this size (measured by the total number of nucleotides) if the database contained only nonhomologous random sequences. The lower the E-value, the more significant the hit. The `!` or `?` that precedes the E-value indicates whether the hit does (`!`) or does not (`?`) satisfy the inclusion threshold for the search. Inclusion thresholds are used to determine what matches should be considered to be "true", as opposed to reporting thresholds that determine what matches will be reported (often including the top of the noise, so you can see what interesting sequences might be getting tickled by your search). By default, inclusion thresholds usually require an E-value of 0.01 or less, and reporting E-value thresholds are set to 10.0, but these can be changed (see the manual page for `cmsearch` toward the end of guide).

The E-value is based on the *bit score*, which is in the next column. This is the log-odds score for the hit. Some people like to see a bit score instead of an E-value, because the bit score doesn't depend on the size of the sequence database, only on the covariance model and the target sequence.

The next number, the *bias*, is a correction term for biased sequence composition that has been applied to the sequence bit score. Infernal uses an alternative null model we call *null3*, described more in section 4, to determine the bias bit score correction. The bias correction is often very small and is only reported to one decimal place, after rounding. For all hits in this example search the bias column reads 0.0 bits, indicating that the correction is less than 0.05 bits. On very biased sequences this correction can become significant and is helpful for lowering the score of high-scoring false positives that achieve high scores solely due to their biased composition.

Next comes the target sequence name the hit is in, and the start and end positions of the hit within the sequence. Hits can occur on either the top (Watson) or bottom (Crick) strand of the target sequence[2], so the start position may be less than (if hit is on the top strand) or greater than (if hit is on the bottom strand) the end position. After the end position, comes a single `+` or `-` symbol, indicating whether the hit is on the top (`+`) or bottom (`-`) strand (solely for convenience - so you don't have to look at the start and end positions to determine the strand the hit is on).

After the strand symbol comes the model field, which indicates whether the hit was found using either the CM (`cm`) or a profile HMM built from the CM (`hmm`). This field is necessary because for models with zero basepairs, `cmsearch` (and `cmscan`) use a profile HMM instead of a CM for final hit scoring. This is done for reasons of speed and efficiency, because profile HMM algorithms are more efficient than CM ones and a CM with zero basepairs is essentially equivalent to a profile HMM. In this example, since our tRNA model does include basepairs, `cmsearch` used a CM to score all hits and so all hits have `cm` for this column. There's an example later in the tutorial of hits found with a profile HMM.

The next column indicates whether the hit is *truncated* or not. Infernal uses special versions of its CM dynamic programming algorithms to allow detection of structural RNAs that have been truncated due to missing data at the beginning and/or end of a target sequence. Truncated hits are most common in databases that include single reads from shotgun sequencing projects. Since our database is a complete genome, we don't expect any hits to be truncated due to missing data. For all hits the "trunc" column reads "no" indicating that, as expected, none of the hits are truncated. There are examples of truncated hits in the next exercise which uses `cmscan`. Section 4 describes how Infernal detects and aligns truncated hits in more detail.

The next column reports the GC fraction of the hit. This is the fraction of residues in the target sequence hit that are either G or C residues. The GC fraction is included as an additional indication of the level of sequence bias of the hit. Some expert users may be aided by this number when deciding if they believe a hit is a real homolog or a false positive.

Finally comes the description of the sequence, if any. This description is propogated from the input target sequence file.

[1] Ranks of hits are in parantheses to make it easy to jump to/from an entry in the hit list and the hit alignment section, described later.

[2] You can search either only the top strand with the `--toponly` or bottom strand with the `--bottomonly` options to `cmsearch` and `cmscan`.

After the hit list comes the hit alignments section. Each hit in the hit list will have a corresponding entry in this section, in the same order. As an illustrative example, let's take a look at hit number 43. First, take a look at the first four lines for this hit:

```
>> NC_013790.1  Methanobrevibacter ruminantium M1 chromosome, complete genome
 rank     E-value  score  bias mdl mdl from   mdl to       seq from      seq to       acc trunc   gc
 ----   --------- ------ ----- --- -------- --------    ----------- -----------      ---- ----- ----
 (43) !   1.2e-10   46.4   0.0  cm        1       72 []      995344      995263 - .. 0.93    no 0.49
```

The first line of each hit alignment begins with `>>` followed by a single space, the name of the target sequence, then two spaces and the description of the sequence, if any. Next comes a set of tabular fields that is partially redundant with the information in the hit list. The first five columns are the same as in the hit list. The next column reports the type of model used for the alignment, as described above for the hit list. The next four columns report the boundaries of the alignment with respect to the query model ("mdl from" and "mdl to") and the target sequence ("seq from" and "seq to"). Following the "seq to" column is a `+` or `-` symbol indicating whether the hit is on the top (`+`) or bottom (`-`) strand.

It's not immediately easy to tell from the "to" coordinate whether the alignment ended internally in the query model or target sequence, versus ran all the way (as in a full-length global alignment) to the end(s). To make this more readily apparent, with each pair of query and target endpoint coordinates, there's also a little symbology. For the normal case of a non-truncated hit: `..` means both ends of the alignment ended internally, and `[]` means both ends of the alignment were full-length flush to the ends of the query or target, and `[.` and `.]` mean only the left (5') or right (3') end was flush/full length. For truncated hits, the symbols are the same except that either the first and/or the second symbol will be a `~` for the query and target. If the first symbol is `~` then the left end of the alignment is truncated because the 5' end of the hit is predicted to be missing (extend beyond the beginning of the target sequence). Similarly, if the second symbol is `~` then the right end of the alignment is truncated because the 3' end of the hit is predicted to be missing (extend beyond the end of the target sequence). These two symbols occur just after the "mdl to" column for the query, and after the strand `+` or `-` symbol for the target sequence.

The next column is labeled "acc" and is the average posterior probability of the aligned target sequence residues; effectively, the expected accuracy per residue of the alignment.

The final two columns indicate whether the hit is truncated or not and the GC fraction of the hit. These are redundant with the columns of the same name in the hit list, described above.

Next comes the alignment display. This is an "optimal posterior accuracy" alignment (Holmes, 1998), which means it is the alignment with the maximal summed posterior probability of all aligned residues. Take a look at the alignment for hit number 43:

```
                               v         v                                                            NC
                   (((((((,,<<<<______.._>>>>,<<<<<_______>>>>>,,,........,,<<<<<_______>>>>>))))))): CS
      tRNA5      1 gCcggcAUAGcgcAgUGGu..AgcgCgccagccUgucAagcuggAGg........UCCgggGUUCGAUUCcccGUgccgGca 72
                   :::G:CAUAGCG AG GGU  A CGCG:CAG:CU +++A:CUG: G+        UC:GGGGUUCGA UCCCC:UG:C:::A
NC_013790.1 995344 AGAGACAUAGCGAAGCGGUcaAACGCGGCAGACUCAAGAUCUGUUGAuuaguucuUCAGGGGUUCGAAUCCCCUUGUCUCUA 995263
                   **********************************************9444444445************************** PP
```

The alignment contains six lines. Start by looking at the second line which ends with `CS`. The line shows the predicted secondary structure of the target sequence. The format is a little fancier and more informative than the simple least-common-denominator format we used in the input alignment file. It's designed to make it easier to see the secondary structure by eye. The format is described in detail later (see WUSS format in section 9); for now, here's all you need to know. Basepairs in simple stem loops are annotated with `<>` characters. Basepairs enclosing multifurcations (multiple stem loops) are annotated with `()`, such as the tRNA acceptor stem in this example. In more complicated structures, `[]` and {} annotations also show up, to reflect deeper nestings of multifurcations. For single stranded residues, `_` characters mark hairpin loops; `-` characters mark interior loops and bulges; `,` characters mark single-stranded residues in multifurcation loops; and `:` characters mark single stranded residues external to any secondary structure. Insertions relative to this consensus are annotated by a `.` character.

The line above the `CS` line ends with `NC` and marks negative scoring non-canonical basepairs in the alignment with a `v` character. All other positions of the alignment will be blank[3] More specifically, the follow-

[3]For anyone trying to parse this output, this means it is possible for this line to be completely blank except for the `NC` trailer.

ing ten types of basepairs which are assigned a negative score by the model at their alignment positions will be marked with a v: A:A, A:C, A:G, C:A, C:C, C:U, G:A, G:G, U:U, and U:C. The NC annotation makes it easy to quickly identify suspicious basepairs in a hit. Importantly, the NC annotation will only be present in CM hit alignments ("mdl" column reads "cm") and will be absent in HMM hit alignments ("mdl" column reads "hmm") because basepairs are not scored by an HMM.

The third line shows that consensus of the query model. The highest scoring residue sequence is shown. Upper case residues are highly conserved. Lower case residues are weakly conserved or unconserved. Dots (.) in this line indicate insertions in the target sequence with respect to the model.

The fourth line shows where the alignment score is coming from. For a consensus basepair, if the observed pair is the highest-scoring possible pair according to the consensus, both residues are shown in upper case; if a pair has a score of ≥ 0, both residues are annotated by : characters (indicating an acceptable compensatory basepair); else, there is a space, indicating that a negative contribution of this pair to the alignment score. Note that the NC line will only mark a subset of these negative scoring pairs with a v, as discussed above. For a single-stranded consensus residue, if the observed residue is the highest scoring possibility, the residue is shown in upper case; if the observed residue has a score of ≥ 0, a + character is shown; else there is a space, indicating a negative contribution to the alignment score. Importantly, for HMM hits ("mdl" column reads "hmm"), *all* positions are considered single stranded, since an HMM scores each half of a basepair independently.

The fifth line, beginning with NC_013790.1 is the target sequence. Dashes (-) in this line indicate deletions in the target sequence with respect to the model.

The bottom line is new to version 1.1 of Infernal. This represents the posterior probability (essentially the expected accuracy) of each aligned residue. A 0 means 0-5%, 1 means 5-15%, and so on; 9 means 85-95%, and a * means 95-100% posterior probability. You can use these posterior probabilities to decide which parts of the alignment are well-determined or not. You'll often observe, for example, that expected alignment accuracy degrades around locations of insertion and deletion, which you'd intuitively expect.

Alignments for some searches may be formatted slightly differently than this example. Longer alignments to longer models will be broken up into blocks of six lines each - this alignment was short enough to be entirely contained within a single block. If your model was built with the --hand option in cmbuild, then an additional line will be included in each block, with RF annotation. If the model used for the alignment was an HMM (the "mdl" column reads "hmm") then the NC line will be absent from each alignment block. We'll see example of all three of these cases later in the tutorial.

After hit number 43, there's 13 more hit alignments for hits number 44 through 56.

Finally, at the bottom of the file, you'll see some summary statistics. For example, at the bottom of the tRNA search output, you'll find something like:

```
Internal CM pipeline statistics summary:
----------------------------------------
Query model(s):                                                    1  (72 consensus positions)
Target sequences:                                                  1  (5874406 residues searched)
Target sequences re-searched for truncated hits:                   1  (360 residues re-searched)
Windows   passing  local HMM SSV           filter:             11205  (0.2116); expected (0.35)
Windows   passing  local HMM Viterbi       filter:                    (off)
Windows   passing  local HMM Viterbi  bias filter:                    (off)
Windows   passing  local HMM Forward       filter:               136  (0.002693); expected (0.005)
Windows   passing  local HMM Forward  bias filter:               133  (0.002623); expected (0.005)
Windows   passing glocal HMM Forward       filter:                84  (0.001951); expected (0.005)
Windows   passing glocal HMM Forward  bias filter:                84  (0.001951); expected (0.005)
Envelopes passing glocal HMM envelope defn filter:                98  (0.001318); expected (0.005)
Envelopes passing  local CM  CYK           filter:                60  (0.0007629); expected (0.0001)
Total CM hits reported:                                           56  (0.0007205); includes 0 truncated hit(s)

# CPU time: 2.01u 0.05s 00:00:02.05 Elapsed: 00:00:00.49
//
[ok]
```

This gives you some idea of what's going on in Infernal's acceleration pipeline. You've got one query CM, and the database has one target sequence. The search examined 5,874,406 residues, even though the actual target sequence length is only half that, because both the top and bottom strand of each target is

searched. 360 of those residues were searched more than once in an effort to find truncated hits. Ignore this for the moment, we'll revisit this later after discussing the filter pipeline (in the subsection entitled "Truncated RNA detection" below).

Each sequence goes through a multi-stage filter pipeline of four scoring algorithms called SSV, Viterbi, Forward[4], and CYK in order of increasing sensitivity and increasing computational requirement. The filter pipeline is the topic of section 4 of this guide but briefly, SSV, Viterbi and Forward are profile HMM algorithms which are more efficient than CM algorithms. These three algorithms are the same ones used by HMMER3 and are the main reason that version 1.1 of Infernal is so much faster than previous versions. For these HMM stages, Infernal uses a filter profile HMM that was constructed simultaneously with the CM, from the same training alignment in `cmbuild`, and stored in the CM file. CYK is a CM scoring algorithm, so it's slow, but it is accelerated using banded dynamic programming with bands derived from an HMM alignment. Subsequences that survive all filters are finally scored with the CM Inside algorithm, agains using HMM bands. Subsequences that score sufficiently high with Inside are then aligned using the optimal posterior accuracy algorithm and displayed.

The score thresholds for a subsequence surviving each HMM filter stage are dependent on the search space size (sequence database size for `cmsearch`). This differs from HMMER3 which always uses the same filter thresholds. In general, the larger the search space the more strict the thresholds are, because a hit must have a higher bit score to have a significant E-value. In this case, the database is relatively small so the filter thresholds have been set relatively loosely. The SSV filter has been configured to allow subsequences with a P-value of ≤ 0.35 through the SSV score filter (thus, if the database contained no homologs and P-values were accurately calculated, the highest scoring 35% of the residues will pass the filter). Here, about 21% of the database in 11,205 separate windows got through the SSV filter. For a database of this size, the local Viterbi filter is turned off. The local Forward filter is set to allow an expected 0.5% of the database survive. Here about 0.3% survives in 136 windows. Next, each surviving window is checked to see if the target sequence is "obviously" so biased in its composition that it's unlikely to be a true homolog. This is called the "bias filter"[5] and applying a bit score correction to previous filter's score for each window and recomputing the P-value. Three of the 136 windows fail to pass the local Forward bias filter stage. Next, the Forward algorithm is used to score each window again, but this time with the HMM configured in glocal mode requiring a full length alignment to the model[6] As with the local stage, an expected 0.5% of the database is expected to survive. In this case, 84 of the 134 windows, comprising about 0.2% of the database, survive. The bias filter is run again, this time applying a correction to the glocal Forward scores. For this search, 0 windows are removed at this stage. The envelope definition stage is next. This stage is very similar to the HMMER3 domain definition stage, with the difference that the HMM is configured in glocal rather than local mode. In this stage, the Forward and Backward algorithms are used to identify zero or more hit envelopes in each window, where each envelope contains one putative hit. Often residues at the beginning and ends of windows are determined to be nonhomologous and are not included in the envelope. In this search, 98 envelopes are defined within the 84 windows. Note that the envelopes comprise only about 70% of the residues from the 84 windows, indicated by the drop of 0.1951% to 0.1318%.

After hit envelopes have been defined with the filter HMM, the two remaining stages of the pipeline use the CM to score both the conserved sequence and structure of each possible hit. In both of these stages, constraints are derived from an HMM alignment of the envelope and enforced as *bands* on the CM dynamic programming matrices (more on this in section 4). In the first CM stage, the CYK algorithm (which is the SCFG analog of the Viterbi HMM algorithm) is used to determine the best scoring maximum likelihood alignment of any subsequence in each envelope to the CM. If this alignment has a P-value of ≤ 0.0001 then the envelope survives to the final round. The envelopes passed to the final stage may be shorter than those examined during the CYK stage. Specifically, envelopes are redefined as starting and ending at the first

[4]Actually two separate Forward based filters are used, the first with the profile HMM in local mode and the next with the profile HMM in global mode. There's more detail on this in section 4.

[5]There's also a bias filter step used in the local Viterbi filter stage, when it is used.

[6]The use of glocal Forward is another important difference between Infernal and HMMER3's (v3.0) pipeline. HMMER v3.0 only uses local HMM algorithms.

and final residues for which at least one alignment exists with a P-value ≤ 0.001.

In the final round, the Inside algorithm (the SCFG analog of the HMM Forward algorithm) is used to define final hit boundaries and scores. Hits with scores above the reporting threshold were output, as described above. In this search there were 56 such hits.

Finally, the running time of the search is reported, in CPU time and elapsed time. This search took about half a second (wall clock time) (running on twenty four cores).

Truncated RNA detection

Now, we come back to the topic of truncated hit detection. As briefly mentioned above, the pipeline statistics summary from our search above reported that 360 residues were re-searched for truncated hits. Infernal explicitly looks at the 5' and 3' ends of target sequences using specialized algorithms for detection of truncated hits, in which part of the 5' and/or 3' end of the actual full length sequence from the source organism's full genomic context is missing in the input target sequence. Truncated hits will be most common in sequence files consisting of unassembled sequencing reads. In our search of the full archaeal genome above, no truncated hits were found. However, there is an example of a truncated hit in the `cmscan` tutorial section below in a sequence from a metagenomics sequencing survey.

Special dynamic programming algorithms are required for truncated hit detection (Kolbe and Eddy, 2009), so sequences must be re-searched for truncated hits after they are initially searched for standard (non-truncated) hits. However, only the sequence ends must be re-searched because Infernal assumes that only hits at sequence terminii might be truncated. This is why our search above reported that only 360 residues were re-searched for truncated hits. For each model, an expected maximum length of a hit[7] is used to define the window length at the beginning and end of each sequence which must be re-searched for truncated hits. For our tRNA model the maximum expected length is 90, so exactly 360 residues were re-searched: the first and final 90 residues on the top strand, and the first and final 90 residues on the bottom strand.

There is one more aspect of truncated hit detection that is important to mention here. Because Infernal expects that hit truncation only occurs at the ends of sequences, 5' truncated hits are forced to start at the first nucleotide of a sequence, 3' truncated hits are forced to end at the final nucleotide of the sequence, and 5' *and* 3' truncated hits are forced to include the full sequence.

The annotation of truncated hits in `cmsearch` output is slightly different than for standard (non-truncated) hits. An example is included in the next section below.

Searching a CM database with a query sequence

The `cmscan` program is for annotating all the different known/detectable RNAs in a given sequence. It takes a single query sequence and a CM database as input. The CM database might be Rfam, for example, or another collection of your choice. `cmscan` is new to version 1.1 of Infernal. It is designed to be useful for sequence datasets from RNA-Seq experiments or metagenomics surveys, for which one wants to know what families of RNAs are represented in the sequence dataset.

Step 1: create an CM database flatfile

A CM "database" flatfile is simply a concatenation of individual CM files. To create a database flatfile, you can either build and calibrate individual CM files and concatenate them, or you can concatenate Stockholm alignments and use `cmbuild` to build a CM database of all of them in one command, and then calibrate that database with `cmcalibrate`. Importantly, `cmscan` can only be used with calibrated CMs.

[7]The expected maximum hit length is defined as the maximum of 1.25 times the consensus length of the model and the W parameter computed with the QDB algorithm (Nawrocki and Eddy, 2007). W is computed based on the transition probabilities of the model by `cmbuild` and stored in the CM file.

Let's create a tiny database called `minifam.cm` containing the tRNA model we've been working with, a 5S ribosomal RNA model, and a Cobalamin riboswitch model. To save you time, calibrated versions of the 5S and Cobalamin models are included in the `tutorial/` directory in the files `5S_rRNA.c.cm`, and `Cobalamin.c.cm`. These files were created using `cmbuild` and `cmcalibrate` from the Rfam 10.1 seed alignments for 5S_rRNA (RF00001) and Cobalamin (RF00174), provided in `tutorial/5S_rRNA.sto` and `tutorial/Cobalamin.sto`. The third model is the tRNA model from earlier in the tutorial (`tRNA5.c.cm`). Feel free to build and calibrate these models yourself if you'd like, but if you'd like to keep moving on with the tutorial, use the pre-calibrated ones. To create the database, simply concatenate the three provided files:

> cat tutorial/tRNA5.c.cm tutorial/5S_rRNA.c.cm tutorial/Cobalamin.c.cm > minifam.cm

Step 2: compress and index the flatfile with cmpress

The `cmscan` program has to read a lot of CMs and their filter HMMs in a hurry, and Infernal's ASCII flatfiles are bulky. To accelerate this, `cmscan` uses binary compression and indexing of the flatfiles. To use `cmscan`, you must first compress and index your CM database with the `cmpress` program:

> cmpress minifam.cm

This will quickly produce:

```
Working...    done.
Pressed and indexed 3 CMs and p7 HMM filters (3 names and 2 accessions).
Covariance models and p7 filters pressed into binary file:  minifam.cm.i1m
SSI index for binary covariance model file:                 minifam.cm.i1i
Optimized p7 filter profiles (MSV part)  pressed into:      minifam.cm.i1f
Optimized p7 filter profiles (remainder) pressed into:      minifam.cm.i1p
```

and you'll see these four new binary files in the directory.

The `tutorial` directory includes a copy of the `minifam.cm` file, which has already been pressed, so there are example binary files `tutorial/minifam.cm.i1{m,i,f,p}` included in the tutorial.

Their format is "proprietary", which is an open source term of art that means both "We haven't found time to document them yet" and "We still might decide to change them arbitrarily without telling you".

Step 3: search the CM database with cmscan

Now we can analyze sequences using our CM database and `cmscan`.

For example, the file `tutorial/metag-example.fa` includes 3 sequences (whole genome shotgun sequencing reads) derived from samples of "whale fall" carcasses in a metagenomics study (Tringe et al., 2005). These three sequences were chosen from the roughly 200,000 in the complete dataset because they include statistically significant hits to the three models in our toy CM database.

To scan the sequences with our database:

> cmscan minifam.cm tutorial/metag-example.fa

The header and the first section of the output will look like:

```
# cmscan :: search sequence(s) against a CM database
# INFERNAL 1.1.2 (June 2016)
# Copyright (C) 2016 Howard Hughes Medical Institute.
# Freely distributed under a BSD open source license.
# - - - - - - - - - - - - - - - - - - - - - - - - - - - - - - - - - - - -
# query sequence file:                   tutorial/metag-example.fa
# target CM database:                    minifam.cm
# number of worker threads:              24
# - - - - - - - - - - - - - - - - - - - - - - - - - - - - - - - - - - - -

Query:       AAGA01015927.1  [L=943]
Description: Metagenome sequence AHAI1002.g1, whole genome shotgun sequence
Hit scores:
 rank     E-value  score  bias  modelname  start    end   mdl trunc   gc  description
 ----   --------- ------ -----  --------- ------ ------   --- ----- ----  -----------
  (1) !   3.3e-19   77.3   0.0  5S_rRNA       59    174 +  cm    no 0.66  5S ribosomal RNA
  (2) !   9.3e-19   62.4   0.0  tRNA5        229    302 +  cm    no 0.62  -
  (3) !     6e-16   53.5   0.0  tRNA5        314    386 +  cm    no 0.59  -
```

`cmscan` has identified three putative RNAs in the first query sequence, one 5S rRNA and two tRNAs. The output fields are in the same order and have the same meaning as in `cmsearch`'s output.

Before we move on, this is a good opportunity to point out an important difference between `cmsearch` and `cmscan` related to the size of the search space (often referred to in the code and in this guide as the Z parameter). The size of the search space for `cmscan` is double the length of the current (single) query sequence (doubled because we're searching both strands) multiplied by the number of models in the CM database (here, 3; for a Rfam search, on the order of 1000). Because each query sequence is probably a different size this means Z changes for each query sequence. In `cmsearch`, the size of the search space is double the summed length of all sequences in the database (again, doubled because both strands are searched). This means that E-values may differ even for the same individual CM vs. sequence comparison, depending on how you do the search. The search space size also affects what filter thresholds `cmsearch` or `cmscan` will use, which is discussed more in section 4.

Now back to the `cmscan` results. What follows the ranked list of three hits are the hit alignments. These are constructed and annotated the same as in `cmsearch`. The 5S alignment is:

```
                            v               v       v         v         v             v           vv                NC
                    (((((((((,,,,<<-<<<<<---<<--<<<<<<______.>>-->>>>-->>---->>>>>-->><<<-<<----<-<<-----<<____>>-- CS
         5S_rRNA   1 gcuuGcggcCAUAccagcgcgaAagcACcgGauCCCAUCc.GaACuCcgAAguUAAGcgcgcUugggCcagggUAGUAcuagGaUGgGuGAcCuC 94
                     :CU:GC:G C UA:C+::G:G+   CACC:GA CCCAU C G ACUC:GAAG  AA C:C::U+G: CC+::G  G:A  + G  GGGU  CC C
  AAGA01015927.1  59 CCUGGCGGCCGUAGCGCGGUGGUCCCACCUGACCCCAUGCcGAACUCAGAAGUGAAACGCCGUAGCGCCGAUG--GUAGUGUG--GGGUCUCCCC 149
                     *************************************************************************..********..********** PP

                        vv         v v          NC
                     --->>->-->>->>>.))))))))): CS
         5S_rRNA  95 cUGggAAgaccagGu.gccgCaagcc 119
                      UG  A: A::AGG   C:GC:AG:C
  AAGA01015927.1 150 AUGCGAG-AGUAGGGaACUGCCAGGC 174
                     **99999.****************** PP
```

After the three sequences, you'll see a pipeline statistics summary report:

```
Internal CM pipeline statistics summary:
----------------------------------------
Query sequence(s):                                               1  (1886 residues searched)
Query sequences re-searched for truncated hits:                  1  (992.0 residues re-searched, avg per model)
Target model(s):                                                 3  (382 consensus positions)
Windows   passing  local HMM SSV           filter:              16  (0.3323); expected (0.35)
Windows   passing  local HMM Viterbi       filter:                  (off)
Windows   passing  local HMM Viterbi  bias filter:                  (off)
Windows   passing  local HMM Forward       filter:               4  (0.09822); expected (0.02)
Windows   passing  local HMM Forward  bias filter:               4  (0.09822); expected (0.02)
Windows   passing glocal HMM Forward       filter:               3  (0.09822); expected (0.02)
Windows   passing glocal HMM Forward  bias filter:               3  (0.09822); expected (0.02)
Envelopes passing glocal HMM envelope defn filter:               4  (0.05189); expected (0.02)
Envelopes passing  local CM  CYK           filter:               4  (0.05189); expected (0.0001)
Total CM hits reported:                                          3  (0.03046); includes 0 truncated hit(s)

# CPU time: 0.15u 0.02s 00:00:00.17 Elapsed: 00:00:00.12
//
```

This report is similar to the one you saw earlier from `cmsearch`, but not identical. One big difference is that `cmscan` will report a summary per query sequence, instead of per query model. In this case, the sequence was 943 residues long, so a total of 1886 residues were searched, since both strands were examined. The next line reports that the average number of residues re-searched for truncated hits per model was 992.0. An average is reported here because remember the number of residues re-searched per model depends on the expected maximum size of a hit, which varies per model, because only sequence terminii are examined for truncated hits (see "Truncated RNA detection" above and section 4). The remainder of the output is the same as in `cmsearch` except that the fractional values are averages per model. For example, for all three models a total of 4 envelopes survived the CYK filter stage, and those surviving envelopes contained 5.189% of the target sequence *on average per model*.

You may have noticed that the expected survival fractions are different than they were in the `cmsearch` example. This is because the P-value filter thresholds are set differently depending on the search space. For this example, the search space (Z) is roughly only 6 Kb (1886 residues in the query sequence multiplied by 3 target models), so the thresholds are set differently than for the `cmsearch` example which had a search space size of roughly 6 Mb. The exact thresholds used for various Z values can be found in Table 1 in section 4.

Truncated hit and local end alignment example

Next, take a look at the `cmscan` output for the second query sequence `AAFY01022046.1`. The bottom strand of this sequence contains one hit to the Cobalamin riboswitch model, which is truncated at the 5' end. Here is the alignment:

```
Hit alignments:
>> Cobalamin  Cobalamin riboswitch
 rank     E-value  score  bias mdl mdl from   mdl to       seq from      seq to       acc trunc   gc
 ----   --------- ------ ----- --- -------- --------    ----------- -----------      ---- ----- ----
  (1) !   6.1e-09   30.0   0.0  cm       32      191 ~]         934         832 - ~. 0.92    5' 0.48

                          ???               v           v      v    v                                    ???? NC
                    ~~~~~~______>>>,,,,,(((,,,<.<<<<________>>>>>,,<<<____>>>,<<<---<<<<~~~~~~>>>>---->>>,,,,)))]]]] CS
      Cobalamin   1 <[31]*agugaaggguuAAaaGGGAAc.ccGGUGaaAaUCCgggGCuGcCCCCgCaACuGUAAgcGg*[61]*cCgcgAGcCaGGAGACCuGCCa 174
                          +G+     + AA: GGAA: : GGUG AAAUCC ::+C:G CCC  C:ACUGUAA:C:         :G:+AG+CAG A AC :  C
 AAFY01022046.1 934 <[ 0]*GUAGGCAAAAGGAAGAGGAAGgAUGGUGGAAAUCCUUCACGGGCCCGGCCACUGUAACCAG*[ 4]*UUGGAAGUCAG-AUACUCUUCU 849
                    ......44455566666899******989**********************************97...7..79*********.9********* PP

                    ??               NC
                    ]]:::::::::::::: CS
      Cobalamin 175 ucaguuuuugaaucucc 191
                     ++++   GAA+CU C
 AAFY01022046.1 848 AUUAAGGCGGAAACUAC 832
                    ***************** PP
```

This alignment has some important differences with the ones we've seen so far because it is of a truncated hit. First, notice that the `trunc` column reads `5'` indicating that Infernal predicts the 5' end (beginning) of this Cobalamin riboswitch is missing. (Note that this hit is on the bottom (reverse) strand so the Cobalamin hit is actually predicted to extend past the end of the input sequence (past residue 934), but on the opposite strand.) The 5' end of the alignment indicates this with special annotation: the `<[31]*` in the model line indicates that the missing sequence is expected to align to the 31 5'-most positions of the Cobalamin model (i.e. about 31 residues are missing) and the first `a` residue in this line corresponds to model position 32; the `<[0]*` annotation in the sequence line indicates that there are no observed residues which align to those 31 positions and the first `G` residue is at position 934 of the sequence, which is the first position (on the opposite strand) of the query sequence. If, alternatively, this sequence was 3' truncated, or both 5' *and* 3' truncated, there would be analogous annotation at the 3' end of the alignment. Also notice the `~]` and `~.` symbols following the model and sequence coordinates, respectively. The `~` leftmost symbol in both of these pairs indicate that this hit is truncated at the 5' end. To make sense of this alignment display, it may help to look at the `cmalign` alignment of the same sequence to the same model on page 30, which shows all model and sequence positions.

Truncated hit alignments also contain different annotation in the NC lines. Instead of only containing blank spaces or v characters indicating negative scoring noncanonical basepairs, ? characters are used to denote basepairs for which the other half is missing due to the truncation. For example, the second g in the first model line corresponds to the right half of a basepair for which the left half is included in the stretch of 31 5' truncated model positions, so it is annotated with a ?. A ? is used because it is impossible to tell if such basepairs are negative scoring non-canonicals or not since we don't know the identity of the other half.

This hit alignment also demonstrates another type of annotation not yet seen in the previous examples, for *local end* alignments. Notice the stretch of six ~ characters towards the end of the first CS line, at the same position as the string `*[61]*` in the model line immediately below. This indicates a a special type of alignment called a local end. Local ends occur when a large insertion or deletion is used in the optimal alignment at reduced penalty (Klein and Eddy, 2003) and allow Infernal to be tolerant of the insertion and/or deletion of RNA substructures not modeled by the CM. An example of how local ends enable remote homology detection in RNase P can be see in Figure 6 on page 106. In this case, 61 model positions are deleted and 4 residues are inserted in the sequence (indicated by `*[ 4]*` at the corresponding positions in the sequence line). It is possible for zero sequence residues to be inserted by a local end, and for the number of residues inserted in a local end to exceed the number of model positions. Note that a single 7 annotates the posterior probability of the four sequence residues in the PP line. This means that the average posterior probability for these four residues is between 65 and 75%. If no sequence residues were in this EL, the PP annotation would be a gap (.) character.

Searching the Rfam CM database with a query sequence

The Rfam database http://rfam.xfam.org/ is a collection of RNA families, each represented by a CM and multiple sequence alignment used to build that CM. As of June 2016, the current release is 12.1, which includes 2474 families. The Rfam website allows web-based searches using cmscan of the Rfam CM database against query sequences that the user can upload. Alternatively, you can perform the same searches by running cmscan locally, as shown in this example. By searching all of Rfam with your sequence dataset, you will be annotating your dataset for most known types of structural RNAs with a single command.

To complete this step of the tutorial you'll need to download the Rfam 12.1 CM file from here: ftp://ftp.ebi.ac.uk/pub/databases/Rfam/12.1/Rfam.cm.gz and gunzipping it, like this:

```
> wget ftp://ftp.ebi.ac.uk/pub/databases/Rfam/12.1/Rfam.cm.gz
> gunzip Rfam.cm.gz
```

Then, as in the previous example, you'll need to run cmpress on this CM database:

```
> cmpress Rfam.cm
```

The next step is to run cmscan. In order to reproduce how Rfam searches are performed (Nawrocki et al., 2015) several command line options are required. Each of these options is explained below. The full command is (split up into two lines so it fits on the page):

```
> cmscan --rfam --cut_ga --nohmmonly --tblout mrum-genome.tblout --fmt 2 \
> --clanin testsuite/Rfam.12.1.clanin Rfam.cm tutorial/mrum-genome.fa > mrum-genome.cmscan
```

This command will take at least several minutes and possibly up to about 30 minutes depending on the number of cores and speed of your computer.

The command line options used in the above command are as follows:

--rfam Specifies that the filter pipeline run in fast mode, with the same strict filters that are used for Rfam searches and for other sequence databases larger than 20 Gb (see section 4).

--cut_ga Specifies that the special Rfam *GA* (gathering) thresholds be used to determine which hits are reported. These thresholds are stored in the Rfam.cm file. Each model has its own GA bit score threshold, which was determined by Rfam curators as the bit score at and above which all hits are believed to be true homologs to the model. These determinations were made based on observed hit results against the large Rfamseq database used by Rfam (Nawrocki et al., 2015).

`--nohmmonly` All models, even those with zero basepairs, are run in CM mode (not HMM mode). This ensures all GA cutoffs, which were determined in CM mode for each model, are valid.

`--tblout` Specifies that a tabular output file should be created, see section 6.

`--fmt 2` The tabular output file will be in format 2, which includes annotation of overlapping hits. See page 60 for a complete description of this format.

`--clanin` Clan information should be read from the file `testsuite/Rfam.12.1.claninfo`. This file lists which models belong to the same clan. Clans are groups of models that are homologous and therefore it is expected that some hits to these models will overlap. For example, the LSU_rRNA_archaea and LSU_rRNA_bacteria models are both in the same clan.

When the `cmscan` command finishes running, the file `mrum-genome.cmscan` will contain the standard output of the program. This file will be similar to what we saw in the earlier example of `cmscan`. The file `mrum-genome.tblout` has also been created, which is a tabular representation of all hits, one line per hit. Take a look at this file. The first two lines are comment lines (prefixed with `#` characters) with the labels of each of the 27 columns of data in the file. Each subsequent line has 27 space delimited tokens. The specific meaning of these tokens is described in detail in section 6. Below I'm including the first 24 lines of the file, with columns 3-5, 7-9 and 13-16 removed (replaced with `...`) so that the text will fit on this page:

```
#idx target name            ... clan name ... seq from   seq to strand ... score   E-value inc olp anyidx afrct1 afrct2 winidx wfrct1 wfrct2 description of target
#--- ---------------------- ... --------- ... -------- -------- ------ ... ----- --------- --- --- ------ ------ ------ ------ ------ ------ ---------------------
1    LSU_rRNA_archaea       ... CL00112   ...   762872   765862      + ... 2763.5         0   !   ^      -      -      -      -      -      - -
2    LSU_rRNA_archaea       ... CL00112   ...  2041329  2038338      - ... 2755.0         0   !   ^      -      -      -      -      -      - -
3    LSU_rRNA_bacteria      ... CL00112   ...   762874   765862      + ... 1872.9         0   !   =      1  1.000  0.999      "      "      " -
4    LSU_rRNA_bacteria      ... CL00112   ...  2041327  2038338      - ... 1865.5         0   !   =      2  1.000  0.999      "      "      " -
5    LSU_rRNA_eukarya       ... CL00112   ...   763018   765851      + ... 1581.3         0   !   =      1  1.000  0.948      "      "      " -
6    LSU_rRNA_eukarya       ... CL00112   ...  2041183  2038349      - ... 1572.1         0   !   =      2  1.000  0.948      "      "      " -
7    SSU_rRNA_archaea       ... CL00111   ...  2043361  2041888      - ... 1552.0         0   !   ^      -      -      -      -      -      - -
8    SSU_rRNA_archaea       ... CL00111   ...   760878   762351      + ... 1546.5         0   !   ^      -      -      -      -      -      - -
9    SSU_rRNA_bacteria      ... CL00111   ...  2043366  2041886      - ... 1161.9         0   !   =      7  0.995  1.000      "      "      " -
10   SSU_rRNA_bacteria      ... CL00111   ...   760873   762353      + ... 1156.4         0   !   =      8  0.995  1.000      "      "      " -
11   SSU_rRNA_eukarya       ... CL00111   ...  2043361  2041891      - ...  970.4 2.5e-289   !   =      7  1.000  0.998      "      "      " -
12   SSU_rRNA_eukarya       ... CL00111   ...   760878   762348      + ...  963.8 2.4e-287   !   =      8  1.000  0.998      "      "      " -
13   SSU_rRNA_microsporidia ... CL00111   ...  2043361  2041891      - ...  919.9 1.9e-277   !   =      7  1.000  0.998      "      "      " -
14   SSU_rRNA_microsporidia ... CL00111   ...   760878   762348      + ...  917.2 1.3e-276   !   =      8  1.000  0.998      "      "      " -
15   RNaseP_arch            ... -         ...  2614544  2614262      - ...  184.9   2.8e-50   !   *      -      -      -      -      -      - -
16   Archaea_SRP            ... CL00003   ...  1064321  1064634      + ...  197.6   1.7e-45   !   *      -      -      -      -      -      - -
17   FMN                    ... -         ...   193975   193837      - ...  115.2   1.7e-24   !   *      -      -      -      -      -      - -
18   tRNA                   ... CL00001   ...   735136   735208      + ...   72.1   1.2e-12   !   *      -      -      -      -      -      - -
19   tRNA                   ... CL00001   ...  2350593  2350520      - ...   71.0   2.5e-12   !   *      -      -      -      -      -      - -
20   tRNA                   ... CL00001   ...  2680310  2680384      + ...   70.9   2.6e-12   !   *      -      -      -      -      -      - -
21   tRNA                   ... CL00001   ...  2351254  2351181      - ...   69.7   5.5e-12   !   *      -      -      -      -      -      - -
22   tRNA                   ... CL00001   ...   361676   361604      - ...   69.5   6.2e-12   !   *      -      -      -      -      -      - -
```

This tabular format includes the target model name, sequence name (in column 3, which is omitted above to save space), clan name, sequence coordinates, bit score, E-value and more. Because the `--fmt 2` option was used, this file includes information on which hits overlap with other hits, starting at the column labelled "olp" and ending with "wfrct2". Hits with the "*" character in the "olp" column do not overlap with any other hits. Those with "^" do overlap with at least one other hit, but none of those overlapping hits have a better score (that occurs higher in the list). Those with "=" also overlap with at least one other hit that does have a better score, the index of which is given in the "anyidx" column. For more detailed explanation of these columns, see page 60.

The top two hits are both to the `LSU_rRNA_archaea` model. These are the two copies of LSU rRNA in the *Methanobrevibacter ruminantium* genome. Hits number 3 and 4 are to the `LSU_rRNA_bacteria` model and overlap with hits 1 and 2 nearly completely (hit 1 is from sequence positions 762872 to 765862 and hit 3 is from sequence positions 762874 to 765862). This overlap is not surprising because the bacterial and archaeal LSU rRNA models are very similar, and so are assigning high scores to the same subsequences. Further, hit 5 is to `LSU_rRNA_eukarya` and also overlaps hits 1 and 3. Because these three LSU models are all expected to produce overlapping hits due to their homology, Rfam has grouped them into the same *clan*, note the "CL00112" value in the "clan name" column for all three hits. This clan information was provided in the `rfam.12.1claninfo` input file we provided to `cmscan` by using the `--clanin` option.

The "olp" column indicates that hit 1 is the highest scoring of the three overlapping hits because it contains the "^" character. Hits 3 and 5 both have "=" in the "olp" column indicating that there is another hit to another model which overlaps these hits and has a better score.

If you were using these results to produce annotations for the *Methanobrevibacter ruminantium* genome, you may want to ignore any hits that have higher scoring overlaps. To do this you can just remove any hits with "=" in the "olp" column. Alternatively, you can have these hits not printed to the tabular output file by additionally providing the `--oskip` option to `cmscan`. You can also modify the overlap annotation behavior with `--oclan` option which restricts the annotation of overlaps to hits for models within the same clan. Overlapping hits from models that are not in the same clan will not be marked as overlaps, instead they will marked as "*" in the "olp" field.

Creating multiple alignments with cmalign

The file `tutorial/mrum-tRNAs10.fa` is a FASTA file containing the 10 tRNA hits above the inclusion threshold (with an E-value less than 0.01) found by `cmsearch` in our search of *M. ruminantium* genome[8] To align these 10 sequences to our example tRNA model and make a multiple sequence alignment:

`> cmalign tRNA5.cm tutorial/mrum-tRNAs10.fa`

The output of this is a Stockholm format multiple alignment file:

```
# STOCKHOLM 1.0
#=GF AU Infernal 1.1.2

mrum-tRNA.1          GGAGCUAUAGCUCAAU..GGC..AGAGCGUUUGGCUGACAU........................................CCAAAAGGUUAUGGGUUCGAUUCCCUUUAGCCCCA
#=GR mrum-tRNA.1  PP ****************..***..******************........................................***********************************
mrum-tRNA.2          GGGCCCGUAGCUCAGU.uGGG..AGAGCGCUGCCCUUGCAA........................................GGCAGAGGCCCCGGGUUCAAAUCCCGGUGGGUCCA
#=GR mrum-tRNA.2  PP ****************.****..******************........................................***********************************
mrum-tRNA.3          GGGCCCAUAGCUUAGCcaGGU..AGAGCGCUCGGCUCAUAA........................................CCGGGAUGUCAUGGGUUCGAAUCCCAUUGGGCCCA
#=GR mrum-tRNA.3  PP *********************..******************........................................***********************************
mrum-tRNA.4          AGGCUAGUGGCACAGCcuGGU.cAGCGCGCACGGCUGAUAA........................................CCGUGAGGUCCUGGGUUCGAAUCCCAGCUAGCCUA
#=GR mrum-tRNA.4  PP ***************999***.*******************........................................***********************************
mrum-tRNA.5          CCCGACUUAGCUCAAUuuGGC..AGAGCGUUGGACUGUAGA........................................UCCAAAUGUUGCUGGUUCAAGUCCGGCAGUCGGGA
#=GR mrum-tRNA.5  PP *********************..******************........................................***********************************
mrum-tRNA.6          GCUUCUAUGGGGUAAU.cGGC.aAACCCAUCGGACUUUCGA........................................UCCGAUAA-UCCGGGUUCAAAUCCCGGUAGAAGCA
#=GR mrum-tRNA.6  PP ****************.****.*******************........................................********.9*************************
mrum-tRNA.7          GCUCCGAUGGUGUAGUccGGCcaAUCAUUUCGGCCUUUCGA........................................GCCGAAGA-CUCGGGUUCGAAUCCCGGUCGGAGCA
#=GR mrum-tRNA.7  PP *******************9999******************........................................********.**************************
mrum-tRNA.8          GCGGUGUUAGUCCAGCcuGGU.uAAGACUCUAGCCUGCCAC........................................GUUAGAGA-CCCGGGUUCAAAUCCCGGACGCCGCA
#=GR mrum-tRNA.8  PP ***************999***.*******************........................................********.**************************
mrum-tRNA.9          GCCGGGGUGGCUCAGC.uGGU.uAGAGCGCACGGCUC----auaggguaacuaagcgugcucugacuuuuuuccugggauaCCGUGAGAUCGCGGGUUCGAAUCCCGCCCCCGGCA
#=GR mrum-tRNA.9  PP ****************.****.************995....678*************************************************************************
mrum-tRNA.10         GGUUCUAUAGUUUAAC.aGGU..AAAACAACUGGCUGUUAA........................................CCGGCAGA-UAGGAGUUCGAAUCUUCUUAGAACCG
#=GR mrum-tRNA.10 PP ****************.****..******************........................................********.9*************************
#=GC SS_cons         (((((((,,<<<<___..___.._>>>>,<<<<<_______~~~~~~~~~~~~~~~~~~~~~~~~~~~~~~~~~~~~~~~~>>>>>,,,,,<<<<<_______>>>>>))))))):
#=GC RF              gCcggcAUAGcgcAgU..GGu..AgcgCgccagccUgucAa~~~~~~~~~~~~~~~~~~~~~~~~~~~~~~~~~~~~~~~~gcuggAGgUCCgggGUUCGAUUCcccGUgccgGca
//
```

The first thing to notice here is that `cmalign` uses both lower case and upper case residues, and it uses two different characters for gaps. This is because there are two different kinds of columns: "match" columns in which residues are assigned to match states and gaps are treated as deletions relative to consensus, and "insert" columns where residues are assigned to insert states. In a match column, residues are upper case, and a '`-`' character means a deletion relative to the consensus. In an insert column, residues are lower case, and a '`.`' is padding. A '`-`' deletion has a cost: transition probabilities were assessed, penalizing the transition into and out of a deletion. A '`.`' pad has no cost per se; instead, the sequence(s) with insertions are paying transition probabilities into and out of their inserted residue.

Actually, there's two types of insert columns: standard insert columns where residues are assigned to insert states and less common "local end" insertion columns where residues are assigned to a special local end state called the "EL" state. There is one EL state per model and it allows a CM to permit large insertions or deletions in the structure present in the alignment the model was built from, at a reduced cost. This can help Infernal detect remote homologs in some cases. We saw an example of this in our Cobalamin `cmscan` hit above. Another example is shown in Figure 6 on page 106. Columns containing EL insertions are denoted in the `#=GC RF` annotation, described next.

Take a look at the final two lines of the alignment. The `#=GC RF` line is Stockholm-format *reference coordinate annotation*, with a residue marking each column that the CM considered to be consensus, a '`.`' marking insert columns and a '`~`' marking EL insert columns. For match columns, upper case residues denote strongly conserved columns, and lower case denotes weakly conserved ones. The `#=GC SS_cons` line gives the consensus secondary structure of the model. The symbols here have the same meaning that they did in a pairwise alignment from `cmsearch`, for a detailed description see section 9. As in the RF annotation, EL columns are indicated by '`~`'. In this alignment, `mrum-tRNA.9` is the only sequence that uses the EL state.

Important: both standard and local end insertions in a CM are *unaligned* (the same way that insertions are unaligned in profile HMM alignments produced by the HMMER package). Suppose one sequence has an insertion of length 10 and another has an insertion of length 2 in the same place in the model. The alignment will show ten insert columns, to accomodate the longest insertion. The residues of the shorter insertion are thrown down in an arbitrary order. (If you must know: by arbitrary Infernal convention, the insertion is divided in half; half is left-justified, and the other half is right-justified, leaving '.' characters in the middle.) Notice that in the previous paragraph I oh-so-carefully said residues are "assigned" to a state, not "aligned". For match states, assigned and aligned are the same thing: a one-to-one correspondence between a residue and a consensus match state in the model. But there may be one *or more* residues assigned to the same insert state.

Don't be confused by the unaligned nature of CM insertions. You're sure to see cases where lower-case inserted residues are "obviously misaligned". This is just because Infernal isn't trying to "align" them in the first place: it is assigning them to unaligned insertions. For example of an obvious misalignment look at sequences `mrum-tRNA.6`

[8] The `-A <f>` option to `cmsearch` can be used to save a Stockholm formatted multiple alignment of all hits above the inclusion threshold to file `<f>`.

and `mrum-tRNA.8` in the above example alignment. The first inserted (lowercase) `c` in `mrum-tRNA.6` would be better aligned with respect to `mrum-tRNA.8` if it were placed one position to the left.

Enough about the sequences in the alignment. Now notice all those `PP` annotation lines. That's posterior probability annotation, as in the single sequence alignments that `cmscan` and `cmsearch` showed. This essentially represents the confidence that each residue is aligned where it should be.

Er, I mean, "assigned", not "aligned". The posterior probability assigned to an inserted residue is the probability that it is assigned to the insert state that corresponds to that column, or the EL state in the case of local end insertions. Because the same insert state might correspond to more than one column, the probability on an insert residue is *not* the probability that it belongs in that particular column; again, where there's a choice of column for inserted residues, that choice is arbitrary.

cmalign assumes sequences may be truncated

Unlike in all previous versions of Infernal, `cmalign` in version 1.1 assumes that input sequences may be truncated and uses specialized dynamic programming algorithms to align them appropriately based on that assumption[9]. Importantly, output alignments from `cmalign` do not indicate when sequences have been truncated, as those from `cmsearch` and `cmscan` do. As an example, use the `tutorial/Cobalamin.c.cm` model to align the truncated Cobalamin hit (in the file `tutorial/Cobalamin.fa`) from the `cmscan` example above (I've split up the alignment below so it will fit on the page):

```
> cmalign tutorial/Cobalamin.c.cm tutorial/Cobalamin.fa

# STOCKHOLM 1.0
#=GF AU Infernal 1.1.2

Cobalamin.1         -------------------------------GUAGGCAAAAGGAAGAGGAAGgAUGGUGGAAAUCCUUCACGGGCCCGGCCA
#=GR Cobalamin.1 PP ...............................44455566666899******989****************************
#=GC SS_cons        :::::::::::::::[[[[[[,<<<____________>>>,,,,,(((,,,<.<<<<_______>>>>>,,<<<____>>>,
#=GC RF             uuaaauugaaacgaugauGGUuccccuuuaaagugaaggguuAAaaGGGAAc.ccGGUGaaAaUCCgggGCuGcCCCCgCaA

Cobalamin.1         CUGUAACCAG---------------------------------auuu----------------------------UUGGAAG
#=GR Cobalamin.1 PP ********97.................................5689............................79*****
#=GC SS_cons        <<<---<<<<------<<<<<<-----<<<-<<<<<<______~~~~>>>>>>--->>>>>>>>>---------->>>>---
#=GC RF             CuGUAAgcGgagagcaccccccAauaaGCCACUggcccgcaag~~~~ggccGGGAAGGCggggggaaggaaugaccCgcgAG

Cobalamin.1         UCAG-AUACUCUUCUAUUAAGGCGGAAACUAC
#=GR Cobalamin.1 PP ****.9*************************
#=GC SS_cons        ->>>,,,,)))]]]]]]::::::::::::::
#=GC RF             cCaGGAGACCuGCCaucaguuuuugaaucucc
//
```

If you look back to the `cmscan` hit alignment for this sequence, you'll notice that this looks a little different. Instead of the `<[31]*` annotation in the model line indicating 31 model positions were missing due to a presumed 5' truncation, `cmalign` includes those 31 positions, and their secondary structure annotation. The local end alignment annotation in the middle of the second line is different too. In the `cmscan` hit alignment the model line included `*[61]*` indicating that 61 model positions were skipped to a local end insertion. In the `cmalign` output those 61 positions are included, aligned to gaps in the sequence. `cmalign` also shows the identity of the four inserted residues in the EL state, and annotates each with a posterior probability, whereas `cmscan` only indicated the number of inserted EL residues and their average posterior probability.

If you examine the `cmscan` and `cmalign` alignments closely you'll notice that they are identical (the same sequence residues are aligned to the same model positions in each) and include identical posterior probability annotation. While this will be the case for the large majority of sequences, it is not guaranteed by Infernal, and sometimes a `cmsearch` or `cmscan` alignment of a hit will differ from a `cmalign` alignment of the same hit. There are several technical reasons for this, including the fact that the HMM band constraints used by `cmalign` can differ from those used by `cmsearch` and `cmscan` which in rare cases can lead to a different alignment or different posterior probabilities. Another reason is that while `cmalign` always assumes a sequence may be 5' or 3' truncated, `cmsearch` and `cmscan` only allow certain types of truncation (5', 3' or both) at sequence terminii. The types of truncated alignment allowed modify the dynamic programming alignment algorithm, so this difference can also result in different alignments. However, most of the time the alignments will be identical, and when they are different they will usually only be slightly different.

---

[9] In versions 1.0 through 1.0.2, the `cmalign --sub` option was recommended when input sequences may have been truncated. This option still exists in this version but we believe the new default mode should do a better job of correctly aligning truncated sequences.

Searching a sequence database for RNAs with unknown or no secondary structure

Some functional RNAs, including many types of small nucleolar RNAs (snoRNAs) do not conserve a secondary structure. It is more appropriate to model such RNA families with a profile HMM than a CM, because CMs only differ from profile HMMs in their modeling of basepairs and because profile HMM scoring algorithms are more efficient than CM ones.

Likewise, profile HMMs are more appropriate than CMs for modeling RNAs of unknown structure. For such RNAs, a profile HMM search may reveal additional homologs that can help elucidate the conserved structure for the family, if there is one. Given the multiple homologs, you can use other programs like RNAalifold (Bernhart et al., 2008; Hofacker et al., 2002) or WAR (Torarinsson and Lindgreen, 2008) (a webserver that includes implementations of several programs) to try to predict the conserved secondary structure of a collection of homologous RNAs. Currently, Infernal itself does not have the capability of predicting structure, but it's predecessor COVE did with the `covet` program, still available at `eddylab.org/software/cove/cove.tar.Z`.

Infernal automatically detects when a model has zero basepairs and uses efficient profile HMM algorithms in `cmsearch` and `cmscan`. As an example, let's revisit the construction of our tRNA model but pretend that we do not know the conserved cloverlear structure of tRNA. The file `tutorial/tRNA5-noss.sto` is identical to the file `tutorial/tRNA5.sto` except that it does not contain consensus secondary structure annotation. To build a structureless tRNA model from this alignment, execute:

`> cmbuild --noss tRNA5-noss.cm tutorial/tRNA5.sto`

The `--noss` option is required when the alignment file lacks structure annotation. By forcing the use of `--noss` we're forcing the user to realize that having no secondary structure is a special case for Infernal.

The output will be similar to be the earlier example, but not identical:

```
# cmbuild :: covariance model construction from multiple sequence alignments
# INFERNAL 1.1.2 (June 2016)
# Copyright (C) 2016 Howard Hughes Medical Institute.
# Freely distributed under a BSD open source license.
# - - - - - - - - - - - - - - - - - - - - - - - - - - - - - - - - - - - -
# CM file:                                          tRNA5-noss.cm
# alignment file:                                   tutorial/tRNA5.sto
# ignore secondary structure, if any:               yes
# - - - - - - - - - - - - - - - - - - - - - - - - - - - - - - - - - - - -
#                                                                rel entropy
#                                                                -----------
# idx    name                      nseq eff_nseq   alen  clen  bps bifs    CM   HMM description
# ------ -------------------- -------- -------- ------ ----- ---- ---- ----- ----- -----------
       1 tRNA5                        5     5.00     74    72    0    0 0.552 0.552
#
# CPU time: 0.14u 0.00s 00:00:00.14 Elapsed: 00:00:00.14
```

The output reports that this model has 0 basepairs ("bps") (the earlier example had 21) and that the CM relative entropy is different from before because basepairs have been ignored. Now the CM and HMM relative entropy are identical, because the CM can be mirrored nearly identically by a profile HMM.

Remember that after we built our tRNA CM with structure above, we needed to use `cmcalibrate` to calibrate the model before we could perform searches. Importantly, zero basepair models do not need to be calibrated prior to running `cmsearch`[10], so we can skip that step here.

Now, let's repeat the search of the *M. ruminantium* genome but using our structureless tRNA model:

`> cmsearch tRNA5-noss.cm tutorial/mrum-genome.fa`

This search is faster than the first one because only profile HMM algorithms are used this time around. Take a look at the list of hits:

[10] Zero basepair models do need to be calibrated before they can be used with `cmscan` however.

```
rank     E-value  score  bias  sequence      start     end   mdl trunc   gc  description
----   --------- ------ -----  ----------- ------- -------   --- ----- ----  -----------
 (1) !   3.6e-09   38.2   0.0  NC_013790.1  362022  361960 - hmm     - 0.46  Methanobrevibacter ruminantium M1 chromosome
 (2) !     3e-07   32.2   0.0  NC_013790.1  762496  762559 + hmm     - 0.64  Methanobrevibacter ruminantium M1 chromosome
 (3) !     3e-07   32.2   0.0  NC_013790.1 2041698 2041635 - hmm     - 0.64  Methanobrevibacter ruminantium M1 chromosome
 (4) !     5e-06   28.3   0.0  NC_013790.1 2585264 2585203 - hmm     - 0.56  Methanobrevibacter ruminantium M1 chromosome
 (5) !   6.1e-06   28.1   0.0  NC_013790.1  735143  735198 + hmm     - 0.59  Methanobrevibacter ruminantium M1 chromosome
 (6) !     7e-06   27.9   0.0  NC_013790.1 2351247 2351188 - hmm     - 0.57  Methanobrevibacter ruminantium M1 chromosome
 (7) !   1.2e-05   27.2   0.0  NC_013790.1  662193  662251 + hmm     - 0.64  Methanobrevibacter ruminantium M1 chromosome
 (8) !   1.2e-05   27.1   0.0  NC_013790.1 2186009 2185951 - hmm     - 0.51  Methanobrevibacter ruminantium M1 chromosome
 (9) !   4.2e-05   25.4   0.0  NC_013790.1 2585183 2585118 - hmm     - 0.56  Methanobrevibacter ruminantium M1 chromosome
(10) !   6.4e-05   24.8   0.0  NC_013790.1 1873882 1873820 - hmm     - 0.63  Methanobrevibacter ruminantium M1 chromosome
(11) !   0.00014   23.7   0.0  NC_013790.1  360882  360824 - hmm     - 0.51  Methanobrevibacter ruminantium M1 chromosome
(12) !   0.00059   21.8   0.0  NC_013790.1  361910  361851 - hmm     - 0.38  Methanobrevibacter ruminantium M1 chromosome
(13) !   0.00092   21.2   0.0  NC_013790.1 2350586 2350528 - hmm     - 0.58  Methanobrevibacter ruminantium M1 chromosome
(14) !    0.0018   20.3   0.0  NC_013790.1  995341  995267 - hmm     - 0.51  Methanobrevibacter ruminantium M1 chromosome
(15) !    0.0026   19.7   0.0  NC_013790.1   97728   97788 + hmm     - 0.49  Methanobrevibacter ruminantium M1 chromosome
(16) !    0.0029   19.6   0.0  NC_013790.1 2186083 2186024 - hmm     - 0.50  Methanobrevibacter ruminantium M1 chromosome
(17) !    0.0031   19.5   0.0  NC_013790.1 2130421 2130351 - hmm     - 0.59  Methanobrevibacter ruminantium M1 chromosome
(18) !    0.0044   19.0   0.0  NC_013790.1  360727  360670 - hmm     - 0.43  Methanobrevibacter ruminantium M1 chromosome
(19) !    0.0057   18.7   0.0  NC_013790.1 1160527 1160608 + hmm     - 0.59  Methanobrevibacter ruminantium M1 chromosome
(20) !    0.0074   18.3   0.0  NC_013790.1  361056  360994 - hmm     - 0.40  Methanobrevibacter ruminantium M1 chromosome
------ inclusion threshold ------
(21) ?     0.011   17.7   0.0  NC_013790.1 2151679 2151737 + hmm     - 0.56  Methanobrevibacter ruminantium M1 chromosome
(22) ?     0.018   17.1   0.0  NC_013790.1 2327123 2327043 - hmm     - 0.62  Methanobrevibacter ruminantium M1 chromosome
(23) ?     0.023   16.7   0.0  NC_013790.1  360973  360920 - hmm     - 0.54  Methanobrevibacter ruminantium M1 chromosome
(24) ?     0.037   16.1   0.0  NC_013790.1 2350982 2350919 - hmm     - 0.50  Methanobrevibacter ruminantium M1 chromosome
(25) ?     0.039   16.1   0.0  NC_013790.1  361671  361606 - hmm     - 0.50  Methanobrevibacter ruminantium M1 chromosome
(26) ?     0.039   16.0   0.0  NC_013790.1 2680176 2680227 + hmm     - 0.62  Methanobrevibacter ruminantium M1 chromosome
(27) ?     0.062   15.4   0.0  NC_013790.1 2585067 2585000 - hmm     - 0.59  Methanobrevibacter ruminantium M1 chromosome
(28) ?     0.063   15.4   0.0  NC_013790.1  362793  362738 - hmm     - 0.46  Methanobrevibacter ruminantium M1 chromosome
(29) ?     0.063   15.4   0.0  NC_013790.1 1064778 1064857 + hmm     - 0.62  Methanobrevibacter ruminantium M1 chromosome
(30) ?     0.072   15.2   0.0  NC_013790.1 2749938 2749884 - hmm     - 0.47  Methanobrevibacter ruminantium M1 chromosome
(31) ?     0.073   15.2   0.0  NC_013790.1 2749832 2749778 - hmm     - 0.47  Methanobrevibacter ruminantium M1 chromosome
(32) ?      0.12   14.5   0.0  NC_013790.1  361729  361689 - hmm     - 0.56  Methanobrevibacter ruminantium M1 chromosome
(33) ?       1.3   11.2   0.0  NC_013790.1  361439  361393 - hmm     - 0.49  Methanobrevibacter ruminantium M1 chromosome
(34) ?       4.4    9.6   0.0  NC_013790.1 2583867 2583803 - hmm     - 0.49  Methanobrevibacter ruminantium M1 chromosome
(35) ?       5.9    9.2   0.0  NC_013790.1  546054  546021 - hmm     - 0.68  Methanobrevibacter ruminantium M1 chromosome
```

Note that this time we only find 35 hits (20 with E-values less than the inclusion threshold of 0.01) compared to 56 (with 54 less than 0.01) in the original search with the structure-based CM. The increased sensitivity in the initial CM search exemplifies the additional power that comes with knowledge of the conserved secondary structure. Not all RNAs will show such a dramatic difference though. In fact, tRNA is an particular strong example of the power of CMs versus sequence-only based methods like HMMs because about as much statistical signal is present in their structure as in their primary sequence. Many structural RNAs contain significantly less information in their structure than in sequence conservation, but many include 10 bits or more of information in their structure. An additional 10 bits roughly translates into lowering the expected statistical significance of homologs detected in database searches by about 3 orders or magnitude[11].

HMM hit alignments differ slightly from CM hit alignments. Take a look at the first alignment:

```
>> NC_013790.1  Methanobrevibacter ruminantium M1 chromosome, complete genome
 rank     E-value  score  bias mdl mdl from   mdl to       seq from      seq to       acc trunc   gc
 ----   --------- ------ ----- --- -------- --------    ----------- -----------      ---- ----- ----
  (1) !   3.6e-09   38.2   0.0 hmm        5       67 ..      362022      361960 - .. 0.93     - 0.46

                      ::::::::::::::::::::::::::::::::::::::::::::::::::::::::::::::: CS
       tRNA5        5 auAUaGcgcAGUGGuAGcGCGgcagcCUgucaagguggAGGUCCuggGUUCGAUUCccaGUgu 67
                       uAUaGc+cA+UGG AG+GCG  +g CUg ca  +   AGGU  uggGUUCGAUUCcc  U+
  NC_013790.1 362022 CUAUAGCUCAAUGGCAGAGCGUUUGGCUGACAUCCAAAAGGUUAUGGGUUCGAUUCCCUUUAG 361960
                     689******************************************************988876 PP
```

First, note that the `mdl` field reads `hmm` instead of `cm`. Also, because HMMs do not model basepairs, there is no `NC` annotation pointing out negative scoring noncanonical basepairs.

If you were to look at more hits you may notice that HMM hits are more likely to not be full length than CM hits are. This hit, for example, begins at model position 5 and ends at model position 67, whereas our earlier CM search included a full length alignment from model position 1 to 72 for the same region of the genome. The profile HMMs used in Infernal allow local alignments that begin and end at any position in the model, with an equal score given for all possible start and end positions (this is the same local alignment strategy used in HMMER 3.0 (Eddy, 2008)). In

[11]For a graph showing the relative amount of information in structure for sequence for many different types of RNAs see Figure 1.9 of (Nawrocki et al., 2009)

contrast, the CM local alignment strategy used by Infernal encourages global alignments by enforcing a score penalty for local ones. Partly because CM and HMM alignments differ in this way, "truncated" HMM hits can start and end anywhere in the target sequence (instead of being identified only with specialized algorithms only at sequence ends like a CM), and so the `trunc` field is invalid for HMM hits and will always read "-".

In `cmscan`, HMM algorithms will be used to compute alignments for all models in the CM database that contain zero basepairs, and CM algorithms will be used for all others. Importantly though, unlike with `cmsearch` even models with zero basepairs must be calibrated prior to using them with `cmscan`. Hits found with the HMM will be annotated like this example above, while CM hits will be annotated like the earlier CM examples.

By default, Infernal uses HMM algorithms for models with zero basepairs, mainly because they are more efficient. If you'd like, you can force the use of CM algorithms for such models with the `--nohmmonly` option with `cmsearch` or `cmscan`. Using `--nohmmonly` will encourage more full length hits, but will cause the program to run a few-fold slower, and also requires that the CM be calibrated with `cmcalibrate` first.

Forcing global CM alignment with the -g option

By default, `cmsearch`, `cmscan` and `cmalign` allow local *or* global alignments between the sequence and the model. This allows an alignment to use what's called a "local begin" and start at any match position in the model and to use what's called a "local end" to prematurely exit a section of the model to handle large insertions or deletions of the model structure and potentially insert nonhomologous residues into the alignment (these were the ~ annotated residues in the `cmscan` and `cmalign` examples above). Local alignments are permitted by default, because our internal benchmarks suggest this strategy is more sensitive for remote homology detection. (An example of a local RNase P alignment is demonstrated in Figure 6 on page 106 of this guide.) However, some users may wish to turn off local alignment. This can be done using the `-g` option to `cmsearch`, `cmscan`, and `cmalign`. The use of local mode by default in `cmalign` is new to version 1.1 - all previous versions used global alignment by default. Importantly, using `-g` does not turn off truncated hit detection and alignment; to do that use `--notrunc`.

Specifying and annotating match positions with cmbuild –hand

When `cmbuild` constructs a model from an input alignment it must decide which columns of the input alignment to define as match (also called "consensus") and insert columns. To do this, it firsts computes weights for each sequences using an *ad hoc* algorithm to downweight closely related sequences and upweight distantly related ones. Then, for each position, the sum of the weights of the sequences that include a residue at the position is computed. If this sum is at least half the total number of sequences then the position is defined as a match position, else it is an insert position. If you're curious which positions get annotated as match versus insert in your alignment, use the `-O <f>` option with `cmbuild`, which will save a annotated version of your input alignment to file `<f>`, including the weights of each sequence. In this output alignment, positions with residues in the `#=GC RF` lines of the alignment are match positions, and positions with gap characters are inserts.

Sometimes you may want to override this automated behavior by specifying which columns be defined as match/insert, especially if you've painstakingly created your alignment by hand. You can do this with the `--hand` option[12]. Using this option you can also define a character to annotate each match position, which will be propagated to alignment output. In this section we'll go through an example of using `--hand` with the 5 sequence tRNA alignment from earlier in the tutorial.

Take a look at the file `tutorial/tRNA5-hand.sto`:

[12]This is the same option as `--rf` from previous versions of Infernal.

```
# STOCKHOLM 1.0

tRNA1             GCGGAUUUAGCUCAGUUGGG.AGAGCGCCAGACUGAAGAUCUGGAGGUCC
tRNA2             UCCGAUAUAGUGUAAC.GGCUAUCACAUCACGCUUUCACCGUGGAGA.CC
tRNA3             UCCGUGAUAGUUUAAU.GGUCAGAAUGGGCGCUUGUCGCGUGCCAGA.UC
tRNA4             GCUCGUAUGGCGCAGU.GGU.AGCGCAGCAGAUUGCAAAUCUGUUGGUCC
otRNA5             GGGCACAUGGCGCAGUUGGU.AGCGCGCUUCCCUUGCAAGGAAGAGGUCA
#=GC SS_cons      <<<<<<<..<<<<.........>>>>.<<<<<.......>>>>>.....<
#=GC RF           [accep]======[=Dloop=]============acd=======[vlp]=

tRNA1             UGUGUUCGAUCCACAGAAUUCGCA
tRNA2             GGGGUUCGACUCCCCGUAUCGGAG
tRNA3             GGGGUUCAAUUCCCCGUCGCGGAG
tRNA4             UUAGUUCGAUCCUGAGUGCGAGCU
tRNA5             UCGGUUCGAUUCCGGUUGCGUCCA
#=GC SS_cons      <<<<.......>>>>>>>>>>>>.
#=GC RF           ====[Tloop]=====[accep]=
//
```

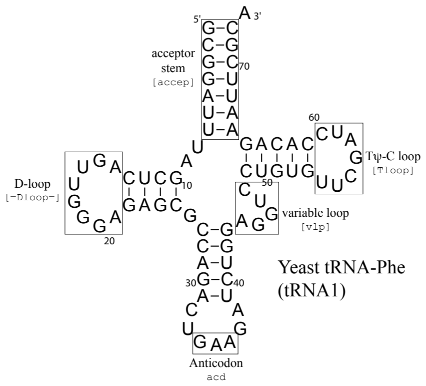

This file is the same as `tutorial/tRNA5.sto` except for the two additional lines beginning with `#=GC RF`. This RF (reference) annotation is required for using `--hand`. When `--hand` is used, any non-gap character in the reference annotation will be assigned as a match (consensus) position. Importantly, four different characters are considered gaps: dashes (`-`), underscores (`_`), dots (`.`) and tildes (`~`). In this example alignment, all columns are non-gap characters, so all columns will be considered match positions.

Different regions of the secondary structure have been marked up using abbreviations for the names of the regions in the reference annotation. For example, `acd` annotates the three positions of the anticodon, and `[vlp]` annotates the so-called variable loop. I've used `[` and `]` to indicate region boundaries in some cases. Crucially, I've avoided the use of any gap characters for positions between named regions which I still want to be considered match positions, and opted to use `=` (which is not considered a gap by `cmbuild`) for these positions.

To build the hand-specified model from this alignment, do:

`> cmbuild --hand tRNA5-hand.cm tutorial/tRNA5-hand.sto`

```
# cmbuild :: covariance model construction from multiple sequence alignments
# INFERNAL 1.1.2 (June 2016)
# Copyright (C) 2016 Howard Hughes Medical Institute.
# Freely distributed under a BSD open source license.
# - - - - - - - - - - - - - - - - - - - - - - - - - - - - - - - - - - - -
# CM file:                                           tRNA5-hand.cm
# alignment file:                                    ../../tutorial/tRNA5-hand.sto
# use #=GC RF annotation to define consensus columns: yes
# - - - - - - - - - - - - - - - - - - - - - - - - - - - - - - - - - - - -
#                                                                  rel entropy
#                                                                  -----------
# idx    name                     nseq eff_nseq   alen  clen  bps bifs    CM   HMM description
# ------ -------------------- -------- -------- ------ ----- ---- ---- ----- ----- -----------
       1 tRNA5-hand                  5     3.59     74    74   21    2 0.763 0.476
#
# CPU time: 0.31u 0.00s 00:00:00.31 Elapsed: 00:00:00.32
```

The output reports that the model now has 74 match (consensus) positions in the `clen` column. If we had built this model without specifying `--hand` (as we did earlier in this tutorial) the resulting model would have had only 72 consensus positions. (I've annotated the two extra match positions with three gaps in `tRNA5.hand.sto` as match solely to demonstrate how `--hand` works, not because I think it's better to model these positions as matches than inserts.)

Now, let's use this model to search the *M. ruminantium* genome again. First, the model must be calibrated. To save time, a calibrated version of the file is in `tutorial/tRNA5-hand.c.cm`. To do the search:

`> cmsearch tutorial/tRNA5-hand.c.cm tutorial/mrum-genome.fa`

The results are very similar to the earlier search with the tRNA model built with default `cmbuild` parameters (though not identical since the model now has two additional match positions). The important difference involves the hit alignments. Take a look at the alignment for hit number 46 as an illustrative example:

```
>> NC_013790.1  Methanobrevibacter ruminantium M1 chromosome, complete genome
 rank     E-value  score  bias mdl mdl from   mdl to       seq from      seq to       acc trunc   gc
 ----   --------- ------ ----- --- -------- --------    ----------- -----------      ---- ----- ----
 (46) !   1.6e-09   43.2   0.0  cm        1       74 []      995344      995263 - .. 0.90    no 0.49

                                 v          v                                                           NC
                     (((((((,,<<<<________._>>>>,<<<<<_______>>>>>,,,.......,,<<<<<_______>>>>>))))))): CS
   tRNA5-hand      1 gCcggcaUAGcgcAgUUGGuu.AgcgCgccagccUgucAagcuggAGg.......UCCgggGUUCGAUUCcccGugccgGca 74
                     :::G:CAUAGCG AG  GGU+ A CGCG:CAG:CU +++A:CUG: G+        UC:GGGGUUCGA UCCCC:UG:C:::A
```

```
NC_013790.1 995344 AGAGACAUAGCGAAGC-GGUCaAACGCGGCAGACUCAAGAUCUGUUGAuuaguucuUCAGGGGUUCGAAUCCCCUUGUCUCUA 995263
                   ************9***.8886258***********************9444444445************************** PP
                   [accep]======[=Dloop=.]============acd=======[vl........p]=====[Tloop]=====[accep]= RF
```

The reference annotation from the training alignment to `cmbuild` has been propagated to the hit as an extra `RF` line at the bottom of the alignment. All inserts in the alignment are annotated as `.` columns in the RF annotation. Note that the variable loop (annotated as `[vlp]` in the training alignment) contains 8 inserted residues. The RF annotation will also be transferred to multiple alignments created with `cmalign`.

4 Infernal 1.1's profile/sequence comparison pipeline

In this section, we briefly outline the processing pipeline for a single profile/sequence comparison. This should help give you a sense of what Infernal is doing under the hood, what sort of mistakes it may make, and what the various results in the output actually mean. If you haven't already worked through the tutorial section of the guide, you should do that first before reading this section as it lays some foundation for this discussion.

We'll first discuss the *standard* pipeline used by Infernal, which is excecuted on each comparison between a CM and full length sequence. Then we'll discuss *truncated variants* of the pipeline that are *re*run on the sequence ends for detection of truncated hits. Finally, we'll cover the HMM-only pipeline which is run for models with zero basepairs. Before we begin, a few notes on terminology. In this discussion, the term "profile" refers to either a profile HMM filter or a CM, and nucleotide and residue are used interchangeably for a single symbol of an input DNA/RNA sequence (even though "residue" traditionally refers to an amino acid residue of a protein sequence). Also, if I refer to "the pipeline" without specifying which variant (standard or a truncated one), then I mean the standard one.

Infernal's standard pipeline is based closely on the profile HMM/sequence comparison pipeline in HMMER3 (`hmmer.org`). In fact, the first several stages of the pipeline use code from HMMER's pipeline to score candidate sequences with profile HMMs that act as filters for the later, more computationally expensive CM stages.

In briefest outline, the comparison pipeline takes the following steps:

Profile HMM filter stages: The first several stages of the pipeline use only a profile HMM, putting off all calculations with the CM until later. Since profile HMM algorithms are less complex than CM ones, this saves time by only using the expensive CM methods for regions the HMM has identified as having a good chance of containing a high-scoring hit. Of course, by relying on sequence-only based filters like HMMs, we are potentially going to miss homologs that are divergent at the sequence level but that the CM would still score highly thanks to conserved secondary structure. Our benchmarks reveal this is rare, but it does happen and is an important failure mode to keep in mind.

The profile HMM filter stages are very closely based on the similar steps in HMMER3's accelerated comparison pipeline with the important difference that both *local* and *glocal* versions of HMM algorithms are used.

Here's a list of the profile HMM filter stages:

Null model. Calculate a score term for the "null hypothesis" (a probability model of *non*-homology). This score correction is used to turn all subsequent profile/sequence bit scores into a final log-odds bit score.

Local scanning SSV filter. The SSV ("Single Segment Viterbi") algorithm looks for high-scoring *ungapped* alignments. Each sequence segment (referred to as a "diagonal") in a SSV alignment is extended slightly to define a window. Overlapping windows are merged together into a single window. Then, long windows greater than a maximum length $2L$ (where L is the maximum of the model's W parameter[1] and 1.25 times the consensus length of the model) are split into multiple windows of length $2L$ with $L-1$ overlapping nucleotides between adjacent windows. Each window is then passed on to the next next pipeline step. Any nucleotides that are not contained in an SSV window are not evaluated further.

Local Viterbi filter. A more stringent accelerated filter. An optimal (maximum likelihood) gapped alignment score is calculated for each sequence window that survived SSV. If this score passes a set threshold, the sequence passes to the next step; else it is rejected.

Bias filter. A hack that reduces false positive Viterbi hits due to biased composition sequences. A two-state HMM is constructed from the mean nucleotide composition of the profile HMM and the standard nucleotide composition of the null model, and used to score the sequence window. The Viterbi bit score is corrected using this as a second null hypothesis. If the Viterbi score still passes the Viterbi threshold, the sequence passes on to the next step; else it is rejected. *The bias filter score correction will also be applied to the local Forward filter and glocal Forward filter scores that follow.*

Local Forward filter. The full likelihood of the profile/sequence window comparison is evaluated, summed over the entire alignment ensemble, using the HMM Forward algorithm with the HMM in *local* mode. This score is corrected to a bit score using the null model and bias filter scores. If the bit score passes a set threshold, the sequence window passes on to the next step; else it is rejected.

Glocal Forward filter/parser. The HMM Forward algorithm is again used to evaluate the full likelihood of the profile/sequence window comparison, but this time the HMM is configured in *global* mode, which requires

[1] W is the expected maximum hit length for the model calculated by `cmbuild` and stored in the CM file

that any valid alignment begin at the first consensus position (match or delete) and end at the final consensus position. (In local mode alignments can begin and end at any model positios.) Aligments in this stage, as in all previous stages can be local *with respect to the sequence* (starting and ending at any sequence position), which is why this stage is referred to as *glocal*: global with respect to the model and local with respect to the sequence. The glocal Forward score is corrected to a bit score using the null model and bias filter scores. If the bit score passes a set threshold, the sequence window passes on to the next step; else it is rejected.

Glocal envelope definition. Using the glocal Forward parser results, now combined with glocal Backward parser results, posterior probabilities that each nucleotide in the window is aligned to a position of the model are calculated. A discrete set of putative alignments is identified by applying heuristics to posterior probabilities. This procedure identifies *envelopes*: subsequences in the target sequence window which contain a lot of probability mass for a match to the profile. The envelopes are often significantly shorter than the full window, containing only nucleotides that have a signficant probability of aligning to the HMM, which is critical for the subsequent CM stages of the pipeline. Each envelope's (there can be more than one if the evaluation reveals multiple full length alignments in a single window) glocal Forward score is corrected to a bit score using the null model and bias filter scores. If the bit score passes a set threshold, the sequence envelope passes on to the next step; else it is rejected.

Covariance model stages: The remainder of the pipeline uses the CM to evaluate each envelope that survived the profile HMM filter stages.

HMM banded CYK filter. For each envelope, posterior probabilities that each sequence residue aligns to each state of a profile HMM is computed[2] and used to derive bands (constraints) for the CM CYK algorithm (Brown, 2000; Nawrocki, 2009). A banded version of the CYK algorithm is used to determine the bit score of the maximum likelihood alignment of any subsequence within the envelope to the CM that is consistent with the HMM-derived bands. If this score passes a set threshold, the sequence envelope passes on to the next step; else it is rejected. Additionally, the boundaries of the envelope may be modified (shortened: the start position increased and/or the end position decreased) at this stage, as detailed below.

HMM banded Inside parser. The full likelihood of the profile/sequence envelope is now evaluated, summed over the entire alignment ensemble for every subsequence of the envelope, using the CM Inside algorithm. Again HMM bands are used to constrain the CM dynamic programming calculations. This procedure identifies zero or more non-overlapping *hits*, each defined as a subsequence in the envelope. An *ad hoc* "null3" hypothesis is constructed for each hit's composition and used to calculate a biased composition score correction. The null3 score is subtracted from the Inside bit score and an E-value is computed for that score.

CM bias filter: the "null3" model. To reduce false positive CM hits due to biased composition sequences, the Inside score is adjusted using a bias filter that is similar, but not identical, to the one used by the HMM stages. A single state HMM is constructed with emission probabilities equal to the mean nucleotide composition of the sequence of the hit, and used to score the hit. The Inside bit score is corrected using this as a second null hypothesis.

Alignment. For each identified hit, the HMM banded Inside/Outside algorithm is performed and an optimal accuracy (also sometimes called maximum expected accuracy) alignment is calculated.

Storage. Now we have a *hit score* (and E-value) and each hit has an optimal accuracy alignment annotated with per-residue posterior probabilities.

Filter thresholds are dependent on database size

Before we go into more detail on each stage, we'll briefly discuss the filter thresholds used for each stage of the pipeline. These are the P-values required for a window or envelope to pass each stage of the pipeline. Unlike in HMMER, in which the filter thresholds are always the same regardless of the query and target, in Infernal, the HMM filter thresholds

[2] The profile HMM used at this stage is referred to as a CM plan 9 (CP9) HMM in it the code. It is similar but not identical to the one used for filtering. It was constructed to be maximally similar to the CM and includes transitions between insert and delete states not allowed in the HMMER plan 7 models used in the filtering steps. CP9 HMMs are essentially a reimplementation of the maximum likelihood heuristic HMM described in (Weinberg and Ruzzo, 2006).

filter stage	model	model configuration	size of search space (Z) < 2Mb	2Mb to 20Mb	20Mb to 200Mb	200Mb to 2Gb	2Gb to 20Gb	> 20Gb
SSV	HMM	local	0.35	0.35	0.35	0.15	0.15	0.06
Viterbi	HMM	local	off	off	0.15	0.15	0.15	0.02
Forward	HMM	local	0.02	0.005	0.003	0.0008	0.0002	0.0002
Forward	HMM	global	0.02	0.005	0.003	0.0008	0.0002	0.0002
envelope definition	HMM	global	0.02	0.005	0.003	0.0008	0.0002	0.0002
HMM banded CYK	CM	local	0.0001	0.0001	0.0001	0.0001	0.0001	0.0001
HMM banded Inside	CM	local	E $\leq$ 10	E $\leq$ 10	E $\leq$ 10	E $\leq$ 10	E $\leq$ 10	E $\leq$ 10
Average relative running time per Mb			30.0	10.0	5.0	2.5	1.5	1.0

Table 1: **Default P-value survival thresholds used for each filter stage for different search space sizes Z.** These values can be changed with command-line options as described in the text. The "HMM banded Inside" stage is not actually a filter, hits with an E-value $\leq$ 10 after this stage are reported to the search output. The final line "Average relative running time" provides rough estimates of the relative speed per Mb of the different parameter settings for each range of Z (these are relative units, not an actual unit of time like minutes). Importantly, Z is defined differently in `cmsearch` and `cmscan`. In `cmsearch`, Z is the total number of nucleotides in the target database file multiplied by 2 (because both strands of each sequence is searched). For `cmscan`, Z is the length of the current query sequence multiplied by 2 (because both strands of the sequence are searched) and multiplied again by the number of CMs in the target CM database.

are dependent on the size of the search space. We use the parameter Z in the code and in this documentation to represent the search space.

For `cmsearch`, Z is the size of the target sequence database, in total number of nucleotides, multiplied by 2 because both strands of each sequence will be searched. For `cmscan`, Z is defined differently; it is the length of the current query sequence (again, multiplied by 2) in nucleotides *multiplied by the number of models in the target CM database*.

In general, the larger the search space, the stricter the filter thresholds become. The specific thresholds used for all stages of the pipeline for all possible search space sizes Z are given in Table 1.

The rationale for making filter thresholds more strict as Z increases is as follows. As Z increases so too does the CM bit score necessary for a hit to reach the E-value reporting threshold (by default 10.0). If we assume that a hit's filter HMM score increases with its CM score (which should be true for most true homologs), then it follows that we should be able to decrease filter P-value thresholds as Z increases without sacrificing sensitivity relative to an unfiltered search. Of course, it's unclear exactly how much we can decrease the thresholds by before we start losing an unacceptable amount of sensitivity. We've taken an empirical approach to determine this by measuring performance on an internal benchmark of remote structural RNA homology search based on Rfam. The sets of filter thresholds in Table 1 were determined to achieve what we feel is a good trade-off between sensitivity, specificity and speed on that benchmark.

Manually setting filter thresholds

As described above, by default filter thresholds are automatically set based on the search space Z, but several options exist for overriding these defaults. There are four pre-defined sets of thresholds, each available via a single command-line option to `cmsearch` or `cmscan`. In order from least strict to most strict these are: `--max`, `--nohmm`, `--mid`, and `--rfam`. (The default thresholds lie somewhere between `--mid` and `--rfam`.) Additionally, you can specify that

the filter thresholds be set as if the search space was <x> megabases with the `--FZ <x>` option. More detail on each of these options is provided below. A one-line description of each option is printed as part of the `cmsearch` and `cmscan` 'help' output that gets displayed with the use of the `-h` option. There's also descriptions of these options in the `cmsearch` and `cmscan` manual pages.

--max: Turn off all filters, run Inside on full length target sequences. This option will result in maximum sensitivity but is also the slowest. Using this option will slow down `cmsearch` by about 3 or 4 orders of magnitude for typical searches.

--nohmm: Turn off all profile HMM filters, run only the CYK filter on full length sequences and Inside on surviving envelopes. Searches with this option will often be between 5 and 10 times faster than `--max` searches.

--mid: Turn off the SSV and Viterbi filters, and set all profile HMM filter thresholds (local Forward, glocal Forward and glocal domain definition) to the same, set P-value. By default this P-value is 0.02, but it is settable to <x> by also using the `--Fmid <x>` option.

--rfam: Set all filter thresholds as if the search space were more than 20 Gb. These are the filter thresholds used by a database like Rfam, which annotates RNAs in an EMBL-based sequence dataset that is several hundred Gb. If you're trying to reproduce results in future versions of Rfam that use Infernal 1.1 (of course, at the time of writing, no version of Rfam yet exists which has used version 1.1) you probably want to use this option. This option will have no effect if the target search space is more than 20 Gb.

--FZ <x>: Set the filter thresholds as if the search space were <x> Mb instead of its actual size. Importantly, the E-values reported will still correspond to the actual size. (To change the search space size the E-values correspond to, use the `-Z <x2>` option[3].)

For expert users, options also exist to precisely set each individual filter stage's threshold. Each stage is referred to by an option that begins with `F` and ends with a number for the stages position in the pipeline (SSV is F1, local Viterbi is F2, and so on). Also, options that pertain to the bias filter part of each stage end in `b`. The complete list of options for controlling the filter thresholds is: `--F1`, `--F1b`, `--F2`, `--F2b`, `--F3`, `--F3b`, `--F4`, `--F4b`, `--F5`, `--F5b`, and `--F6`. Additional options exist for turning on or of each stage as well: `--noF1`, `--doF1b`, `--noF2`, `--noF2b`, `--noF3`, `--noF3b`, `--noF4`, `--noF4b`, `--noF5`, `--noF5b`, and `--noF6`. Because these options are only expected to be useful to a small minority of users, they are only displayed in the help message for `cmsearch` or `cmscan` if the special `--devhelp` option is used.

In more detail: profile HMM filter stages

Now we'll go back and revisit each stage of the pipeline in a bit more detail.

Null model.

The "null model" calculates the probability that the target subsequence (window or envelope) is *not* homologous to the query profile. A profile HMM or CM bit score is the log of the ratio of the sequence's probability according to the profile (the homology hypothesis) over the null model probability (the non-homology hypothesis).

The null model is a one-state HMM configured to generate "random" sequences of the same mean length L as the target subsequence window or envelope being evaluated[4], with each residue drawn from a background frequency distribution of 0.25 for all four RNA nucleotides (a standard i.i.d. model: residues are treated as independent and identically distributed). This null model is used for the profile HMM and the CM stages of the pipeline.

For technical reasons, the *residue emission* probabilities of the null model are incorporated directly into the profile HMM, by turning each emission probability in the profile into an odds ratio. The null model score calculation therefore is only concerned with accounting for the remaining *transition* probabilities of the null model and toting them up into a bit score correction. The null model calculation is fast, because it only depends on the length of the target sequence window being evaluated, not its sequence.

[3]Note that if you use `-Z <x2>` without `--FZ <x>`, the filter thresholds will be set as if the search space size is <x2> Mb.

[4]For the SSV filter, which examines full length target sequences instead of windows, L is set as the expected maximum length of a hit for the profile, defined as the maximum of the 1.25 times the consensus length of the model and the W parameter for the CM we're filtering for. The W parameter is calculated by `cmbuild`.

SSV filter.

The sequence is aligned to the profile using a specialized model that allows a single high-scoring local ungapped segment to match. The optimal alignment score (Viterbi score) is calculated under this specialized model, hence the term SSV, for "single-segment Viterbi". SSV is similar, but not identical to, the MSV ("multi-segment Viterbi) algorithm used by the programs in HMMER for protein sequence analysis. There are two main differences. First, SSV only allows a single ungapped segment match between the sequence and specialized model. Second, the variant of SSV used by Infernal is designed for scanning along potentially long sequences (think chromosomes instead of protein sequences) and potentially finding many high-scoring hits in each sequence. This scanning SSV algorithm was developed by Travis Wheeler for a soon-to-be released version of HMMER that includes a program for DNA homology search called `nhmmer`.

Vector parallelization methods are used to accelerate optimal ungapped alignment in SSV. The P-value threshold for what subsequences pass this filter range from 0.35 for small target databases down to 0.06 for large ones (Table 1). Take as an example, the `cmsearch` for tRNAs performed in the tutorial. The database size was roughly 6 Mb, so the SSV threshold was set as 0.35. This means that about 35% of nonhomologous sequence is expected to pass.

The SSV bit score is calculated as a log-odds score using the null model for comparison. No correction for a biased composition or repetitive sequence is done at this stage. For comparisons involving biased sequences and/or profiles, more than 35% of comparisons will pass the SSV filter. For the tRNA search from the tutorial, the end of the search output contained a line like:

```
Windows   passing  local HMM SSV           filter:           11197  (0.2108); expected (0.35)
```

which tells you how many windows and what fraction of the total database was comprised by those windows passed the SSV filter, versus what fraction was expected.

The `--F1 <x>` expert option sets filter P-value threshold for passing the SSV filter to `<x>`. The `--doF1b` and `--F1b <x>` options turn on a SSV bias filter (described further below) and control its P-value threshold, respectively. SSV is turned off if the `--max`, `--nohmm`, `--mid`, or `--noF1` options are used.

Local Viterbi filter.

Each sequence window that survives the SSV filter is now aligned to the profile HMM using a fast Viterbi algorithm for optimal gapped alignment. This stage is identical the Viterbi stage of the HMMER3 pipeline.

This Viterbi implementation is specialized for speed. It is implemented in 8-way parallel SIMD vector instructions, using reduced precision scores that have been scaled to 16-bit integers. Only one row of the dynamic programming matrix is stored, so the routine only recovers the score, not the optimal alignment itself. The reduced representation has limited range; local alignment scores will not underflow, but high scoring comparisons can overflow and return infinity, in which case they automatically pass the filter.

The final Viterbi filter bit score is then computed using the null model score.

As with SSV, and all other HMM filter stages, the local Viterbi filter threshold depends on the search space Z. It is the only stage that is actually turned off if Z is low enough (less than 20 Mb). For the tRNA search used in the tutorial Z was less than 20 Mb, so the local Viterbi filter was turned off, and the following line was included in the output in the pipeline summary output:

```
Windows   passing  local HMM Viterbi       filter:                  (off)
```

For searches with Z greater than 20 Mb, this line would be formatted similar to the one for the SSV filter. For example, a search of the tRNA model from the tutorial against the *Saccharomyces cerevisiae* genome results in:

```
Windows   passing  local HMM Viterbi       filter:           20666  (0.09861); expected (0.15)
```

In this search 20666 windows have survived the local Viterbi filter, comprising 0.09861 fraction of all nucleotides searched. The expected survival fraction was 0.15. This large of a deviation from expectation is common. One reason for it is that the 0.15 expectation assumes that the full (100%) of the database is subjected to the Viterbi filter, but remember that only the fraction that survived SSV will be (roughly 35%). You might expect that in general most of the windows that would eventually survive Viterbi would also survive SSV, but surely some of them will fail to pass SSV which will lower the expectation from 0.15. Exactly how much it will lower it is based on many factors and is probably difficult to predict accurately - Infernal doesn't even try. So while 0.15 is printed as the expectation, you will often observe survival fractions substantially lower than this. This same logic applies to all downstream filter stages. There are other factors at play here too, including biased composition effects, which will also impact the accuracy of the survival fraction predictions of all other stages of the pipeline.

The --F2 <x> option controls the P-value threshold for passing the Viterbi filter, and can be turned off with --noF2. The local Viterbi filter is also turned off if the --max, --nohmm, or --mid options are used.

Biased composition filter.

It's possible for profile HMMs and/or sequences to have biased nucleotide compositions that result in "significant" log-odds bit scores not because the profile matches the sequence particularly well, but because the *null model* matches the sequence particularly badly.

In a few cases, profiles and/or target sequences are sufficiently biased that too many comparisons pass the local Viterbi or local Forward stages of the filter pipeline, causing Infernal speed performance to be severely degraded. The treatment of biased composition comparisons is a serious problem for the HMM implementations in HMMER and Infernal. Solving it well will require more research. As a stopgap solution to rescuing most of the speed degradation while not sacrificing too much sensitivity, an *ad hoc* biased composition filtering step is applied to remove highly biased comparisons.

On the fly, a two-state HMM is constructed. One state emits nucleotides from the background frequency distribution (same as the null1 model), and the other state emits nucleotides from the mean nucleotide composition of the profile (i.e. the expected composition of sequences generated by the core model, including match and insert states [hmmer/src/p7_hmm.c:p7_hmm_SetComposition()]). Thus if the profile is highly biased (A-rich, for example), this composition bias will be captured by this second state. This model's transitions are arbitrarily set such that state 1 emits an expected length of the size of the current window, and state 2 emits an expected length of M/8 at a time (for a profile HMM of consensus length M). An overall target sequence length distribution is set to a mean of L, identical to the null1 model.

The sequence is then rescored using this "bias filter model" in place of the null1 model, using the HMM Forward algorithm. A new local Viterbi bit score is obtained. If the P-value of this satisfies the local Viterbi bias filter threshold, the sequence passes to the next stage of the pipeline.

The bias filter score is used in an analogous way in the next two stages of the pipeline: the local Forward and glocal Forward stages. The biased composition filter step compromises a small amount of sensitivity. Though it is good to have it on by default, you may want to shut it off if you know you will have no problem with biased composition hits.

The default value used for the local Viterbi bias filter depends on the search space size, Z. Like the local Viterbi filter it is turned off by default if Z is less than 20 Mb. So it too was off in the example tRNA search from the tutorial, and the summary line in the output was:

```
Windows   passing  local HMM Viterbi  bias filter:                 (off)
```

For searches with Z greater than 20 Mb, this line would be formatted similar to the one for the SSV filter. For example, a search of the tRNA model from the tutorial against the *Saccharomyces cerevisiae* genome results in:

```
Windows   passing  local HMM Viterbi  bias filter:           20342  (0.09712); expected (0.15)
```

So 20342 windows survive this stage, making up 0.09712 fraction of the total sequence space searched. A similar line is included above for the (non-bias) local HMM Viterbi filter, indicating that 20666 windows passed that stage, meaning that 324 windows were removed by the Viterbi bias filter.

The --F2b <x> option controls the P-value threshold for passing the local Viterbi bias filter stage. The --noF2b option turns off (bypasses) the local Viterbi biased composition filter. With this option, the local Viterbi filter will still be used, but its score is not recomputed using the bias composition model. The local Viterbi bias filter is also turned off if the --max or --nohmm options are used.

Local Forward filter.

Each surviving window is now aligned to the profile HMM using the full Forward algorithm with the model configured in local mode (allowing alignments to begin and end at any position of the model), which calculates the likelihood of the target sequence given the profile, summed over the ensemble of all possible alignments. This step is identical to the "Forward filter/parser" stage of the HMMER pipeline.

This is a specialized time- and memory-efficient Forward implementation called the "Forward parser". It is implemented in 4-way parallel SIMD vector instructions, in full precision (32-bit floating point).

The local Forward filter bit score is calculated by correcting this score using the standard null model log likelihood. If the P-value of this score passes the local Forward filter threshold then it passes to the Forward bias filter and the score

is recomputed using the biased composition model. If the P-value of this score passes the Forward bias filter threshold, then this window is pass onto the next stage of the pipeline[5].

The local Forward filter threshold used depends on the search space Z (Table 1). For the tRNA search in the tutorial, Z was about 6 Mb and the threshold for the local Forward stage and its bias filter were set as 0.005. The summary output contains two lines of output pertaining to this stage:

```
Windows   passing  local HMM Forward       filter:             140  (0.002747); expected (0.005)
Windows   passing  local HMM Forward  bias filter:             139  (0.002728); expected (0.005)
```

For this search 140 windows survived the local HMM Forward filter, and one of these was removed by the subsequent bias filter.

The `--F3 <x>` and `--F3b <x>` control the P-value thresholds for passing the local Forward and local Forward bias filter stages. The `--noF3` and `--noF3b` options turn off (bypasses) the stages. Both filters are also turned off if the `--max` or `--nohmm` options are used.

Glocal Forward filter.

Each surviving window is now aligned to the profile HMM again using the full Forward algorithm, but this time with the model configured in global mode, only allowing alignments that begin at the first position of the model and end at the final position. This stage is referred to as *glocal* because it is global with respect to the profile HMM, but (potentially) local with respect to the sequence window, alignments can begin and end at any position in the sequence window.

For technical reasons related to the inability to guarantee a lower bound on the glocal Forward score, glocal Forward is not implemented with parallel SIMD vector instructions, and consequently it is significantly slower than the local Forward filter implementation. However, in practice it is run on a significantly smaller fraction of the database than is the local stage, which decreases the impact of this slower speed on the overall running time of the search.

It is not immediately obvious that using a glocal HMM filter stage is a good idea, when our final CM stages of the pipeline will allow local hits to the model. You might imagine that the glocal filter will remove some windows that don't match well enough to the *full* model to survive but do contain a high-scoring local CM hit. This is undoubtedly true in some cases (indeed, we could certainly construct examples of this) but it does not seem to be common enough to be a concern, at least based on our internal benchmarking. That is, using both a local and glocal Forward filter does not seem to decrease sensitivity compared with only using a local one. The reason probably lies in the relatively high (0.005) filter threshold used at this stage. Even hits that will eventually be reported as local hits still contain surrounding sequence similar enough to the profile to make up a global score that passes threshold.

An obvious failure mode of the glocal filter is for identifying hits that are truncated at one or both ends due to the beginning or termination of a sequencing read. However, Infernal uses a special pipeline for identifying these hits at the ends of sequences, as described in a later section, so if they're missed at this stage, they should be detected later when the truncated variants of the pipeline are run.

The glocal Forward filter bit score is calculated by correcting the score found by the Forward algorithm using the standard null model log likelihood. If the P-value of this score passes the glocal Forward filter threshold then it passes to the glocal Forward bias filter and the score is recomputed using the biased composition model. If the P-value of this score passes the glocal Forward bias filter threshold, then this window is pass onto the next stage of the pipeline.

The glocal Forward filter threshold used depends on the search space Z (Table 1). For the tRNA search in the tutorial, Z was about 6 Mb and the threshold for the glocal Forward stage and its bias filter were set as 0.005. The summary output contains two lines of output pertaining to this stage:

```
Windows   passing glocal HMM Forward       filter:              88  (0.001973); expected (0.005)
Windows   passing glocal HMM Forward  bias filter:              88  (0.001973); expected (0.005)
```

For this search 88 of the 139 windows evaluated by the glocal HMM Forward filter survived, and none of them were removed by the subsequent bias filter.

The `--F4 <x>` and `--F4b <x>` control the P-value thresholds for passing the glocal Forward and glocal Forward bias filter stages. The `--noF4` and `--noF4b` options turn off (bypasses) the stages. Both filters are also turned off if the `--max` or `--nohmm` options are used.

[5] You might wonder why the intial score with just the null model is even computed if the bias adjusted score must also pass the threshold for the window to survive. We do this solely for more informative output - so we can report how many windows fail to pass at each of these two stages independently, to potentially alert users if a large number of windows are being thrown out by the bias filter alone.

Envelope definition.

A target sequence window that reaches this point is likely to be larger than the eventual hit or hit(s) (if any) contained within it, including nonhomologous nucleotides at the ends of the target sequence window and possibly in between hits (if there are more than one). At this stage, each window is transformed into one or more envelopes that usually are significantly shorter than the original window.

Infernal's HMM envelope definition step is very similar to the *domain* definition step of HMMER, with the important difference that the profile HMM is configured for global alignment instead of local alignment. The envelope definition step is essentially its own pipeline, with steps as follows:

Backward parser. The counterpart of the glocal Forward filter algorithm is calculated. The Forward algorithm gives the likelihood of all *prefixes* of the target sequence, summed over their alignment ensemble, and the Backward algorithm gives the likelihood of all *suffixes*. For any given point of a possible model state/residue alignment, the product of the Forward and Backward likelihoods gives the likelihood of the entire alignment ensemble conditional on using that particular alignment point. Thus, we can calculate things like the posterior probability that an alignment starts or ends at a given position in the target sequence.

Decoding. The posterior decoding algorithm is applied, to calculate the posterior probability of alignment starts and ends (profile B and E state alignments) with respect to target sequence position.

The sum of the posterior probabilities of alignment starts (B states) over the entire target sequence window is the *expected number of hits* in the sequence window.

Region identification. A heuristic is now applied to identify a *non-overlapping* set of "regions" that contain significant probability mass suggesting the presence of a match (alignment) to the profile.

For each region, the expected number of envelopes is calculated (again by posterior decoding on the Forward/Backward parser results). This number should be about 1: we expect each region to contain one global alignment to the profile.

Envelope identification. Now, within each region, we will attempt to identify envelopes. An envelope is a subsequence of the target sequence that appears to contain alignment probability mass for a likely hit (one global alignment to the profile).

When the region contains $\simeq$1 expected envelope, envelope identification is already done: the region's start and end points are converted directly to envelope coordinates.

In some cases, the region appears to contain more than one expected hit – where more than one hit is closely spaced on the target sequence and/or the domain scores are weak and the probability masses are ill-resolved from each other. These "multi-hit regions", when they occur, are passed off to an even more *ad hoc* resolution algorithm called *stochastic traceback clustering*. In stochastic traceback clustering, we sample many alignments from the posterior alignment ensemble, cluster those alignments according to their overlap in start/end coordinates, and pick clusters that sum up to sufficiently high probability. Consensus start and end points are chosen for each cluster of sampled alignments. These start/end points define envelopes.

It's also possible (though rare) for stochastic clustering to identify *no* envelopes in the region.

During envelope identification, the Forward algorithm is used to score each putative envelope, and the standard null model (not the bias one) is used as a correction (using mean length L equal to the envelope length) and this score is checked to see if it is below a P-value threshold. This threshold is Z-dependent (Table 1. For the tRNA search in the tutorial, this threshold is 0.005. If the P-value is less than the threshold the envelope has survived all HMM filter stages and is passed onto the CYK stage of the filter pipeline.

In the tRNA search example, the summary output line for the envelope definition stage is shown below (the line for the previous filter stage is also shown for comparison):

```
Windows   passing glocal HMM Forward  bias filter:              88  (0.001973); expected (0.005)
Envelopes passing glocal HMM envelope defn filter:             101  (0.001358); expected (0.005)
```

Note that while only 88 windows were passed into this stage, comprising 0.001973 fraction of the total database, 101 envelopes survived making up 0.001358 fraction of the database survived. Some windows have been transformed into multiple envelopes and in general the surviving envelopes are significantly shorter than the input windows.

The `--F5 <x>` expert option sets the filter P-value threshold for passing the envelope definition filter to `<x>`. The `--doF5b` and `--F5b <x>` options turn on a bias filter for this stage and control its P-value threshold, respectively. HMM envelope definition is turned off if the `--max` or `--nohmm` options are used.

In more detail: CM stages of the pipeline

After all profile HMM filter stages are complete, we have defined a set of envelopes each of which may contain a single hit to the CM. We now using sequence- and structure-based CM algorithms to score each envelope and determine if it has a significant (reportable) hit to the CM within it. Unfortunately, CM algorithms are computationally expensive (more than an order of magnitude more complex than HMM algorithms) so we need to constrain these algorithms in order to incorporate them into a comparison pipeline that runs at a practical speed. The acceleration technique used by Infernal relies on computing and imposing bands on the CM dynamic programming matrices derived from an HMM Forward/Backward decoding of the sequence, similar to the one used in the envelope definition filter stage. This technique is based on one pioneered by Michael Brown (Brown, 2000). Next, we'll discuss the calculation of those bands and how they're utilized to accelerate the remaining CM stages of the pipeline.

HMM band definition for CM stages.

For each envelope that survives all the filter HMM stages, sequence-specific bands are derived for a CYK dynamic programming alignment of the envelope. The bands are derived from a HMM Forward/Backward/Decoding steps (similar to those used in the envelope definition pipeline) are used to determine the posterior probability that each HMM position aligns to each sequence position of the envelope. From these probabilities, *bands* are determined for each state, defined as sequence coordinate ranges that exclude only a preset amount of the probability mass for that state. By default, the excluded probability mass (referred to as tau (τ) in the code) is 0.0001 ($1e-4$). For example, a band for state x defined as positions 10 to 20 (inclusive) of an envelope of length 30 indicates that the summed probability of state x aligning to positions 1 through 9 and 21 through 30 is less than $1e-4$. These HMM bands are then mapped onto the CM by converting them to/from analogous states in the HMM and CM that model the same position in the consensus model. A more detailed discussion of the calculation and mapping of these bands can be found in (Nawrocki, 2009).

The HMM used for deriving these bands is not the same one used in the filter HMM stages of the pipeline. The new HMM used here is called a CM Plan 9 (CP9) HMM, and it is constructed to be maximally similar to a CM. These HMMs are essentially a reimplementation of of the maximum likelihood heuristic HMM described in (Weinberg and Ruzzo, 2006). CP9 HMMs are more similar to CMs than are the plan 7 HMMs of HMMER in that they include transitions from delete states to insert states and vice versa. These transitions are allowed in CMs, but not in plan 7 HMMs. Also, CP9 HMMs have special states for mirroring the local end (EL) state of a CM, which don't occur in plan 7 HMMs. Finally, these CP9 HMMs are configured in a local mode with an uneven begin and end probability distribution that places the highest probability on beginning at model position 1 and ending at the final model position. This local configuration more closely matches the CM local configuration than both the local and global p7 filter HMM configuration[6].

HMM banded CM CYK filter.

Given an envelope and CP9 HMM-derived bands constraining possible alignments of a CM to the sequence positions in the envelope, a banded version of the CM CYK algorithm is used to determine the maximum-likelihood CM alignment[7] of any subsequence in the envelope to the CM that is consistent with the HMM bands (Nawrocki, 2009).

In most cases that occur in Infernal's pipeline, the HMM banded version of CYK is significantly faster than non-banded, or even query-dependent banded (Nawrocki and Eddy, 2007) implementations of the CM CYK algorithm. The speedup gained typically increases with the consensus length of the model being used. However, HMM banded CYK is only efficient when the target sequence alignment with an HMM is well-resolved (includes at least some sequence position/model position pairs that align with high probability), because only then will the resulting bands be "tight" and exclude a large fraction of possible alignments. For this reason, the HMM filter stages of the pipeline, in particular the envelope definition stage, are crucial for enabling the HMM banded CM stages to work efficiently.

The null-corrected CYK bit score (corrected using the same type of equiprobable (25% A, C, G and U) iid, null model used by the profile HMM filter stages) is returned from the HMM banded CYK algorithm. No bias correction is used at this stage. If this score has a P-value less than 0.0001 then the envelope is passed to the next stage of the pipeline. Unlike in the HMM filter stages, the P-value threshold used for the CYK filter is not dependent on the search space Z, but it is controllable with the `--F6` option.

[6] In the plan 7 HMMER3 HMMs used in the filter stages, begin and end probabilities are equal for all positions of the model (Eddy, 2008).

[7] The CYK algorithm is the CM analog of the HMM Viterbi algorithm.

Envelope boundaries are potentially redefined at this stage based on CYK scores. Envelopes can only be shortened, not extended, at either boundary. We take advantage of the fact that the CYK algorithm computes the maximum likelihood alignment score (consistent with the HMM bands) of all possible subsequences in the envelope to the CM, and examine each nucleotide to see if it is included in any possible alignment to the model above a certain score threshold. By default, this threshold is a 10-fold lower P-value than the filter survival threshold, so it is 0.001 $(10 * 0.0001)$. This envelope redefinition can be turned off with the `--nocykenvx` option and it the threshold for it can be changed with the `--cykenvx <x>` option.

HMM banded CM Inside filter/parser.

Envelopes that survive CYK are evaluated using the CM Inside algorithm, which is the CM analog of the HMM Forward algorithm. HMM bands are rederived for this step (remember the envelope boundaries may now be different), using a smaller τ value of $5e-6$. With Inside, the probability of all possible alignments (instead of just the most likely one) of each subsequence to the model that is consistent with the bands are summed to define a probability for that subsequence. A log-odds score is determined by applying two null models: the standard null model used in CYK and an alternative null3 model based on the composition of the aligned subsequence. The null3 model is explained below.

A non-overlapping set of subsequence/model hits that fall below the reporting E-value threshold is then collected using a greedy approach[8]. Each hit is defined by a start/end position in the envelope.

By default, the reporting threshold is an E-value of 10.0, but this is settable to a different E-value with the `-E <x>` option, or to a particular bit score with the `-T <x>` option.

Optimal accuracy alignment.

For each hit reported by Inside, an optimally accurate alignment[9] is computed using HMM banded versions of the Inside and Outside algorithm (using the same bands from the Inside filter) and annotated with posterior probabilities for each aligned nucleotide. The optimally accurate alignment is the alignment that maximizes the sum of the posterior probabilities of all aligned nucleotides. The alignment is then stored in memory until the end of the search when it is finally output as part of a ranked list of hits and their alignments.

Biased composition CM score correction: the null3 model.

CM Inside scores are adjusted using an alternative null hypothesis called the null3 model in an effort to counteract the contribution of biased sequence composition to the scores of hits. The null3 model was motivated by the observation that many high-scoring false positive hits in CM searches are to regions of the target database with with highly biased residue composition, in particular regions with high percentages of A and U residues. If a model has a bias for a particular residue, for example A, and a target region is composed of an overrepresentation of that residue then it will receive a high score simply because it is A-rich.

A different null3 model is calculated for every alignment. The 4 emission probabilities of the null3 model are calculated as simply their occurence within the region of the hit. For example, if the hit is 50 residues long and contains 20 As, 5 Cs, 5 Gs and 20 Us, then the null3 model probabilitiles will be calculated as $(0.4, 0.1, 0.1, 0.4)$.

The null3 model is similar to the bias filter model used in the profile HMM stages but it often gives more severe corrections for highly biased hits because it is a single HMM state with emission probabilities defined by the hit composition, not the model composition. Importantly, scores collected during model calibration for estimating E-value parameters in `cmcalibrate` are also corrected using the null3 model.

The null3 model is used in combination with the standard null model (single state equiprobable A,C,G and U, same as the profile HMM standard null model). We told you earlier that an Infernal bit score is the log of the ratio of the sequence's probability according to the profile over the null model probability, but how do we calculate this score if we have more than one null hypothesis? Infernal does a bit of algebraic sleight of hand here, to arrive at an additive correction to the original score that it calls the "null3 score correction".

---

[8]This greedy approach is as follows: while any hits below threshold that do not overlap with previously reported ones remain, select the top scoring one and report it

[9]This is the same type of alignment referred to as "maximum expected accuracy" in the HMMER documentation

Derivation of the null3 score correction We arrived at the parameters of the null3 model in a very *ad hoc* way. However, after that, the way Infernal arrives at the final bit score once the null3 parameters have been determined is clean (e.g. derivable) Bayesian probability theory. It is analagous to the way HMMER uses the *null2* score correction.

If we take the Bayesian view, we're interested in the probability of a hypothesis H given some observed data D:

$$P(H|D) = \frac{P(D|H)P(H)}{\sum_{H_i} P(D|H_i)P(H_i)},$$

an equation which forces us to state explicit probabilistic models not just for the hypothesis we want to test, but also for the alternative hypotheses we want to test against. Up until now, we've considered two hypotheses for an observed sequence D: either it came from our CM (call that model M), or it came from our null hypothesis for random, unrelated sequences (call that model N). If these are the only two models we consider, the Bayesian posterior for the model M is:

$$P(M|D) = \frac{P(D|M)P(M)}{P(D|M)P(M) + P(D|N)P(N)}$$

Recall that the log odds score (in units of bits) reported by Infernal's alignment algorithms is

$$s = \log_2 \frac{P(D|M)}{P(D|N)}.$$

Let's assume for simplicity that ***a priori***, the profile and the null model are equiprobable, so the priors $P(M)$ and $P(N)$ cancel. Then the log odds score s is related to the Bayesian posterior by a sigmoid function,

$$P(M|D) = \frac{2^s}{2^s + 1}.$$

(We don't have to assume that the two hypotheses are equiprobable; keeping these around would just add an extra $\pi = \log P(M)/P(N)$ factor to s. We'll reintroduce these prior log odds scores π shortly.)

The simple sigmoid relationship between the posterior and the log odds score suggests a plausible basis for calculating a score that includes contributions of more than one null hypothesis: **we desire a generalized score S such that:**

$$\frac{2^S}{2^S + 1} = P(M|D),$$

for *any* number of alternative hypotheses under consideration.

So, let N_i represent any number of alternative null models N_i. Then, by algebraic rearrangement of Bayes' theorem,

$$S = \log \frac{P(D|M)P(M)}{\sum_i P(D|N_i)P(N_i)}.$$

We saw above that Infernal internally calculates a log odds score s, of the model relative to the first null hypothesis. Let's now call that s_M, the alignment score of the model. Infernal extends that same scoring system to all additional competing hypotheses, calculating a log odds score relative to the first null hypothesis for any additional null hypotheses $i > 1$:

$$s_i = \log \frac{P(D|N_i)}{P(D|N_1)}$$

We can also state prior scores π_i for how relatively likely each null hypothesis is, relative to the main one:

$$\pi_i = \log \frac{P(N_i)}{P(N_1)}$$

(Remember that we assumed $\pi_M = 0$; but we're going to put it back in anyway now.)

Now we can express S in terms of the internal scores s and prior scores π:

$$S = \log \frac{e^{s_M + \pi_M}}{1 + \sum_{i>1} e^{s_i + \pi_i}},$$

which therefore simply amounts to an additive correction of the original score, $(s_M + \pi_M)$:

$$S = (s_M + \pi_M) - \log\left(1 + \sum_{i>1} e^{s_i + \pi_i}\right)$$

So, to calculate its reported score, Infernal uses four quantities:

s_M The (simple, uncorrected) log odds score for the model, calculated by optimal alignment of the model to the sequence.

π_M The log odds of the priors, $\log P(M)/P(N_1)$. Infernal implicitly assumes this factor to be 0.

s_2 The (simple, uncorrected) log odds score for the null3 hypothesis, calculated by rescoring the residues of the alignment under the null3 model.

π_2 The log odds of the priors, $\log P(N_2)/P(N_1)$. Infernal arbitrarily assumes that the null3 model is $\frac{1}{65536}$ as likely as the main null model, so this factor is -16 bits.[10]

The code that calculates the null3 correction is in `cm_parsetree.c:ScoreCorrectionNull3()`.

The null3 correction is usually close to zero, for random sequences, but becomes a significant quantitative penalty on biased composition sequences. It gets added to the original alignment score to form Infernal's final bit score.

The null3 score correction is introduced in version 1.0 and was not present in any of the 0.x versions of Infernal. This can lead to large differences in the scores reported by 1.0 and previous versions.

The following table shows the penalty for a 100 nucleotide hit with varying compositions of A, C, G, and U residues. This table is included to give you an idea of how severe the null3 correction is, and can be useful for comparing bit scores from Infernal 1.0 to previous versions (which did not use the null3 correction). These are just a sample of the possible composition of hits you might see. Again, these scores are for 100 nucleotide hits, to determine the correction for a hit of length x simply multiply the corresponding correction below by $x/100$. For example, a 35% A, 15% C, 15% G, 35% U hit of length 100 nt would receive a 6.88 bit penalty from null3 (row 4). A 200 nt hit of the same composition would receive a penalty of 13.76 bits. A 50 nt hit of the same composition would receive a 3.44 bit penalty.

GC%	A%	C%	G%	U%	NULL3 correction (bits)
0.0	50.0	0.0	0.0	50.0	95.00
10.0	45.0	5.0	5.0	45.0	48.10
20.0	40.0	10.0	10.0	40.0	22.81
30.0	35.0	15.0	15.0	35.0	6.88
35.0	32.5	17.5	17.5	32.5	2.01
40.0	30.0	20.0	20.0	30.0	0.30
45.0	27.5	22.5	22.5	27.5	0.07
50.0	25.0	25.0	25.0	25.0	0.04
55.0	22.5	27.5	27.5	22.5	0.07
60.0	20.0	30.0	30.0	20.0	0.30
65.0	17.5	32.5	32.5	17.5	2.01
70.0	15.0	35.0	35.0	15.0	6.88
80.0	10.0	40.0	40.0	10.0	22.81
90.0	5.0	45.0	45.0	5.0	48.10
100.0	0.0	50.0	50.0	0.0	95.00

By default, the null3 score correction is used by `cmcalibrate`, `cmsearch` and `cmscan`. It can be turned off in any of these programs by using the `--nonull3` option. However, be careful, the E-values for models that are calibrated

[10]In versions 1.0 through 1.0.2, the null3 model was assumed to be $\frac{1}{32}$ as likely as the main null model (-5 bit factor instead of -16 bits). The decreased value in version 1.1 means that null3 penalties are reduced. We decided to do this based on internal benchmarking results. Version 1.1 uses a new default prior for parameterizing models which apparently alleviates the problem of biased composition, thus allowing us to reduce this value without sacrificing performance.

with --nonull3 are only valid when --nonull3 is also used with cmsearch or cmscan. Likewise, if --nonull3 is *not* used during calibration, it should not be used during searches or scans.

Truncated hit detection using variants of the pipeline

The Infernal pipeline discussed above is designed to find complete RNA homologs within a (usually) larger sequence, e.g. a choromosome. *Final* hits may be local (not full-length) with respect to the model, but the glocal HMM filter stage requires that at least some statistical signal remain in a full length match to the profile HMM. That is, enough signal to result in a good enough glocal Forward score to pass the glocal filter (e.g. a P-value of 0.005 for a search space between 2 and 20 Mb). In our benchmarking, the vast majority of statistically significant CM hits do pass the glocal Forward HMM filter, however, there is the obvious failure mode of the pipeline on *truncated* hits. A truncated hit is defined as a hit that is missing one or more nucleotides at the 5' and/or 3' end solely because of the position where the sequencing read happened to begin or end. While the chromosome the read derives from presumably included a full length hit, the read itself does not because the sequencing reaction used to determine the sequence began or ended at a position internal to the hit. Infernal uses modified versions of its profile/sequence comparison pipeline for detection of truncated hits. This modified pipeline is executed after the full sequence is examined with the standard pipeline.

The modified pipeline is only used near the sequence termini because Infernal only expects truncated hits to occur due to the premature ending of a sequencing read[11]. Specifically only the first or final X nucleotides at either sequence end are examined for truncated hits, where X is defined as the minimum of the W parameter (maximum expected hit length, calculated in cmbuild) and 1.25 times the consensus length of the model.

There are three variants of the pipeline used for truncated hit detection:

1. **5' truncated hit detection.** This pipeline is used only on the X 5'-most nucleotides of each sequence. These X nucleotides are also searched along with the rest of the complete sequence as part of the standard pipeline. Only 5' truncated or non-truncated alignments can be found by this pipeline variant, but non-truncated alignments will be duplicates of those found previously by the standard pipeline.
2. **3' truncated hit detection.** This pipeline is used only on the X 3'-most nucleotides of each sequence. These X nucleotides are also searched along with the rest of the complete sequence as part of the standard pipeline. Only 3' truncated or non-truncated alignments can be found by this pipeline variant, but non-truncated alignments will be duplicates of those found previously by the standard pipeline.
3. **5' and 3' truncated hit detection.** This pipeline is used only on complete sequences that are less than X nucleotides long. These sequences are also searched in their entirety by the standard pipeline, the 5' truncated variant pipeline, and the 3' truncated variant pipeline. Any types of alignments are possible at this stage (5' truncated, 3' truncated, 5' *and* 3' truncated or non-truncated) but only 5' *and* 3' truncated alignments will not be duplicates of previously found alignments.

Given a sequence to search, the standard pipeline described in the first part of this section is used on the full sequence. Then the truncated pipeline variants 1 and 2 are used on the sequence termini. Finally, if the sequence is less than X nucleotides long, variant 3 is used. Before hits are output, overlapping hits potentially found in different variants of the pipeline are removed, by keeping the highest scoring one. Also, remember that Infernal searches both strands of each sequence, so each pipeline is run separately on each strand.

It may seem like Infernal is duplicating effort by searching the first and final X nucleotides twice, i.e. once in the standard pipeline and once in the 5' truncated variant or the 3' truncated variant. One might argue that only searching these X nucleotides with the truncated pipeline variant should be sufficient to find truncated and non-truncated hits. However, as we'll discuss next, the glocal HMM filter stages, HMM band construction and the CYK and Inside algorithms are different between the standard and truncated variants of the pipeline and some non-truncated hits found by the standard variant will be missed by the truncated variants, and vice versa. So it is important we search the X terminal nucleotides on the 5' and 3' end using both standard and truncated variants of the pipeline. The same is true of sequences that are less than X nucleotides, which get searched four times, once by each of the four pipeline variants (the standard plus the three truncated variants).

[11] There are cases where truncated hits might occur within sequences ("split tRNAs" in archaea (Randau et al., 2005), for example), but these are rare and Infernal does not explicit look for these types of hits, unless the --anytrunc option is used, as discussed at the end of this section.

Differences between the standard pipeline and the truncated variants

For all three truncated variants of the pipeline, the first three HMM filter stages (SSV, local Viterbi and local Forward), as well as their bias composition corrections, are identical to the standard pipeline described above. However, the glocal HMM Forward, glocal HMM envelope definition, CYK and Inside differ to accomodate truncated hits.

Recall that in the standard pipeline in the glocal HMM Forward and envelope definition stages, the HMM is configured in global mode which forces all alignments to begin in the first model position and end in the final one (in the match or delete state in both cases). Also, in these stages, the alignment could begin or end in any position of the sequence window (i.e. local with respect to the sequence). These aspects of these two pipeline stages differ in the truncated variants. For the 5' truncated pipeline variant, alignments can begin at any model position but must end at the final model position, *and* the *first* nucleotide in the window must be aligned to an HMM model position. The 3' truncated pipeline variant does the opposite, allowing alignments to end at any position but requiring they start at the first position *and* requiring that the *final* nucleotide in the sequence be aligned to an HMM model position. In the 5' and 3' truncated pipeline variant, alignments can begin and end at any model position but the first and final nucleotide of the sequence window must both be aligned to model positions.

Similar changes are necessary for the truncated pipeline variants in the CM pipeline stages. The HMM bands are computed using CP9 HMMs configured in modified ways, similar to the modifications for the filter HMMs in the glocal filter stages. Specifically, in the 5' variant the first nucleotide in the window must be aligned to the model and model *begin* probabilities are equal for all model positions. In the 3' variant, the final nucleotide in the window must be aligned to the model and the model *end* probabilities are equal for all model positions. In the 5' and 3' variant, the first and final nucleotides (i.e. all nucleotides) in the window must be aligned to the model and model begin *and* end probabilities are equal for all model positions.

Also, additional score corrections are used in the CM stages to compensate for the fact that alignments are now allowed to begin and/or end at any model position to account for the truncation. The correction is roughly the log of $1/M$ bits for the 5'-only and 3'-only pipeline variants, and roughly the log of $2/(M*(M+1))$ bits for the 5' and 3' pipeline variant. For all hits found in a truncated pipeline variant, this correction is subtracted from the CM bit score.

For the CYK and Inside stages, specialized versions of the CM alignment algorithms are necessary to cope with truncated alignments. Allowing truncated alignments requires more changes to CM algorithms than for HMM algorithms because CMs are arranged in a tree-like structure instead of in a linear structure like HMMs, and need to be able to deal with the possibility that the sequence aligning to part of a subtree of the model has been truncated away. For example, a truncated CM alignment algorithm must be able to deal with the case where the left half but not the right half of a stem has been deleted, and vice versa. For details on CM truncated alignment, see (Kolbe and Eddy, 2009). HMM banded, truncated versions of CYK and Inside are implemented in Infernal, and are used by the truncated pipeline variants (see `src/cm_dpsearch_trunc.c` and `src/cm_dpalign_trunc.c`. These implementations allow either 5' truncation, 3' truncation, or both, and can require that valid alignments begin at the first nucleotide of the sequence or end at the final nucleotide of the sequence. For the 5' truncated pipeline variant, only 5' truncations are allowed and all valid alignments must begin with the first nucleotide of the sequence. For the 3' variant, only 3' truncations are allowed and all valid alignments must end with the final nucleotide of the sequence. For the 5' and 3' pipeline variant, 5' and/or 3' truncations are allowed, but all valid alignments must include all nucleotides of the sequence.

Modifying how truncated hits are detected using command-line options

The description above of the truncated variants of the pipeline describe Infernal's default behavior. You can turn off truncated hit detection with the `--notrunc` option. You can also modify how truncated hit detection works with the `--anytrunc` option. With `--anytrunc`, truncated hits are allowed to occur anywhere within a sequence, instead of necessarily including the first or final nucleotide.

Importantly, the truncated variants of the pipeline are currently not implemented for use with non-HMM banded alignment. This means that the they are incompatible with the `--max` options and `--nohmm` options. When either of these options are used, truncated hit detection is automatically turned off.

HMM-only pipeline variant for models without structure

As mentioned and demonstrated in the tutorial section of the guide, Infernal uses a special HMM-only pipeline for models that have zero basepairs. For a family with zero basepairs, it makes sense to use a profile HMM instead of a covariance model because they will be very similar models (the only important difference between CMs and profile HMMs is the former's ability to model two basepaired positions as dependent on one another) and HMM algorithms are

more efficient than CM ones. So a profile HMM search will be as sensitive but faster than a CM one for families with zero basepairs. When `cmsearch` or `cmscan` is being used for a comparison between a sequence and a model with zero basepairs, it will automatically use the HMM-only filter pipeline. For these models, the truncated variants of the pipeline are not used, because the standard HMM pipeline is capable of identifying truncated hits.

The HMM-only pipeline that Infernal uses is essentially identical to HMMER3's (version 3.0) pipeline, with the lone difference that the scanning local SSV filter (described for the standard pipeline above) replaces the full-sequence (non-scanning) MSV filter used in HMMER. The scanning SSV filter was originally implemented by Travis Wheeler for a new program in a soon-to-be-released version of HMMER called `nhmmer` for DNA homology search.

We won't go through the HMM-only pipeline in as much detail as the standard one (for more, see the HMMER3 user's guide (Eddy, 2009)), but briefly the HMM-only pipeline consists of the following steps: local scanning SSV filter to define windows, a bias filter to correct SSV scores for biased composition (identical to the one in the standard pipeline used after the local Viterbi stage), local Viterbi filter for each window, and finally local Forward filter for each window. Windows that survive the local Forward filter are then subject to the 'domain identification' stage of the HMMER pipeline, which identifies hits after full alignment ensemble calculation using the Forward and Backward algorithms. Each hit is then aligned to the HMM using an optimal accuracy alignment algorithm that maximizes the summed posterior probability of all aligned residues. The null3 biased composition model is not used in the HMM-only pipeline, but HMMER's null2 biased composition model is used, see the HMMER3 guide for details on null2.

Unlike in the standard pipeline, the filter thresholds used in the HMM-only pipeline are always the same, i.e. they are not dependent on the size of the search space. The default thresholds are the same values used in the HMMER3 pipeline: the SSV filter threshold is 0.02, the local Viterbi threshold is 0.001 and the local Forward threshold is $1e-5$. These can be changed with the `--hmmF1` (SSV), `--hmmF2` (Viterbi) and `--hmmF3` (Forward). The `--hmmmax` option runs the HMM-only pipeline in maximum sensitivity mode with the SSV P-value threshold set at 0.3 and the Viterbi and Forward filters turned off. The HMM-only pipeline can be turned off with the `--nohmmonly` option. When turned off, all models will use the standard and truncated CM pipelines, even those with no structure.

5 Profile SCFG construction: the `cmbuild` program

Infernal builds a model of consensus RNA secondary structure using a formalism called a *covariance model* (CM), which is a type of *profile stochastic context-free grammar* (profile SCFG) (Eddy and Durbin, 1994; Durbin et al., 1998; Eddy, 2002).

What follows is a technical description of what a CM is, how it corresponds to a known RNA secondary structure, and how it is built and parameterized.[1] You certainly don't have to understand the technical details of CMs to understand `cmbuild` or Infernal, but it will probably help to at least skim this part. After that is a description of what the `cmbuild` program does to build a CM from an input RNA multiple alignment, and how to control the behavior of the program.

Technical description of a covariance model

Definition of a stochastic context free grammar

A stochastic context free grammar (SCFG) consists of the following:

- M different nonterminals (here called *states*). I will use capital letters to refer to specific nonterminals; V and Y will be used to refer generically to unspecified nonterminals.
- K different terminal symbols (e.g. the observable alphabet, a,c,g,u for RNA). I will use small letters a, b to refer generically to terminal symbols.
- a number of *production rules* of the form: $V \rightarrow \gamma$, where γ can be any string of nonterminal and/or terminal symbols, including (as a special case) the empty string ϵ.
- Each production rule is associated with a probability, such that the sum of the production probabilities for any given nonterminal V is equal to 1.

SCFG productions allowed in CMs

A CM is a specific, repetitive SCFG architecture consisting of groups of model states that are associated with base pairs and single-stranded positions in an RNA secondary structure consensus. A CM has seven types of states and production rules:

State type	Description	Production	Emission	Transition
P	(pair emitting)	$P \rightarrow aYb$	$e_v(a,b)$	$t_v(Y)$
L	(left emitting)	$L \rightarrow aY$	$e_v(a)$	$t_v(Y)$
R	(right emitting)	$R \rightarrow Ya$	$e_v(a)$	$t_v(Y)$
B	(bifurcation)	$B \rightarrow SS$	1	1
D	(delete)	$D \rightarrow Y$	1	$t_v(Y)$
S	(start)	$S \rightarrow Y$	1	$t_v(Y)$
E	(end)	$E \rightarrow \epsilon$	1	1

Each overall production probability is the independent product of an emission probability e_v and a transition probability t_v, both of which are position-dependent parameters that depend on the state v (analogous to hidden Markov models). For example, a particular pair (P) state v produces two correlated letters a and b (e.g. one of 16 possible base pairs) with probability $e_v(a,b)$ and transits to one of several possible new states Y of various types with probability $t_v(Y)$. A bifurcation (B) state splits into two new start (S) states with probability 1. The E state is a special case ϵ production that terminates a derivation.

A CM consists of many states of these seven basic types, each with its own emission and transition probability distributions, and its own set of states that it can transition to. Consensus base pairs will be modeled by P states, consensus single stranded residues by L and R states, insertions relative to the consensus by more L and R states, deletions relative to consensus by D states, and the branching topology of the RNA secondary structure by B, S, and E states. The procedure for starting from an input multiple alignment and determining how many states, what types of states, and how they are interconnected by transition probabilities is described next.

[1] Much of this text is taken from (Eddy, 2002).

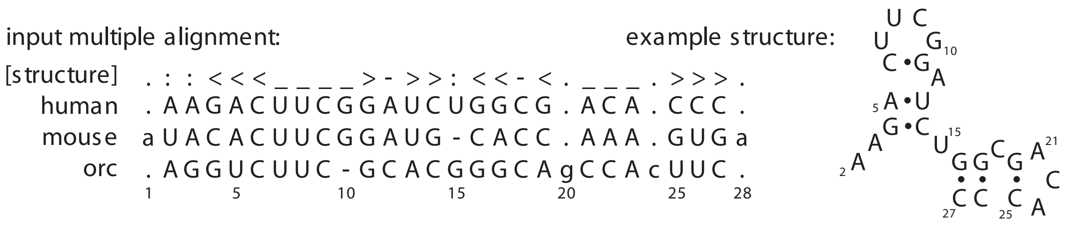

Figure 1: **An example RNA sequence family.** Left: a toy multiple alignment of three sequences, with 28 total columns, 24 of which will be modeled as consensus positions. The [structure] line annotates the consensus secondary structure in WUSS notation. Right: the secondary structure of the "human" sequence.

From consensus structural alignment to guide tree

Figure 1 shows an example input file: a multiple sequence alignment of homologous RNAs, with a line in WUSS notation that describes the consensus RNA secondary structure. The first step of building a CM is to produce a binary *guide tree* of *nodes* representing the consensus secondary structure. The guide tree is a parse tree for the consensus structure, with nodes as nonterminals and alignment columns as terminals.

The guide tree has eight types of nodes:

Node	Description	Main state type
MATP	(pair)	P
MATL	(single strand, left)	L
MATR	(single strand, right)	R
BIF	(bifurcation)	B
ROOT	(root)	S
BEGL	(begin, left)	S
BEGR	(begin, right)	S
END	(end)	E

These consensus node types correspond closely with the CM's final state types. Each node will eventually contain one or more states. The guide tree deals with the consensus structure. For individual sequences, we will need to deal with insertions and deletions with respect to this consensus. The guide tree is the skeleton on which we will organize the CM. For example, a MATP node will contain a P-type state to model a consensus base pair; but it will also contain several other states to model infrequent insertions and deletions at or adjacent to this pair.

The input alignment is first used to construct a consensus secondary structure (Figure 2) that defines which aligned columns will be ignored as non-consensus (and later modeled as insertions relative to the consensus), and which consensus alignment columns are base-paired to each other. For the purposes of this description, I assume that both the structural annotation and the labeling of insert versus consensus columns is given in the input file, as shown in the alignment in Figure 1, where both are are indicated by the WUSS notation in the [structure] line (where, e.g., insert columns are marked with `.`). (In practice, `cmbuild` does need secondary structure annotation, but it doesn't require insert/consensus annotation or full WUSS notation in its input alignment files; this would require a lot of manual annotation. More on this later.)

Given the consensus structure, consensus base pairs are assigned to MATP nodes and consensus unpaired columns are assigned to MATL or MATR nodes. One ROOT node is used at the head of the tree. Multifurcation loops and/or multiple stems are dealt with by assigning one or more BIF nodes that branch to subtrees starting with BEGL or BEGR head nodes. (ROOT, BEGL, and BEGR start nodes are labeled differently because they will be expanded to different groups of states; this has to do with avoiding ambiguous parse trees for individual sequences, as described below.) Alignment columns that are considered to be insertions relative to the consensus structure are ignored at this stage.

In general there will be more than one possible guide tree for any given consensus structure. Almost all of this ambiguity is eliminated by three conventions: (1) MATL nodes are always used instead of MATR nodes where possible, for instance in hairpin loops; (2) in describing interior loops, MATL nodes are used before MATR nodes; and (3) BIF

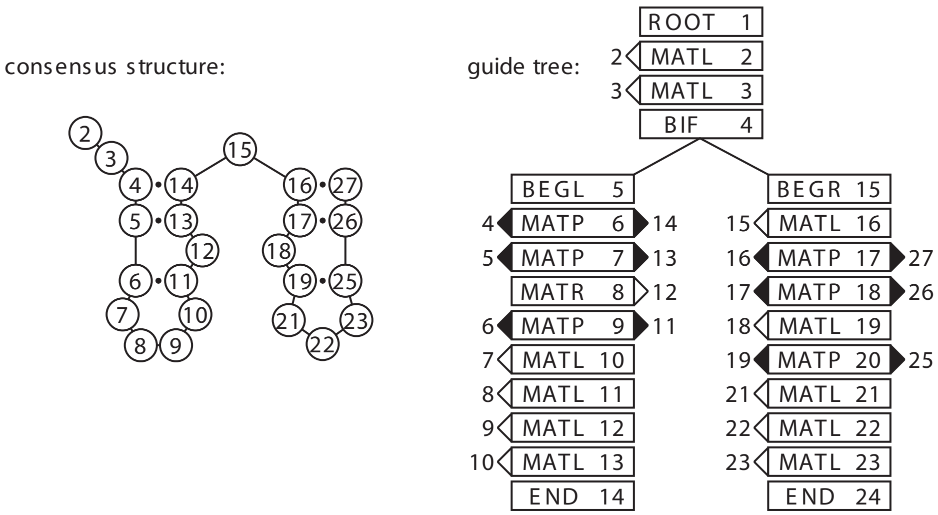

Figure 2: **The structural alignment is converted to a guide tree.** Left: the consensus secondary structure is derived from the annotated alignment in Figure 1. Numbers in the circles indicate alignment column coordinates: e.g. column 4 base pairs with column 14, and so on. Right: the CM guide tree corresponding to this consensus structure. The nodes of the tree are numbered 1..24 in preorder traversal (see text). MATP, MATL, and MATR nodes are associated with the columns they generate: e.g., node 6 is a MATP (pair) node that is associated with the base-paired columns 4 and 14.

nodes are only invoked where necessary to explain branching secondary structure stems (as opposed to unnecessarily bifurcating in single stranded sequence). One source of ambiguity remains. In invoking a bifurcation to explain alignment columns $i..j$ by two substructures on columns $i..k$ and $k+1..j$, there will be more than one possible choice of k if $i..j$ is a multifurcation loop containing three or more stems. The choice of k impacts the performance of the divide and conquer algorithm; for optimal time performance, we will want bifurcations to split into roughly equal sized alignment problems, so I choose the k that makes $i..k$ and $k+1..j$ as close to the same length as possible.

The result of this procedure is the guide tree. The nodes of the guide tree are numbered in preorder traversal (e.g. a recursion of "number the current node, visit its left child, visit its right child": thus parent nodes always have lower indices than their children). The guide tree corresponding to the input multiple alignment in Figure 1 is shown in Figure 2.

From guide tree to covariance model

A CM must deal with insertions and deletions in individual sequences relative to the consensus structure. For example, for a consensus base pair, either partner may be deleted leaving a single unpaired residue, or the pair may be entirely deleted; additionally, there may be inserted nonconsensus residues between this pair and the next pair in the stem. Accordingly, each node in the master tree is expanded into one or more *states* in the CM as follows:

Node	States	total # states	# of split states	# of insert states
MATP	[MP ML MR D] IL IR	6	4	2
MATL	[ML D] IL	3	2	1
MATR	[MR D] IR	3	2	1
BIF	[B]	1	1	0
ROOT	[S] IL IR	3	1	2
BEGL	[S]	1	1	0
BEGR	[S] IL	2	1	1
END	[E]	1	1	0

Here we distinguish between consensus ("M", for "match") states and insert ("I") states. ML and IL, for example, are both L type states with L type productions, but they will have slightly different properties, as described below.

The states are grouped into a *split set* of 1-4 states (shown in brackets above) and an *insert set* of 0-2 insert states. The split set includes the main consensus state, which by convention is first. One and only one of the states in the split set must be visited in every parse tree (and this fact will be exploited by the divide and conquer algorithm). The insert state(s) are not obligately visited, and they have self-transitions, so they will be visited zero or more times in any given parse tree.

State transitions are then assigned as follows. For bifurcation nodes, the B state makes obligate transitions to the S states of the child BEGL and BEGR nodes. For other nodes, each state in a split set has a possible transition to every insert state in the *same* node, and to every state in the split set of the *next* node. An IL state makes a transition to itself, to the IR state in the same node (if present), and to every state in the split set of the next node. An IR state makes a transition to itself and to every state in the split set of the next node.

There is one exception to this arrangement of transitions: insert states that are immediately before an END node are effectively *detached* from the model by making transitions into them impossible. This inelegant solution was imposed on the CM model building procedure to fix a design flaw that allowed an ambiguity in the determination of a parsetree given a structure. The detachment of these special insert states removes this ambiguity.

This arrangement of transitions guarantees that (given the guide tree) there is unambiguously one and only one parse tree for any given individual structure. This is important. The algorithm will find a maximum likelihood parse tree for a given sequence, and we wish to interpret this result as a maximum likelihood structure, so there must be a one to one relationship between parse trees and secondary structures (Giegerich, 2000).

The final CM is an array of M states, connected as a directed graph by transitions $t_v(y)$ (or probability 1 transitions $v \rightarrow (y,z)$ for bifurcations) with the states numbered such that $(y,z) \geq v$. There are no cycles in the directed graph other than cycles of length one (e.g. the self-transitions of the insert states). We can think of the CM as an array of states in which all transition dependencies run in one direction; we can do an iterative dynamic programming calculation through the model states starting with the last numbered end state M and ending in the root state 1. An example CM, corresponding to the input alignment of Figure 1, is shown in Figure 3.

As a convenient side effect of the construction procedure, it is guaranteed that the transitions from any state are to a *contiguous* set of child states, so the transitions for state v may be kept as an offset and a count. For example, in Figure 3, state 12 (an MP) connects to states 16, 17, 18, 19, 20, and 21. We can store this as an offset of 4 to the first connected state, and a total count of 6 connected states. We know that the offset is the distance to the next non-split state in the current node; we also know that the count is equal to the number of insert states in the current node, plus the number of split set states in the next node. These properties make establishing the connectivity of the CM trivial. Similarly, all the parents of any given state are also contiguously numbered, and can be determined analogously. We are also guaranteed that the states in a split set are numbered contiguously. This contiguity is exploited by the divide and conquer implementation.

Parameterization

Using the guide tree and the final CM, each individual sequence in the input multiple alignment can be converted unambiguously to a CM parse tree, as shown in Figure 4. Weighted counts for observed state transitions and singlet/pair emissions are then collected from these parse trees. These counts are converted to transition and emission probabilities, as maximum *a posteriori* estimates using mixture Dirichlet priors (Sjölander et al., 1996; Durbin et al., 1998; Nawrocki and Eddy, 2007).

Comparison to profile HMMs

The relationship between an SCFG and a covariance model is analogous to the relationship of hidden Markov models (HMMs) and profile HMMs for modeling multiple sequence alignments (Krogh et al., 1994; Durbin et al., 1998; Eddy, 1998). A comparison may be instructive to readers familiar with profile HMMs. A profile HMM is a repetitive HMM architecture that associates each consensus column of a multiple alignment with a single type of model node – a MATL node, in the above notation. Each node contains a "match", "delete", and "insert" HMM state – ML, IL, and D states, in the above notation. The profile HMM also has special begin and end states. Profile HMMs could therefore be thought of as a special case of CMs. An unstructured RNA multiple alignment would be modeled by a guide tree of all MATL nodes, and converted to an unbifurcated CM that would essentially be identical to a profile HMM. (The only difference is trivial; the CM root node includes a IR state, whereas the start node of a profile HMM does not.) All the other node types (especially MATP, MATR, and BIF) and state types (e.g. MP, MR, IR, and B) are SCFG augmentations necessary to extend profile HMMs to deal with RNA secondary structure.

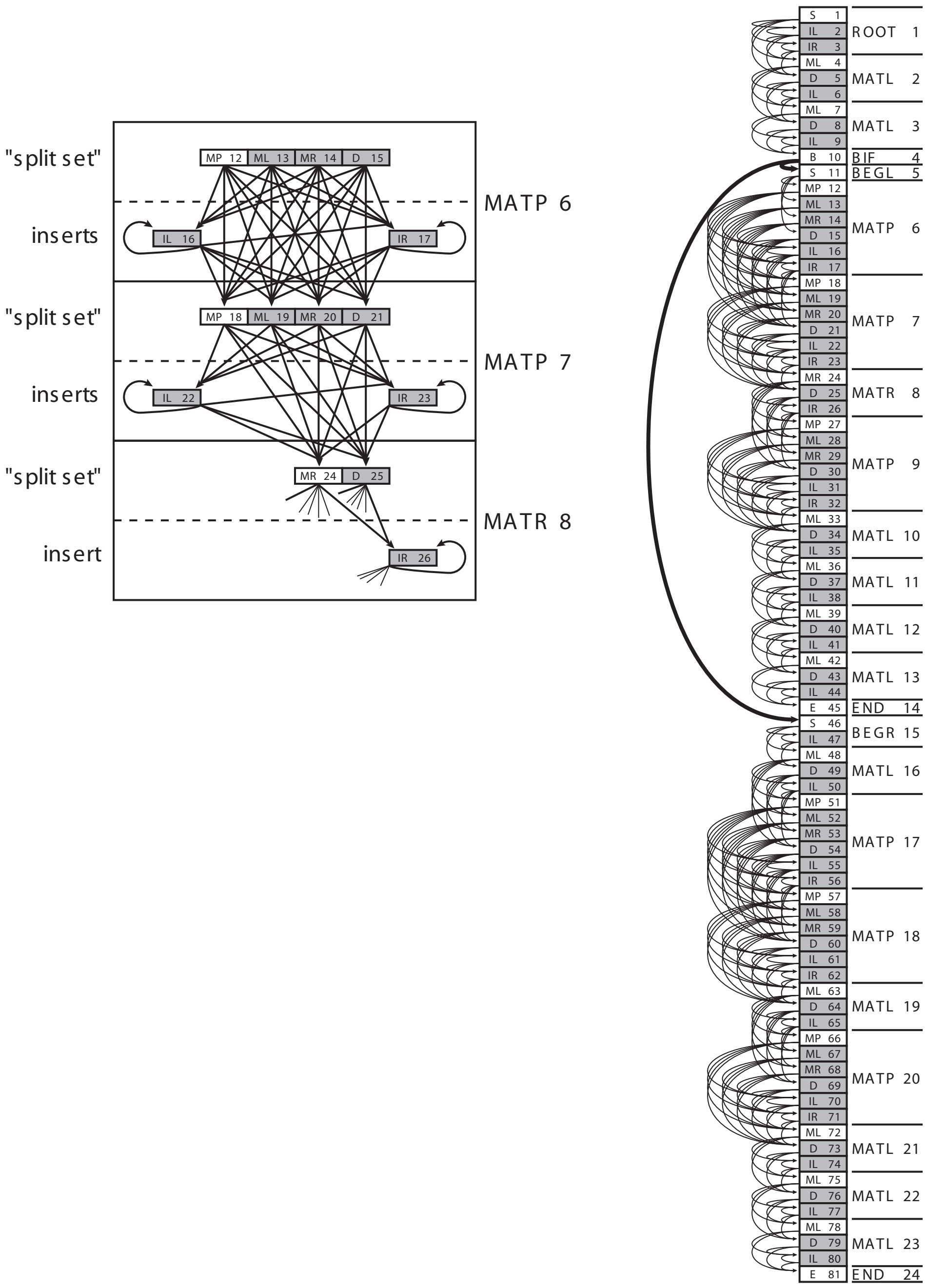

Figure 3: **A complete covariance model.** Right: the CM corresponding to the alignment in Figure 1. The model has 81 states (boxes, stacked in a vertical array). Each state is associated with one of the 24 nodes of the guide tree (text to the right of the state array). States corresponding to the consensus are in white. States responsible for insertions and deletions are gray. The transitions from bifurcation state B10 to start states S11 and S46 are in bold because they are special: they are an obligate (probability 1) bifurcation. All other transitions (thin arrows) are associated with transition probabilities. Emission probability distributions are not represented in the figure. Left: the states are also arranged according to the guide tree. A blow up of part of the model corresponding to nodes 6, 7, and 8 shows more clearly the logic of the connectivity of transition probabilities (see main text), and also shows why any parse tree must transit through one and only one state in each "split set".

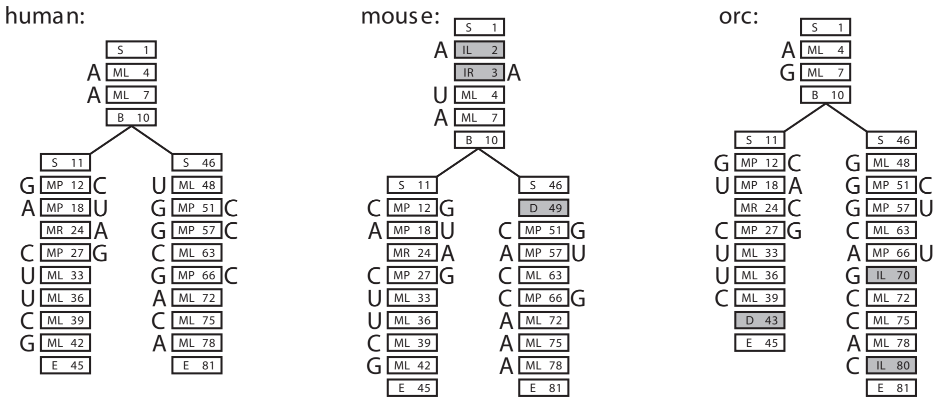

Figure 4: **Example parse trees.** Parse trees are shown for the three sequences/structures from Figure 1, given the CM in Figure 3. For each sequence, each residue must be associated with a state in the parse tree. (The sequences can be read off its parse tree by starting at the upper left and reading counterclockwise around the edge of parse tree.) Each parse tree corresponds directly to a secondary structure – base pairs are pairs of residues aligned to MP states. A collection of parse trees also corresponds to a multiple alignment, by aligning residues that are associated with the same state – for example, all three trees have a residue aligned to state ML4, so these three residues would be aligned together. Insertions and deletions relative to the consensus use nonconsensus states, shown in gray.

The `cmbuild` program, step by step

The `cmbuild` command line syntax is:

`> cmbuild <options> [cmfile] [alifile]`

where `[alifile]` is the name of the input alignment file, and `[cmfile]` is the name of the output CM file. What follows describes the steps that `cmbuild` goes through, and the most important options that can be chosen to affect its behavior.

Alignment input file

The input alignment file must be in Stockholm format, and it must have a consensus secondary structure annotation line (`#=GC SS_cons`).

The program is actually capable of reading many common multiple alignment formats (ClustalW, PHYLIP, GCG MSF, and others) but no other format currently supports consensus RNA secondary structure annotation. This may change in the future, either when other formats allow structure annotation, or when `cmbuild` is capable of inferring consensus structure from the alignment by automated comparative analysis, as the earlier COVE suite was capable of (Eddy and Durbin, 1994).

If the file does not exist, is not readable, or is not in a recognized format, the program exits with a “Alignment file doesn’t exist or is not readable” error. If the file does not have consensus secondary structure annotation, the program exits with a “no consensus structure annotation” error. This includes all non-Stockholm alignment files.

▷ ***Why does `cmbuild` have a `--informat` option, if it only accepts Stockholm?*** *If you don’t specify `--informat`, the software has to autodetect the file format. Autodetection of file formats doesn’t work in certain advanced/nonstandard cases, for instance if you’re reading the alignment from standard input instead of from a file. The `--informat` allows you to override autodetection; e.g. `cat my.sto | cmbuild --informat Stockholm my.cm -` is an example of reading the alignment from piped standard input.*

Parsing secondary structure annotation

The structure annotation line only needs to indicate which columns are base paired to which. It does not have to be in full WUSS notation. Even if it is, the details of the notation are largely ignored. Nested pairs of <>, (), [], or symbols

are interpreted as base paired columns. All other columns marked with the symbols `:,_-.` are interpreted as single stranded columns.

A simple minimal annotation is therefore to use <> symbols to mark base pairs and `.` for single stranded columns.

If a secondary structure annotation line is in WUSS notation and it contains valid pseudoknot annotation (e.g. additional non-nested stems marked with AAA,aaa or BBB,bbb, etc.), this annotation is ignored because Infernal cannot handle pseudoknots. Internally, these columns are treated as if they were marked with `.` symbols.

> ▷ ***How should I choose to annotate pseudoknots?*** *Infernal can only deal with nested base pairs. If there is a pseudoknot, you have to make a choice of which stem to annotate as normal nested structure (thus including it in the model) and which stem to call additional "pseudoknotted" structure (thus ignoring it in the model). For example, for a simple two-stem pseudoknot, should you annotate it as* `AAAA.<<<<aaaa....>>>>`*, or* `<<<<.AAAA>>>>....aaaa`*? From an RNA structure viewpoint, which stem I label as the pseudoknotted one is an arbitrary choice; but since one of the stems in the pseudoknot will have to be modeled as a single stranded region by Infernal, the choice makes a slight difference in the performance of your model. You want your model to capture as much information content as possible. Thus, since the information content of the model is a sum of the sequence conservation plus the additional information contributed by pairwise correlations in base-paired positions, you should tend to annotate the shorter stem as the "pseudoknot" (modeling as many base pairs as possible), and you should also annotate the stem with the more conserved primary sequence as the "pseudoknot" (if one stem is more conserved at the sequence level, you won't lose as much by modeling that one as primary sequence consensus only).*

If (aside from any ignored pseudoknot annotation) the structure annotation line contains characters other than `<>()[]:_-,.` then those characters are ignored (treated as `.`) and a warning is printed.

If, after this "data cleaning", the structure annotation is inconsistent with a secondary structure (for example, if the number of < and > characters isn't the same), then the program exits with a "failed to parse consensus structure annotation" error.

Sequence weighting

By default, the input sequences are weighted in two ways to compensate for biased sampling (phylogenetic correlations). Relative sequence weights are calculated by the Henikoff position-based method. (Henikoff and Henikoff, 1994). (The `--wpb` option forces position-based weights, but is redundant since that's the default.) To turn relative weighting off (e.g. set all weights to 1.0), use the `--wnone` option.

Some alignment file formats allow relative sequence weights to be given in the file. This includes Stockholm format, which has `#=GS WT` weight annotations. Normally `cmbuild` ignores any such input weights. The `--wgiven` option tells `cmbuild` to use them. This lets you set the weights with any external procedure you like; for example, the `esl-weight` utility program in Easel[2] implements some common weighting algorithms, including the Gerstein/Sonnhammer/Chothia weighting scheme (Gerstein et al., 1994).

Absolute weights (the "effective sequence number") is calculate by "entropy weighting" (Karplus et al., 1998). This sets the balance between the prior and the data, and affects the information content of the model. Entropy weighting reduces the effective sequence number (the total sum of the weights) and increases the entropy (degrading the information content) of the model until a threshold is reached. The default entropy is 1.41 bits per position (roughly 0.59 bits of information, relative to uniform base composition). This threshold can be changed with the `--ere` <x> option. Entropy weighting may be turned off entirely with the `--enone` option.

Architecture construction

The CM architecture is now constructed from your input alignment and your secondary structure annotation, as described in the previous section.

The program needs to determine which columns are consensus (match) columns, and which are insert columns. (Remember that although WUSS notation allows insertions to be annotated in the secondary structure line, `cmbuild` is only paying attention to annotated base pairs.) By default, it does this by a simple rule based on the frequency of residues (non-gaps) in a column. If the frequency of residues is lower than a threshold, the column is considered to be an insertion. Importantly though this frequency is determined using the relative weights from the sequence weighting step, instead of absolute gaps (e.g. a residue in a sequence with weight 0.8 will count as 0.8 residues).

[2] This program will be in `infernal/easel/miniapps/` after building Infernal.

The threshold defaults to 0.5. It can be changed to another number <x> (from 0 to 1.0) by the `--symfrac <x>` option. The lower the number, the more columns are included in the model. At `--symfrac 0.0`, all the columns are considered to be part of the consensus. At `--symfrac 1.0`, only columns with no gaps are.

You can also manually specify which columns are consensus versus insert by including reference coordinate annotation (e.g. a `#=GC RF` line, in Stockholm format) and using the `--hand` option. There's an example of this in the tutorial. Any columns marked by non-gap symbols become consensus columns. (The simplest thing to do is mark consensus columns with x's, and insert columns with `.`'s. Remember that spaces aren't allowed in alignments in Stockholm format.) If you set the `--hand` option but your file doesn't have reference coordinate annotation, the program exits with an error.

Parameterization

Weighted observed emission and transition counts are then collected from the alignment data. These count vectors c are then converted to estimated probabilities p using mixture Dirichlet priors (Sjölander et al., 1996; Durbin et al., 1998; Nawrocki and Eddy, 2007). You can provide your own prior as a file, using the `--prior <f>` option.

As an exception, insert state emission probabilities are not learned from the counts from implicit parse trees of sequences in the input alignment, instead they are all set to 0.25 for each of the four RNA nucleotides. Another exception is made for transition counts in ROOT_IL and ROOT_IR states from the implicit parsetrees. Any transition counts in these states are *ignored* by the construction procedure – they are set to zero before the transition probability parameters for these states are determined.

Naming the model

Each CM gets a name. Stockholm format allows the alignment to have a name, provided in the `#=GF ID` tag. If this name is provided, it is used as the CM name.

Stockholm format allows more than one alignment per file, and `cmbuild` supports this: CM files can contain more than one model, and if you say e.g. `cmbuild Rfam.cm Rfam.sto` where `Rfam.sto` contains a whole database of alignments, `cmbuild` will create a database of CMs in the `Rfam.cm` file, one per alignment.

If a name is not provided in the Stockholm `#=GF ID` annotation, the name given to each CM is the input filename. This will not work if the alignment file has more than one alignment. In that case, you must include names for each alignment.

If the alignment file only has 1 alignment in it, you can override the automatic naming conventions and provide your own name with the `-n <s>` option, where <s> is any string.

Saving the model

The model is now saved to a file, according to the filename specified on the command line. By default, a new file is created, and the model is saved in a portable ASCII text format. This format is described in section 9 of this guide.

If the cmfile already exists, the program exits with an error. The `-F` option causes the new model to overwrite an existing cmfile.

6 Tabular output formats

Target hits tables

The `--tblout` output option in `cmsearch` and `cmscan` produces *target hits tables*. There are two different formats of target hits table, which are both described below. By default, both `cmsearch` and `cmscan` produce the target hits table in *format 1*. Format 1 is the only format that was used by Infernal versions 1.1rc1 through 1.1.1. As of version 1.1.2, with `cmscan`, the `--fmt 2` option can be used in combination with `--tblout` to produce a target hits table in the alternative *format 2*. Both formats 1 and 2 target hits table consist of one line for each different query/target comparison that met the reporting thresholds, ranked by decreasing statistical significance (increasing E-value).

Target hits table format 1

In the format 1 table, each line consists of **18 space-delimited fields** followed by a free text target sequence description, as follows:[1]

(1) target name: The name of the target sequence or profile.

(2) accession: The accession of the target sequence or profile, or '-' if none.

(3) query name: The name of the query sequence or profile.

(4) accession: The accession of the query sequence or profile, or '-' if none.

(5) mdl (model): Which type of model was used to compute the final score. Either 'cm' or 'hmm'. A CM is used to compute the final hit scores unless the model has zero basepairs or the `--hmmonly` option is used, in which case a HMM will be used.

(6) mdl from (model coord): The start of the alignment of this hit with respect to the profile (CM or HMM), numbered 1..N for a profile of N consensus positions.

(7) mdl to (model coord): The end of the alignment of this hit with respect to the profile (CM or HMM), numbered 1..N for a profile of N consensus positions.

(8) seq from (ali coord): The start of the alignment of this hit with respect to the sequence, numbered 1..L for a sequence of L residues.

(9) seq to (ali coord): The end of the alignment of this hit with respect to the sequence, numbered 1..L for a sequence of L residues.

(10) strand: The strand on which the hit occurs on the sequence. '+' if the hit is on the top (Watson) strand, '-' if the hit is on the bottom (Crick) strand. If on the top strand, the "seq from" value will be less than or equal to the "seq to" value, else it will be greater than or equal to it.

(11) trunc: Indicates if this is predicted to be a truncated CM hit or not. This will be "no" if it is a CM hit that is not predicted to be truncated by the end of the sequence, "5' " or "3' " if the hit is predicted to have one or more 5' or 3' residues missing due to a artificial truncation of the sequence, or "5'&3'" if the hit is predicted to have one or more 5' residues missing and one or more 3' residues missing. If the hit is an HMM hit, this will always be '-'.

(12) pass: Indicates what "pass" of the pipeline the hit was detected on. This is probably only useful for testing and debugging. Non-truncated hits are found on the first pass, truncated hits are found on successive passes.

(13) gc: Fraction of G and C nucleotides in the hit.

(14) bias: The biased-composition correction: the bit score difference contributed by the null3 model for CM hits, or the null2 model for HMM hits. High bias scores may be a red flag for a false positive. It is difficult to correct for all possible ways in which a nonrandom but nonhomologous biological sequences can appear to be similar, such as short-period tandem repeats, so there are cases where the bias correction is not strong enough (creating false positives).

(15) score: The score (in bits) for this target/query comparison. It includes the biased-composition correction (the "null3" model for CM hits, or the "null2" model for HMM hits).

---

[1] The `tblout` format is deliberately space-delimited (rather than tab-delimited) and justified into aligned columns, so these files are suitable both for automated parsing and for human examination. Tab-delimited data files are difficult for humans to examine and spot check. For this reason, we think tab-delimited files are a minor evil in the world. Although we occasionally receive shrieks of outrage about this, we stubbornly feel that space-delimited files are just as trivial to parse as tab-delimited files.

(16) E-value: The expectation value (statistical significance) of the target. This is a *per query* E-value; i.e. calculated as the expected number of false positives achieving this comparison's score for a *single* query against the search space Z. For `cmsearch` Z is defined as the total number of nucleotides in the target dataset multiplied by 2 because both strands are searched. For `cmscan` Z is the total number of nucleotides in the query sequence multiplied by 2 because both strands are searched and multiplied by the number of models in the target database. If you search with multiple queries and if you want to control the *overall* false positive rate of that search rather than the false positive rate per query, you will want to multiply this per-query E-value by how many queries you're doing.

(17) inc: Indicates whether or not this hit achieves the inclusion threshold: '!' if it does, '?' if it does not (and rather only achieves the reporting threshold). By default, the inclusion threshold is an E-value of 0.01 and the reporting threshold is an E-value of 10.0, but these can be changed with command line options as described in the manual pages.

(18) description of target: The remainder of the line is the target's description line, as free text.

Target hits table format 2

Format 2 includes all 18 of the fields from format 1 in the same order, plus 9 additional fields that are interspersed between some of the 18 from format 1, as follows:

(Before field 1 of format 1) idx: The index of the hit in the list. The first hit has index '1', the second has index '2', the Nth hit has index 'N'.

(Before field 5 of format 1) clan name: The name of the clan the model for this hit belongs to, or - if the model does not belong to a clan. A clan is a group of related models. For example, Rfam groups three LSU rRNA models (LSU_rRNA_archaea, LSU_rRNA_bacteria, and LSU_rRNA_eukarya) into the same clan. The value in this field will always be - unless the `--clanin <f>` option was used with `cmscan` to specify clan/model relationships in the input file `<f>`. See section 9 for a description of the format of the input file used with `--clanin`.

The following seven fields all occur in format 2 between fields 17 ('inc:') and 18 ('description of target') from format 1.

olp: A single character indicating the overlap status of this hit. Here, two hits are deemed to *overlap* if they share at least one nucleotide on the same strand of the same sequence. There are three possible values in this field: $*$, ^ and =. $*$ indicates this hit does not overlap with any other reported hits. ^ indicates that this hit does overlap with at least one other hit, but none of the hits that overlap with it have a higher score (occur above it in the hit list). = indicates that this hit does overlap with at least one other hit that has a higher score (occurs above it in the hit list). If the `--oclan` option was enabled, the definition of *overlap* for the designations of the three characters $*$, ^ and = described above changes to: two hits are deemed to *overlap* if they share at least one nucleotide on the same strand of the same sequence and they are to models that are in the same clan. That is, only overlaps between hits to models that are in the same clan are counted, all other overlaps are ignored and not annotated. Infernal will never report two overlapping hits to the same model.

anyidx: For hits that have = in the "olp" field, this is the index of the best scoring hit that overlaps with this hit. For hits with either $*$ or ^ in the "olp" field, this field will always be -.

anyfrct1: For hits that have = in the "olp" field, this is the fraction of the length of this hit that overlaps with the best scoring overlapping hit (the hit index given in the "anyidx" field), to 4 significant digits. For hits with - in the "anyidx" field, this field will always be -.

anyfrct2: For hits that have = in the "olp" field, this is the fraction of the length of the best scoring overlapping hit (the hit index given in the "anyidx" field) that overlaps with this hit, to 4 significant digits. For hits with - in the "anyidx" field, this field will always be -.

winidx: For hits that have = in the "olp" field, this is either **"** or the index of the best scoring hit that overlaps with this hit that is marked as ^ in the "olp" field. If the value is **"** it means that the best scoring hit that overlaps with this hit that is marked as ^ in the "olp" field is already listed in the "anyidx" field, which is usually the case. For hits with either $*$ or ^ in the "olp" field, this field will always be -.

winfrct1: For hits that have neither - nor **"** in the "winidx" field, this is the fraction of the length of this hit that overlaps with the best scoring overlapping hit marked with ^ in the "olp" field (the hit index given in the "winidx" field), to 4 significant digits. For hits with either $*$ or ^ in the "olp" field, this field will always be -. For hits with - in the "winidx" field, this field will always be -. For hits with **"** in the "winidx" field, this field will always be **"**.

winfrct2: For hits that have neither - nor " in the "winidx" field, this is the fraction of the length of the best scoring overlapping hit marked with ^ in the "olp" field (the hit index given in the "winidx" field) that overlaps with this hit, to 4 significant digits. For hits with either * or ^ in the "olp" field, this field will always be -. For hits with - in the "winidx" field, this field will always be -. For hits with " in the "winidx" field, this field will always be ".

The tables are columnated neatly for human readability, but do not write parsers that rely on this columnation; rely on space-delimited fields. The pretty columnation assumes fixed maximum widths for each field. If a field exceeds its allotted width, it will still be fully represented and space-delimited, but the columnation will be disrupted on the rest of the row.

Note the use of target and query columns. A program like `cmsearch` searches a query profile against a target sequence database. In an `cmsearch` tblout file, the sequence (target) name is first, and the profile (query) name is second. A program like `cmscan`, on the other hand, searches a query sequence against a target profile database. In a `cmscan` tblout file, the profile name is first, and the sequence name is second. You might say, hey, wouldn't it be more consistent to put the profile name first and the sequence name second (or vice versa), so `cmsearch` and `cmscan` tblout files were identical? Well, they still wouldn't be identical, because the target database size used for E-value calculations is different (total number of target nucleotides for `cmsearch`, number of target profiles times target sequence length for `cmscan`), and it's good not to forget this.

If some of the descriptions of these fields don't make sense to you, it may help to go through the tutorial in section 3 and read section 4 of the manual.

7 Some other topics

How do I cite Infernal?

If you'd like to cite a paper, please cite the Infernal 1.1 application note in *Bioinformatics*:

Infernal 1.1: 100-fold faster RNA homology searches. EP Nawrocki and SR Eddy. Bioinformatics, 29:2933-2935, 2013.

The most appropriate citation is to the web site, `http://eddylab.org/infernal/`. You should also cite what version of the software you used. We archive all old versions, so anyone should be able to obtain the version you used, when exact reproducibility of an analysis is an issue.

The version number is in the header of most output files. To see it quickly, do something like `cmscan -h` to get a help page, and the header will say:

```
# cmscan :: search sequence(s) against a CM database
# INFERNAL 1.1.2 (June 2016)
# Copyright (C) 2016 Howard Hughes Medical Institute.
# Freely distributed under a BSD open source license.
# - - - - - - - - - - - - - - - - - - - - - - - - - - - - - - - - - - - - -
```

So (from the second line there) this is from Infernal 1.1.2.

How do I report a bug?

Email us, at `sean@eddylab.org`.

Before we can see what needs fixing, we almost always need to reproduce a bug on one of our machines. This means we want to have a small, reproducible test case that shows us the failure you're seeing. So if you're reporting a bug, please send us:

- A brief description of what went wrong.
- The command line(s) that reproduce the problem.
- Copies of any files we need to run those command lines.
- Information about what kind of hardware you're on, what operating system, and (if you compiled the software yourself rather than running precompiled binaries), what compiler and version you used, with what configuration arguments.

Depending on how glaring the bug is, we may not need all this information, but any work you can put into giving us a clean reproducible test case doesn't hurt and often helps.

The information about hardware, operating system, and compiler is important. Bugs are frequently specific to particular configurations of hardware/OS/compiler. We have a wide variety of systems available for trying to reproduce bugs, and we'll try to match your system as closely as we can.

If you first see a problem on some huge compute (like running a zillion query sequence over a huge profile database), it will really, really help us if you spend a bit of time yourself trying to isolate whether the problem really only manifests itself on that huge compute, or if you can isolate a smaller test case for us. The ideal bug report (for us) gives us everything we need to reproduce your problem in one email with at most some small attachments.

Remember, we're not a company with dedicated support staff – we're a small lab of busy researchers like you. Somebody here is going to drop what they're doing to try to help you out. Try to save us some time, and we're more likely to stay in our usual good mood.

If we're in our usual good mood, we'll reply quickly. We'll probably tell you we fixed the bug in our development code, and that the fix will appear in the next Infernal release. This of course doesn't help you much, since nobody knows when the next Infernal release is going to be. So if possible, we'll usually try to describe a workaround for the bug.

If the code fix is small, we might also tell you how to patch and recompile the code yourself. You may or may not want to do this.

There are currently not enough open bugs to justify having a formal on-line bug tracking system. We have a bugtracking system, but it's internal.

Input files

Reading from a stdin pipe using - (dash) as a filename argument

Generally, Infernal programs read their sequence and/or profile input from files. Unix power users often find it convenient to string an incantation of commands together with pipes (indeed, such wizardly incantations are a point of pride). For example, you might extract a subset of query sequences from a larger file using a one-liner combination of scripting commands (perl, awk, whatever). To facilitate the use of Infernal programs in such incantations, you can almost always use an argument of '-' (dash) in place of a filename, and the program will take its input from a standard input pipe instead of opening a file.[1]

For example, the following three commands are entirely equivalent, and give essentially identical output:

```
> cmalign tRNA5.cm mrum-tRNAs10.fa
> cat tRNA5.cm | ../src/cmalign - mrum-tRNAs10.fa
> cat mrum-tRNAs10.fa | ../src/cmalign tRNA5.cm -
```

Most Easel "miniapp" programs share the same ability of pipe-reading.

Because the programs for CM fetching (`cmfetch`) and sequence fetching (`esl-sfetch`) can fetch any number of profiles or (sub)sequences by names/accessions given in a list, *and* these programs can also read these lists from a stdin pipe, you can craft incantations that generate subsets of queries or targets on the fly. For example, you can extract and align all hits found by `cmsearch` with an E-value below the inclusion threshold as follows (using the \character twice below to split up the final command onto three lines):

```
> cmsearch --tblout tRNA5.mrum-genome.tbl tRNA5.cm mrum-genome.fa
> esl-sfetch --index mrum-genome.fa
> cat tRNA5.mrum-genome.tbl | grep -v ^# | grep ! \
> | awk '{ printf(``%s/%s-%s %s %s %s\n'', $1, $8, $9, $8, $9, $1); }' \
> | esl-sfetch -Cf mrum-genome.fa - | ../src/cmalign tRNA5.cm -
```

The first command performed the search using the CM file `tRNA5.c.cm` and sequence file `mrum-genome.fa` (these were used in the tutorial), and saved tabular output to `tRNA5.mrum-genome.tbl`. The second command indexed the genome file to prepare it for fast (sub)sequence retrieval. In the third command we've extracted only those lines from `tRNA5.mrum-genome.tbl` that do not begin with a `#` (these are comment lines) and also include a `!` (these are hits that have E-values below the inclusion threshold) using the first two `grep` commands. This output was then sent through `awk` to reformat the tabular output to the "GDF" format that `esl-sfetch` expects: `<newname> <from> <to> <source seqname>`. These lines are then piped into `esl-sfetch` (using the '-' argument) to retrieve each hit (only the subsequence that comprises each hit – not the full target sequence). `esl-sfetch` will output a FASTA file, which is finally being piped into `cmalign`, again using the '-' argument. The output that is actually printed to the screen will be a multiple alignment of all the included tRNA hits.

You can do similar commands piping subsets of CMs. Supposing you have a copy of Rfam in Rfam.cm:

```
> cmfetch --index Rfam.cm
> cat myqueries.list | cmfetch -f Rfam.cm - | cmsearch - mrum-genome.fa
```

This takes a list of query CM names/accessions in `myqueries.list`, fetches them one by one from Rfam, and does an cmsearch with each of them against the sequence file `mrum-genome.fa`. As above, the `cat myqueries.list` part can be replaced by any suitable incantation that generates a list of profile names/accessions.

There are three kinds of cases where using '-' is restricted or doesn't work. A fairly obvious restriction is that you can only use one '-' per command; you can't do a `cmalign - -` that tries to read both a CM and sequences through the same stdin pipe. Second, another case is when an input file must be obligately associated with additional, separately generated auxiliary files, so reading data from a single stream using '-' doesn't work because the auxiliary files aren't present (in this case, using '-' will be prohibited by the program). An example is `cmscan`, which needs its `<cmfile>` argument to be associated with four auxiliary files named `<cmfile>.i1{mifp}` that `cmpress` creates, so `cmscan` does not permit a '-' for its `<cmfile>` argument. Finally, when a command would require multiple passes over an input file the command will generally abort after the first pass if you are trying to read that file through a standard input pipe (pipes are nonrewindable in general; a few Easel programs will buffer input streams to make multiple passes possible, but this is not usually the case). An important example is trying to search a database that is streamed into `cmsearch`.

[1] An important exception is the use of '-' in place of the target sequence file in `cmsearch`. This is not allowed because `cmsearch` first quickly reads the target sequence file to determine its size (it needs to know this to know how to set filter thresholds), then rewinds it and starts to process it. There's a couple of additional cases where stdin piping won't work described later in this section.

This is not allowed because `cmsearch` first reads the entire sequence file to determine its size (which dictates the filter thresholds that will be used for the search), then needs to rewind the file before beginning the search.

In general, Infernal, HMMER and Easel programs document in their man page whether (and which) command line arguments can be replaced by '-'. You can always check by trial and error, too. The worst that can happen is a "Failed to open file -" error message, if the program can't read from pipes.

8 Manual pages

`cmalign` - align sequences to a covariance model

Synopsis

cmalign *[options]* <*cmfile*> <*seqfile*>

Description

cmalign aligns the RNA sequences in <*seqfile*> to the covariance model (CM) in <*cmfile*>. The new alignment is output to *stdout* in Stockholm format, but can be redirected to a file <*f*> with the **-o** <*f*> option.

Either <*cmfile*> or <*seqfile*> (but not both) may be '-' (dash), which means reading this input from *stdin* rather than a file.

The sequence file <*seqfile*> must be in FASTA or Genbank format.

cmalign uses an HMM banding technique to accelerate alignment by default as described below for the **--hbanded** option. HMM banding can be turned off with the **--nonbanded** option.

By default, **cmalign** computes the alignment with maximum expected accuracy that is consistent with constraints (bands) derived from an HMM, using a banded version of the Durbin/Holmes optimal accuracy algorithm. This behavior can be changed with the **--cyk** or **--sample** options.

cmalign takes special care to correctly align truncated sequences, where some nucleotides from the beginning (5') and/or end (3') of the actual full length biological sequence are not present in the input sequence (see DL Kolbe and SR Eddy, Bioinformatics, 25:1236-1243, 2009). This behavior is on by default, but can be turned off with **--notrunc.** In previous versions of **cmalign** the **--sub** option was required to appropriately handle truncated sequences. The **--sub** option is still available in this version, but the new default method for handling truncated sequences should be as good or superior to the sub method in nearly all cases.

The **--mapali** <*s*> option allows inclusion of the fixed training alignment used to build the CM from file <*s*> within the output alignment of **cmalign.**

It is possible to merge two or more alignments created by the same CM using the Easel miniapp **esl-alimerge** (included in the easel/miniapps/ subdirectory of Infernal). Previous versions of **cmalign** included options to merge alignments but they were deprecated upon development of **esl-alimerge,** which is significantly more memory efficient.

By default, **cmalign** will output the alignment to stdout. The alignment can be redirected to an output file <*f*> with the **-o** <*f*> option. With **-o,** information on each aligned sequence, including score and model alignment boundaries will be printed to stdout (more on this below).

The output alignment will be in Stockholm format by default. This can be changed to Pfam, aligned FASTA (AFA), A2M, Clustal, or Phylip format using the **--outformat** <*s*> option, where <*s*> is the name of the desired format. As a special case, if the output alignment is large (more than 10,000 sequences or more than 10,000,000 total nucleotides) than the output format will be Pfam format, with each sequence appearing on a single line, for reasons of memory efficiency. For alignments larger than this, using **--ileaved** will force interleaved Stockholm format, but the user should be aware that this may require a lot of memory. **--ileaved** will only work for alignments up to 100,000 sequences or 100,000,000 total nucleotides.

If the output alignment format is Stockholm or Pfam, the output alignment will be annotated with posterior probabilities which estimate the confidence level of each aligned nucleotide. This annotation appears as lines beginning with "#=GR <seq name> PP", one per sequence, each immediately below the corresponding aligned sequence "<seq name>". Characters in PP lines have 12 possible values: "0-9", "*", or ".". If ".", the position corresponds to a gap in the sequence. A value of "0" indicates a posterior probability of between 0.0 and 0.05, "1" indicates between 0.05 and 0.15, "2" indicates between 0.15 and 0.25 and so on up to "9" which indicates between 0.85 and 0.95. A value of "*" indicates a posterior probability of between 0.95 and 1.0. Higher posterior probabilities correspond to greater confidence that the aligned nucleotide belongs where it appears in the alignment. With **--nonbanded,** the calculation of the posterior

probabilities considers all possible alignments of the target sequence to the CM. Without **--nonbanded** (i.e. in default mode), the calculation considers only possible alignments within the HMM bands. Further, the posterior probabilities are conditional on the truncation mode of the alignment. For example, if the sequence alignment is truncated 5', a PP value of "9" indicates between 0.85 and 0.95 of all 5' truncated alignments include the given nucleotide at the given position. The posterior annotation can be turned off with the **--noprob** option. If **--small** is enabled, posterior annotation must also be turned off using **--noprob.**

The tabular output that is printed to stdout if the **-o** option is used includes one line per sequence and twelve fields per line: "idx": the index of the sequence in the input file, "seq name": the sequence name; "length": the length of the sequence; "cm from" and "cm to": the model start and end positions of the alignment; "trunc": "no" if the sequence is not truncated, "5'" if the beginning of the sequence truncated 5', "3'" if the end of the sequence is truncated, and "5'&3'" if both the beginning and the end are truncated; "bit sc": the bit score of the alignment, "avg pp" the average posterior probability of all aligned nucleotides in the alignment; "band calc", "alignment" and "total": the time in seconds required for calculating HMM bands, computing the alignment, and complete processing of the sequence, respectively; "mem (Mb)": the size in Mb of all dynamic programming matrices required for aligning the sequence. This tabular data can be saved to file <*f*> with the **--sfile** <*f*> option.

Options

-h Help; print a brief reminder of command line usage and available options.

-o <*f*> Save the alignment in Stockholm format to a file <*f*>*.* The default is to write it to standard output.

-g Configure the model for global alignment of the query model to the target sequences. By default, the model is configured for local alignment. Local alignments can contain large insertions and deletions called "local ends" in the structure to be penalized differently than normal indels. These are annotated as " " columns in the RF line of the output alignment. The **-g** option can be used to disallow these local ends. The **-g** option is required if the **--sub** option is also used.

Options for Controlling the Alignment Algorithm

--optacc Align sequences using the Durbin/Holmes optimal accuracy algorithm. This is the default. The optimal accuracy alignment will be constrained by HMM bands for acceleration unless the **--nonbanded** option is enabled. The optimal accuracy algorithm determines the alignment that maximizes the posterior probabilities of the aligned nucleotides within it. The posterior probabilites are determined using (possibly HMM banded) variants of the Inside and Outside algorithms.

--cyk Do not use the Durbin/Holmes optimal accuracy alignment to align the sequences, instead use the CYK algorithm which determines the optimally scoring (maximum likelihood) alignment of the sequence to the model, given the HMM bands (unless **--nonbanded** is also enabled).

--sample Sample an alignment from the posterior distribution of alignments. The posterior distribution is determined using an HMM banded (unless **--nonbanded)** variant of the Inside algorithm.

--seed <*n*> Seed the random number generator with <*n*>*,* an integer >= 0. This option can only be used in combination with **--sample.** If <*n*> is nonzero, stochastic sampling of alignments will be reproducible; the same command will give the same results. If <*n*> is 0, the random number generator is seeded arbitrarily, and stochastic samplings may vary from run to run of the same command. The default seed is 181.

--notrunc Turn off truncated alignment algorithms. All sequences in the input file will be assumed to be full length, unless **--sub** is also used, in which case the program can still handle truncated sequences but will use an alternative strategy for their alignment.

--sub Turn on the sub model construction and alignment procedure. For each sequence, an HMM is first used to predict the model start and end consensus columns, and a new sub CM is constructed that only models consensus columns from start to end. The sequence is then aligned to this sub CM. Sub alignment is an older method than the default one for aligning sequences that are possibly truncated. By default, **cmalign** uses special DP algorithms to handle truncated sequences which should be more accurate than the sub method in most cases. **--sub** is still included as an option mainly for testing against this default truncated sequence handling. This ”sub CM” procedure is not the same as the ”sub CMs” described by Weinberg and Ruzzo.

Options for Controlling Speed and Memory Requirements

--hbanded This option is turned on by default. Accelerate alignment by pruning away regions of the CM DP matrix that are deemed negligible by an HMM. First, each sequence is scored with a CM plan 9 HMM derived from the CM using the Forward and Backward HMM algorithms to calculate posterior probabilities that each nucleotide aligns to each state of the HMM. These posterior probabilities are used to derive constraints (bands) on the CM DP matrix. Finally, the target sequence is aligned to the CM using the banded DP matrix, during which cells outside the bands are ignored. Usually most of the full DP matrix lies outside the bands (often more than 95%), making this technique faster because fewer DP calculations are required, and more memory efficient because only cells within the bands need be allocated. Importantly, HMM banding sacrifices the guarantee of determining the optimally accurarte or optimal alignment, which will be missed if it lies outside the bands. The tau paramater is the amount of probability mass considered negligible during HMM band calculation; lower values of tau yield greater speedups but also a greater chance of missing the optimal alignment. The default tau is 1E-7, determined empirically as a good tradeoff between sensitivity and speed, though this value can be changed with the **--tau** ” <**x**>” option. The level of acceleration increases with both the length and primary sequence conservation level of the family. For example, with the default tau of 1E-7, tRNA models (low primary sequence conservation with length of about 75 nucleotides) show about 10X acceleration, and SSU bacterial rRNA models (high primary sequence conservation with length of about 1500 nucleotides) show about 700X. HMM banding can be turned off with the **--nonbanded** option.

--tau <*x*> Set the tail loss probability used during HMM band calculation to <*x*>. This is the amount of probability mass within the HMM posterior probabilities that is considered negligible. The default value is 1E-7. In general, higher values will result in greater acceleration, but increase the chance of missing the optimal alignment due to the HMM bands.

--mxsize <*x*> Set the maximum allowable total DP matrix size to <*x*> megabytes. By default this size is 1028 Mb. This should be large enough for the vast majority of alignments, however if it is not **cmalign** will attempt to iteratively tighten the HMM bands it uses to constrain the alignment by raising the tau parameter and recalculating the bands until the total matrix size needed falls below <*x*> megabytes or the maximum allowable tau value (0.05 by default, but changeable with **--maxtau)** is reached. At each iteration of band tightening, tau is multiplied by a 2.0. The band tightening strategy can be turned off with the **--fixedtau** option. If the maximum tau is reached and the required matrix size still exceeds <*x*> or if HMM banding is not being used and the required matrix size exceeds <*x*> then **cmalign** will exit prematurely and report an error message that the matrix exceeded its maximum allowable size. In this case, the **--mxsize** can be used to raise the size limit or the maximum tau can be raised with **--maxtau.** The limit will commonly be exceeded when the **--nonbanded** option is used without the **--small** option, but can still occur when **--nonbanded** is not used. Note that if **cmalign** is being run in <*n*> multiple threads on a multicore machine then each thread may have an allocated matrix of up to size <*x*> Mb at any given time.

--fixedtau Turn off the HMM band tightening strategy described in the explanation of the **--mxsize** option above.

--maxtau <*x*> Set the maximum allowed value for tau during band tightening, described in the explanation of **--mxsize** above, to <*x*>. By default this value is 0.05.

--nonbanded Turns off HMM banding. The returned alignment is guaranteed to be the globally optimally accurate one (by default) or the globally optimally scoring one (if **--cyk** is enabled). The **--small** option is recommended in combination with this option, because standard alignment without HMM banding requires a lot of memory (see **--small**).

--small Use the divide and conquer CYK alignment algorithm described in SR Eddy, BMC Bioinformatics 3:18, 2002. The **--nonbanded** option must be used in combination with this options. Also, it is recommended whenever **--nonbanded** is used that **--small** is also used because standard CM alignment without HMM banding requires a lot of memory, especially for large RNAs. **--small** allows CM alignment within practical memory limits, reducing the memory required for alignment LSU rRNA, the largest known RNAs, from 150 Gb to less than 300 Mb. This option can only be used in combination with **--nonbanded, --notrunc,** and **--cyk.**

Optional Output Files

--sfile <*f*> Dump per-sequence alignment score and timig information to file <*f*>*.* The format of this file is described above (it's the same data in the same format as the tabular stdout output when the **-o** option is used).

--tfile <*f*> Dump tabular sequence tracebacks for each individual sequence to a file <*f*>*.* Primarily useful for debugging.

--ifile <*f*> Dump per-sequence insert information to file <*f*>*.* The format of the file is described by "#"-prefixed comment lines included at the top of the file <*f*>*.* The insert information is valid even when the **--matchonly** option is used.

--elfile <*f*> Dump per-sequence EL state (local end) insert information to file <*f*>*.* The format of the file is described by "#"-prefixed comment lines included at the top of the file <*f*>*.* The EL insert information is valid even when the **--matchonly** option is used.

Other Options

--mapali <*f*> Reads the alignment from file <*f*> used to build the model aligns it as a single object to the CM; e.g. the alignment in <*f*> is held fixed. This allows you to align sequences to a model with **cmalign** and view them in the context of an existing trusted multiple alignment. <*f*> must be the alignment file that the CM was built from. The program verifies that the checksum of the file matches that of the file used to construct the CM. A similar option to this one was called **--withali** in previous versions of **cmalign.**

--mapstr Must be used in combination with **--mapali** <*f*>**.** Propogate structural information for any pseudoknots that exist in <*f*> to the output alignment. A similar option to this one was called **--withstr** in previous versions of **cmalign.**

--informat <*s*> Assert that the input <*seqfile*> is in format <*s*>*.* Do not run Babelfish format autodection. This increases the reliability of the program somewhat, because the Babelfish can make mistakes; particularly recommended for unattended, high-throughput runs of Infernal. Acceptable formats are: FASTA, GENBANK, and DDBJ. <*s*> is case-insensitive.

--outformat <*s*> Specify the output alignment format as <*s*>*.* Acceptable formats are: Pfam, AFA, A2M, Clustal, and Phylip. AFA is aligned fasta. Only Pfam and Stockholm alignment formats will include consensus structure annotation and posterior probability annotation of aligned residues.

--dnaout Output the alignments as DNA sequence alignments, instead of RNA ones.

--noprob Do not annotate the output alignment with posterior probabilities.

--matchonly Only include match columns in the output alignment, do not include any insertions relative to the consensus model. This option may be useful when creating very large alignments that require a lot of memory and disk space, most of which is necessary only to deal with insert columns that are gaps in most sequences.

--ileaved Output the alignment in interleaved Stockholm format of a fixed width that may be more convenient for examination. This was the default output alignment format of previous versions of **cmalign.** Note that **cmalign** requires more memory when this option is used. For this reason, **--ileaved** will only work for alignments of up to 100,000 sequences or a total of 100,000,000 aligned nucleotides.

--regress <*s*> Save an additional copy of the output alignment with no author information to file <*s*>.

--verbose Output additional information in the tabular scores output (output to stdout if **-o** is used, or to <*f*> if **--sfile** <*f*> is used). These are mainly useful for testing and debugging.

--cpu <*n*> Specify that <*n*> parallel CPU workers be used. If <*n*> is set as "0", then the program will be run in serial mode, without using threads. You can also control this number by setting an environment variable, *INFERNAL_NCPU.* This option will only be available if the machine on which Infernal was built is capable of using POSIX threading (see the Installation section of the user guide for more information).

--mpi Run as an MPI parallel program. This option will only be available if Infernal has been configured and built with the "--enable-mpi" flag (see the Installation section of the user guide for more information).

`cmbuild` - construct covariance model(s) from structurally annotated

Synopsis

cmbuild *[options]* <*cmfile_out*> <*msafile*>

Description

For each multiple sequence alignment in <*msafile*> build a covariance model and save it to a new file <*cmfile_out*>.

The alignment file must be in Stockholm or SELEX format, and must contain consensus secondary structure annotation. **cmbuild** uses the consensus structure to determine the architecture of the CM.

<*msafile*> may be '-' (dash), which means reading this input from *stdin* rather than a file. To use '-', you must also specify the alignment file format with **--informat** <*s*>**,** as in **--informat stockholm** (because of a current limitation in our implementation, MSA file formats cannot be autodetected in a nonrewindable input stream.)

<*cmfile_out*> may not be '-' *(stdout),* because sending the CM file to *stdout* would conflict with the other text output of the program.

In addition to writing CM(s) to <*cmfile_out*>*,* **cmbuild** also outputs a single line for each model created to stdout. Each line has the following fields: "aln": the index of the alignment used to build the CM; "idx": the index of the CM in the <*cmfile_out*>*;* "name": the name of the CM; "nseq": the number of sequences in the alignment used to build the CM; "eff_nseq": the effective number of sequences used to build the model; "alen": the length of the alignment used to build the CM; "clen": the number of columns from the alignment defined as consensus (match) columns; "bps": the number of basepairs in the CM; "bifs": the number of bifurcations in the CM; "rel entropy: CM": the total relative entropy of the model divided by the number of consensus columns; "rel entropy: HMM": the total relative entropy of the model ignoring secondary structure divided by the number of consensus columns. "description": description of the model/alignment.

Options

-h Help; print a brief reminder of command line usage and available options.

-n <*s*> Name the new CM <*s*>*.* The default is to use the name of the alignment (if one is present in the <*msafile*>*),* or, failing that, the name of the <*msafile*>*.* If <*msafile*> contains more than one alignment, *-n* doesn't work, and every alignment must have a name annotated in the <*msafile*> (as in Stockholm #=GF ID annotation).

-F Allow <*cmfile_out*> to be overwritten. Without this option, if <*cmfile_out*> already exists, **cmbuild** exits with an error.

-o <*f*> Direct the summary output to file <*f*>*,* rather than to *stdout.*

-O <*f*> After each model is constructed, resave annotated source alignments to a file <*f*> in Stockholm format. Sequences are annoted with what relative sequence weights were assigned. The alignments are also annotated with a reference annotation line indicating which columns were assigned as consensus. If the source alignment had reference annotation ("#=GC RF") it will be replaced with the consensus residue of the model for consensus columns and '.' for insert columns, unless the **--hand** option was used for specifying consensus positions, in which case it will be unchanged. **--devhelp** Print help, as with **-h,** but also include expert options that are not displayed with **-h.** These expert options are not expected to be relevant for the vast majority of users and so are not described in the manual page. The only resources for understanding what they actually do are the brief one-line descriptions output when **--devhelp** is enabled, and the source code.

Options Controlling Model Construction

These options control how consensus columns are defined in an alignment.

--fast Define consensus columns automatically as those that have a fraction >= **symfrac** of residues as opposed to gaps. (See below for the **--symfrac** option.) This is the default.

--hand Use reference coordinate annotation (#=GC RF line, in Stockholm) to determine which columns are consensus, and which are inserts. Any non-gap character indicates a consensus column. (For example, mark consensus columns with ”x”, and insert columns with ”.”.) This option was called **--rf** in previous versions of Infernal (0.1 through 1.0.2).

--symfrac <*x*> Define the residue fraction threshold necessary to define a consensus column when not using **--hand.** The default is 0.5. The symbol fraction in each column is calculated after taking relative sequence weighting into account. Setting this to 0.0 means that every alignment column will be assigned as consensus, which may be useful in some cases. Setting it to 1.0 means that only columns that include 0 gaps will be assigned as consensus. This option replaces the **--gapthresh** <*y*> option from previous versions of Infernal (0.1 through 1.0.2), with <*x*> equal to (1.0 - <*y*>*).* For example to reproduce behavior for a command of **cmbuild --gapthresh ” 0.8”** in a previous version, use **cmbuild --symfrac ” 0.2”** with this version.

--noss Ignore the secondary structure annotation, if any, in <*msafile*> and build a CM with zero basepairs. This model will be similar to a profile HMM and the **cmsearch** and **cmscan** programs will use HMM algorithms which are faster than CM ones for this model. Additionally, a zero basepair model need not be calibrated with **cmcalibrate** prior to running **cmsearch** with it. The **--noss** option must be used if there is no secondary structure annotation in **<msafile>.**

--rsearch <*f*> Parameterize emission scores a la RSEARCH, using the RIBOSUM matrix in file <*f*>*.* With **--rsearch** enabled, all alignments in <*msafile*> must contain exactly one sequence or the **--call** option must also be enabled. All positions in each sequence will be considered consensus ”columns”. Actually, the emission scores for these models will not be identical to RIBOSUM scores due of differences in the modelling strategy between Infernal and RSEARCH, but they will be as similar as possible. RIBOSUM matrix files are included with Infernal in the ”matrices/” subdirectory of the top-level ”infernal-xxx” directory. RIBOSUM matrices are substitution score matrices trained specifically for structural RNAs with separate single stranded residue and base pair substitution scores. For more information see the RSEARCH publication (Klein and Eddy, BMC Bioinformatics 4:44, 2003).

Other Model Construction Options

--null <*f*> Read a null model from <*f*>*.* The null model defines the probability of each RNA nucleotide in background sequence, the default is to use 0.25 for each nucleotide. The format of null files is specified in the user guide.

--prior <*f*> Read a Dirichlet prior from <*f*>*,* replacing the default mixture Dirichlet. The format of prior files is specified in the user guide.

Use **--devhelp** to see additional, otherwise undocumented, model construction options.

Options Controlling Relative Weights

cmbuild uses an ad hoc sequence weighting algorithm to downweight closely related sequences and upweight distantly related ones. This has the effect of making models less biased by uneven phylogenetic representation. For example, two identical sequences would typically each receive half the weight that one sequence would. These options control which algorithm gets used.

--wpb Use the Henikoff position-based sequence weighting scheme [Henikoff and Henikoff, J. Mol. Biol. 243:574, 1994]. This is the default.

--wgsc Use the Gerstein/Sonnhammer/Chothia weighting algorithm [Gerstein et al, J. Mol. Biol. 235:1067, 1994].

--wnone Turn sequence weighting off; e.g. explicitly set all sequence weights to 1.0.

--wgiven Use sequence weights as given in annotation in the input alignment file. If no weights were given, assume they are all 1.0. The default is to determine new sequence weights by the Gerstein/Sonnhammer/Chothia algorithm, ignoring any annotated weights.

--wblosum Use the BLOSUM filtering algorithm to weight the sequences, instead of the default GSC weighting. Cluster the sequences at a given percentage identity (see **--wid);** assign each cluster a total weight of 1.0, distributed equally amongst the members of that cluster.

--wid <*x*> Controls the behavior of the *--wblosum* weighting option by setting the percent identity for clustering the alignment to <*x*>*.*

Options Controlling Effective Sequence Number

After relative weights are determined, they are normalized to sum to a total effective sequence number, *eff_nseq.* This number may be the actual number of sequences in the alignment, but it is almost always smaller than that. The default entropy weighting method *(--eent)* reduces the effective sequence number to reduce the information content (relative entropy, or average expected score on true homologs) per consensus position. The target relative entropy is controlled by a two-parameter function, where the two parameters are settable with *--ere* and *--esigma.*

--eent Use the entropy weighting strategy to determine the effective sequence number that gives a target mean match state relative entropy. This option is the default, and can be turned off with **--enone.** The default target mean match state relative entropy is 0.59 bits for models with at least 1 basepair and 0.38 bits for models with zero basepairs, but changed with **--ere.** The default of 0.59 or 0.38 bits is automatically changed if the total relative entropy of the model (summed match state relative entropy) is less than a cutoff, which is is 6.0 bits by default, but can be changed with the expert, undocumented **--eX** option. If you really want to play with that option, consult the source code.

--enone Turn off the entropy weighting strategy. The effective sequence number is just the number of sequences in the alignment.

--ere <*x*> Set the target mean match state relative entropy as <*x*>*.* By default the target relative entropy per match position is 0.59 bits for models with at least 1 basepair and 0.38 for models with zero basepairs.

--eminseq <*x*> Define the minimum allowed effective sequence number as <*x*>*.*

--ehmmre <*x*> Set the target HMM mean match state relative entropy as <*x*>*.* Entropy for basepairing match states is calculated using marginalized basepair emission probabilities.

--eset <*x*> Set the effective sequence number for entropy weighting as <*x*>*.*

Options Controlling Filter P7 Hmm Construction

For each CM that **cmbuild** constructs, an accompanying filter p7 HMM is built from the input alignment as well. These options control filter HMM construction:

--p7ere <*x*> Set the target mean match state relative entropy for the filter p7 HMM as <*x*>*.* By default the target relative entropy per match position is 0.38 bits.

--p7ml Use a maximum likelihood p7 HMM built from the CM as the filter HMM. This HMM will be as similar as possible to the CM (while necessarily ignorant of secondary structure).

Use **--devhelp** to see additional, otherwise undocumented, filter HMM construction options.

Options Controlling Filter P7 Hmm Calibration

After building each filter HMM, **cmbuild** determines appropriate E-value parameters to use during filtering in **cmsearch** and **cmscan** by sampling a set of sequences and searching them with each HMM filter configuration and algorithm. **--EmN** *<n>* Set the number of sampled sequences for local MSV filter HMM calibration to *<n>.* 200 by default. **--EvN** *<n>* Set the number of sampled sequences for local Viterbi filter HMM calibration to *<n>.* 200 by default. **--ElfN** *<n>* Set the number of sampled sequences for local Forward filter HMM calibration to *<n>.* 200 by default. **--EgfN** *<n>* Set the number of sampled sequences for glocal Forward filter HMM calibration to *<n>.* 200 by default.

Use **--devhelp** to see additional, otherwise undocumented, filter HMM calibration options.

Options for Refining the Input Alignment

--refine *<f>* Attempt to refine the alignment before building the CM using expectation-maximization (EM). A CM is first built from the initial alignment as usual. Then, the sequences in the alignment are realigned optimally (with the HMM banded CYK algorithm, optimal means optimal given the bands) to the CM, and a new CM is built from the resulting alignment. The sequences are then realigned to the new CM, and a new CM is built from that alignment. This is continued until convergence, specifically when the alignments for two successive iterations are not significantly different (the summed bit scores of all the sequences in the alignment changes less than 1% between two successive iterations). The final alignment (the alignment used to build the CM that gets written to *<cmfile_out>)* is written to *<f>.*

-l With **--refine,** turn on the local alignment algorithm, which allows the alignment to span two or more subsequences if necessary (e.g. if the structures of the query model and target sequence are only partially shared), allowing certain large insertions and deletions in the structure to be penalized differently than normal indels. The default is to globally align the query model to the target sequences.

--gibbs Modifies the behavior of **--refine** so Gibbs sampling is used instead of EM. The difference is that during the alignment stage the alignment is not necessarily optimal, instead an alignment (parsetree) for each sequences is sampled from the posterior distribution of alignments as determined by the Inside algorithm. Due to this sampling step **--gibbs** is non-deterministic, so different runs with the same alignment may yield different results. This is not true when **--refine** is used without the **--gibbs** option, in which case the final alignment and CM will always be the same. When **--gibbs** is enabled, the **--seed** " <**n**>" option can be used to seed the random number generator predictably, making the results reproducible. The goal of the **--gibbs** option is to help expert RNA alignment curators refine structural alignments by allowing them to observe alternative high scoring alignments.

--seed *<n>* Seed the random number generator with *<n>,* an integer >= 0. This option can only be used in combination with **--gibbs.** If *<n>* is nonzero, stochastic sampling of alignments will be reproducible; the same command will give the same results. If *<n>* is 0, the random number generator is seeded arbitrarily, and stochastic samplings may vary from run to run of the same command. The default seed is 0.

--cyk With **--refine,** align with the CYK algorithm. By default the optimal accuracy algorithm is used. There is more information on this in the **cmalign** manual page.

--notrunc With **--refine,** turn off the the truncated alignment algorithm. There is more information on this in the **cmalign** manual page.

Use **--devhelp** to see additional, otherwise undocumented, alignment refinement options as well as other output file options and options for building multiple models for a single alignment.

`cmcalibrate` - fit exponential tails for covariance model E-value determination

Synopsis

cmcalibrate *[options] cmfile*

Description

cmcalibrate determines exponential tail parameters for E-value determination by generating random sequences, searching them with the CM and collecting the scores of the resulting hits. A histogram of the bit scores of the hits is fit to an exponential tail, and the parameters of the fitted tail are saved to the CM file. The exponential tail parameters are then used to estimate the statistical significance of hits found in **cmsearch** and **cmscan.**

A CM file must be calibrated with **cmcalibrate** before it can be used in **cmsearch** or **cmscan,** with a single exception: it is not necessary to calibrate CM files that include only models with zero basepairs before running **cmsearch.**

cmcalibrate is very slow. It takes a couple of hours to calibrate a single average sized CM on a single CPU. **cmcalibrate** will run in parallel on all available cores if Infernal was built on a system that supports POSIX threading (see the Installation section of the user guide for more information). Using <**n**> cores will result in roughly <**n**> -fold acceleration versus a single CPU. MPI (Message Passing Interface) can be also be used for parallelization with the **--mpi** option if Infernal was built with MPI enabled, but using more than 161 processors is not recommended because increasing past 161 won't accelerate the calibration. See the Installation seciton of the user guide for more information.

The **--forecast** option can be used to estimate how long the program will take to run for a given *cmfile* on the current machine. To predict the running time on <*n*> processors with MPI, additionally use the **--nforecast** <*n*> option.

The random sequences searched in **cmcalibrate** are generated by an HMM that was trained on real genomic sequences with various GC contents. The goal is to have the GC distributions in the random sequences be similar to those in actual genomic sequences.

Four rounds of searches and subsequent exponential tail fits are performed, one each for the four different CM algorithms that can be used in **cmsearch** and **cmscan:** glocal CYK, glocal Inside, local CYK and local Inside.

The E-values parameters determined by **cmcalibrate** are only used by the **cmsearch** and **cmscan** programs. If you are not going to use these programs then do not waste time calibrating your models.

Options

-h Help; print a brief reminder of command line usage and available options.

-L <*x*> Set the total length of random sequences to search to <*x*> megabases (Mb). By default, <*x*> is 1.6 Mb. Increasing <*x*> will make the exponential tail fits more precise and E-values more accurate, but will take longer (doubling <*x*> will roughly double the running time). Decreasing <*x*> is not recommended as it will make the fits less precise and the E-values less accurate.

Options for Predicting Required Time and Memory

--forecast Predict the running time of the calibration of *cmfile* (with provided options) on the current machine and exit. The calibration is not performed. The predictions should be considered rough estimates. If multithreading is enabled (see Installation section of user guide), the timing will take into account the number of available cores.

--nforecast <*n*> With **--forecast,** specify that <*n*> processors will be used for the calibration. This might be useful for predicting the running time of an MPI run with <*n*> processors.

--memreq Predict the amount of required memory for calibrating *cmfile* (with provided options) on the current machine and exit. The calibration is not performed.

Options Controlling Exponential Tail Fits

--gtailn *<x>* fit the exponential tail for glocal Inside and glocal CYK to the *<n>* highest scores in the histogram tail, where *<n>* is *<x>* times the number of Mb searched. The default value of *<x>* is 250. The value 250 was chosen because it works well empirically relative to other values.

--ltailn *<x>* fit the exponential tail for local Inside and local CYK to the *<n>* highest scores in the histogram tail, where *<n>* is *<x>* times the number of Mb searched. The default value of *<x>* is 750. The value 750 was chosen because it works well empirically relative to other values.

--tailp *<x>* Ignore the **--gtailn** and **--ltailn** prefixed options and fit the *<x>* fraction tail of the histogram to an exponential tail, for all search modes.

Optional Output Files

--hfile *<f>* Save the histograms fit to file *<f>*. The format of this file is two space delimited columns per line. The first column is the x-axis values of bit scores of each bin. The second column is the y-axis values of number of hits per bin. Each series is delimited by a line with a single character "&". The file will contain one series for each of the four exponential tail fits in the following order: glocal CYK, glocal Inside, local CYK, and local Inside.

--sfile *<f>* Save survival plot information to file *<f>.* The format of this file is two space delimited columns per line. The first column is the x-axis values of bit scores of each bin. The second column is the y-axis values of fraction of hits that meet or exceed the score for each bin. Each series is delimited by a line with a single character "&". The file will contain three series of data for each of the four CM search modes in the following order: glocal CYK, glocal Inside, local CYK, and local Inside. The first series is the empirical survival plot from the histogram of hits to the random sequence. The second series is the exponential tail fit to the empirical distribution. The third series is the exponential tail fit if lambda were fixed and set as the natural log of 2 (0.691314718).

--qqfile *<f>* Save quantile-quantile plot information to file *<f>.* The format of this file is two space delimited columns per line. The first column is the x-axis values, and the second column is the y-axis values. The distance of the points from the identity line (y=x) is a measure of how good the exponential tail fit is, the closer the points are to the identity line, the better the fit is. Each series is delimited by a line with a single character "&". The file will contain one series of empirical data for each of the four exponential tail fits in the following order: glocal CYK, glocal Inside, local CYK and local Inside.

--ffile *<f>* Save space delimited statistics of different exponential tail fits to file *<f>.* The file will contain the lambda and mu values for exponential tails fit to histogram tails of different sizes. The fields in the file are labelled informatively.

--xfile *<f>* Save a list of the scores in each fit histogram tail to file *<f>.* Each line of this file will have a different score indicating one hit existed in the tail with that score. Each series is delimited by a line with a single character "&". The file will contain one series for each of the four exponential tail fits in the following order: glocal CYK, glocal Inside, local CYK, and local Inside.

Other Options

--seed *<n>* Seed the random number generator with *<n>,* an integer >= 0. If *<n>* is nonzero, stochastic simulations will be reproducible; the same command will give the same results. If *<n>* is 0, the random number generator is seeded arbitrarily, and stochastic simulations will vary from run to run of the same command. The default seed is 181.

--beta *<x>* By default query-dependent banding (QDB) is used to accelerate the CM search algorithms with a beta tail loss probability of 1E-15. This beta value can be changed to *<x>* with **--beta** *<x>***.** The beta parameter is the amount of probability mass excluded during band

calculation, higher values of beta give greater speedups but sacrifice more accuracy than lower values. The default value used is 1E-15. (For more information on QDB see Nawrocki and Eddy, PLoS Computational Biology 3(3): e56.)

--nonbanded Turn off QDB during E-value calibration. This will slow down calibration.

--nonull3 Turn off the null3 post hoc additional null model. This is not recommended unless you plan on using the same option to **cmsearch** and/or **cmscan.**

--random Use the background null model of the CM to generate the random sequences, instead of the more realistic HMM. Unless the CM was built using the **--null** option to **cmbuild,** the background null model will be 25% each A, C, G and U.

--gc *<f>* Generate the random sequences using the nucleotide distribution from the sequence file *<f>.*

--cpu *<n>* Specify that *<n>* parallel CPU workers be used. If *<n>* is set as "0", then the program will be run in serial mode, without using threads. You can also control this number by setting an environment variable, *INFERNAL_NCPU.* This option will only be available if the machine on which Infernal was built is capable of using POSIX threading (see the Installation section of the user guide for more information).

--mpi Run as an MPI parallel program. This option will only be available if Infernal has been configured and built with the "--enable-mpi" flag (see the Installation section of the user guide for more information).

`cmconvert` - convert Infernal covariance model files

Synopsis

cmconvert *[options]* <*cmfile*>

Description

The **cmconvert** utility converts an input covariance model file to different Infernal formats.

By default, the input CM file can be any CM file created by Infernal version 1.0 or later; the output CM file is a current Infernal format. Files from versions older than version 1.0 cannot be converted.

<*cmfile*> may be '-' (dash), which means reading this input from *stdin* rather than a file.

Options

-h Help; print a brief reminder of command line usage and all available options.

-a Output profiles in ASCII text format. This is the default.

-b Output profiles in binary format.

-1 Output in legacy Infernal1 (Infernal v1.0 through v1.0.2) ASCII text format. Due to important changes between version v1.0.2 and v1.1, any E-value statistic parameters calculated by **cmcalibrate** in <**cmfile**> will not be written to the converted output file.

--mlhmm Do not output a CM file. Instead, output one maximum likelihood p7 HMM built from each CM in <**cmfile**> in HMMER3 ASCII text format. The HMM will have been constructed to be as similar as possible to the CM, without modeling secondary structure. This option could be useful for comparative studies of Infernal and HMMER3.

--fhmm Do not output a CM file. Instead, output the filter p7 HMM for each CM in <**cmfile**> in HMMER3 ASCII text format.

`cmemit` - sample sequences from a covariance model

Synopsis

cmemit *[options]* <*cmfile*>

Description

The **cmemit** program samples (emits) sequences from the covariance model(s) in <*cmfile*>, and writes them to output. Sampling sequences may be useful for a variety of purposes, including creating synthetic true positives for benchmarks or tests.

The default is to sample ten unaligned sequence from each CM. Alternatively, with the **-c** option, you can emit a single majority-rule consensus sequence; or with the **-a** option, you can emit an alignment.

The <*cmfile*> may contain a library of CMs, in which case each CM will be used in turn.

<*cmfile*> may be '-' (dash), which means reading this input from *stdin* rather than a file.

For models with zero basepairs, sequences are sampled from the profile HMM filter instead of the CM. However, since these models will be nearly identical (unless special options were used in **cmbuild** to prevent this), using the HMM instead of the CM will not change the output in a significant way, unless the **-l** option is used. With **-l,** the HMM will be configured for equiprobable model begin and end positions, while the CM will not. You can force **cmemit** to always sample from the CM with the **--nohmmonly** option.

Options

-h Help; print a brief reminder of command line usage and available options.

-o <*f*> Save the synthetic sequences to file <*f*> rather than writing them to stdout.

-N <*n*> Generate <*n*> sequences. The default value for <*n*> is 10.

-u Write the generated sequences in unaligned format (FASTA). This is the default behavior.

-a Write the generated sequences in an aligned format (STOCKHOLM) with consensus structure annotation rather than FASTA. Other output formats are possible with the **--outformat** option.

-c Predict a single majority-rule consensus sequence instead of sampling sequences from the CM's probability distribution. Highly conserved residues (base paired residues that score higher than 3.0 bits, or single stranded residues that score higher than 1.0 bits) are shown in upper case; others are shown in lower case.

-e <*n*> Embed the CM emitted sequences in a larger randomly generated sequence of length <*n*> generated from an HMM that was trained on real genomic sequences with various GC contents (the same HMM used by **cmcalibrate).** You can use the **--iid** option to generate 25% A, C, G, and U sequence instead. The CM emitted sequence will begin at a random position within the larger sequence and will be included in its entirety unless the **--u5p** or **--u3p** options are used. When **-e** is used in combination with **--u5p,** the CM emitted sequence will always begin at position 1 of the larger sequence and will be truncated 5'. When used in combination **--u3p** the CM emitted sequence will always end at position <*n*> of the larger sequence and will be truncated 3'.

-l Configure the CMs into local mode before emitting sequences. By default the model will be in global mode. In local mode, large insertions and deletions are more common than in global mode.

Options for Truncating Emitted Sequences

--u5p Truncate all emitted sequences at a randomly chosen start position <**n**>**,** by only outputting residues beginning at <**n**>**.** A different start point is randomly chosen for each sequence.

--u3p Truncate all emitted sequences at a randomly chosen end position <**n**>**,** by only outputting residues up to position <**n**>**.** A different end point is randomly chosen for each sequence.

--a5p <*n*> In combination with the **-a** option, truncate the emitted alignment at a randomly chosen start match position <*n*>*,* by only outputting alignment columns for positions after match state <*n*> - 1. <*n*> must be an integer between 0 and the consensus length of the model (which can be determined using the **cmstat** program. As a special case, using 0 as <*n*> will result in a randomly chosen start position.

--a3p <*n*> In combination with the **-a** option, truncate the emitted alignment at a randomly chosen end match position <*n*>*,* by only outputting alignment columns for positions before match state <*n*> + 1. <*n*> must be an integer between 1 and the consensus length of the model (which can be determined using the **cmstat** program). As a special case, using 0 as <*n*> will result in a randomly chosen end position.

Other Options

--seed <*n*> Seed the random number generator with <*n*>*,* an integer >= 0. If <*n*> is nonzero, stochastic sampling of sequences will be reproducible; the same command will give the same results. If <*n*> is 0, the random number generator is seeded arbitrarily, and stochastic samplings will vary from run to run of the same command. The default seed is 0.

--iid With **-e,** generate the larger sequences as 25% each A, C, G and U.

--rna Specify that the emitted sequences be output as RNA sequences. This is true by default.

--dna Specify that the emitted sequences be output as DNA sequences. By default, the output alphabet is RNA.

--idx <*n*> Specify that the emitted sequences be named starting with <*modelname*>*.*<*n*>*.* By default <*n*> is 1.

--outformat <*s*> With **-a,** specify the output alignment format as <*s*>*.* Acceptable formats are: Pfam, AFA, A2M, Clustal, and Phylip. AFA is aligned fasta. Only Pfam and Stockholm alignment formats will include consensus structure annotation.

--tfile <*f*> Dump tabular sequence parsetrees (tracebacks) for each emitted sequence to file <*f*>*.* Primarily useful for debugging.

--exp <*x*> Exponentiate the emission and transition probabilities of the CM by <*x*> and then renormalize those distributions before emitting sequences. This option changes the CM probability distribution of parsetrees relative to default. With <*x*> less than 1.0 the emitted sequences will tend to have lower bit scores upon alignment to the CM. With <x> greater than 1.0, the emitted sequences will tend to have higher bit scores upon alignment to the CM. This bit score difference will increase as <x> moves further away from 1.0 in either direction. If <x> equals 1.0, this option has no effect relative to default. This option is useful for generating sequences that are either more difficult (<*x*> < 1.0) or easier (<*x*> > 1.0) for the CM to distinguish as homologous from background, random sequence.

--hmmonly Emit from the filter profile HMM instead of the CM.

--nohmmonly Never emit from the filter profile HMM, always use the CM, even for models with zero basepairs.

`cmfetch` - retrieve covariance model(s) from a file

Synopsis

cmfetch *[options]* <*cmfile*> <*key*> (retrieves CM named <key>)

cmfetch -f *[options]* <*cmfile*> <*keyfile*> (retrieves all CMs listed in <keyfile>)

cmfetch --index *[options]* <*cmfile*> (indexes <cmfile> for fetching)

Description

Retrieves one or more CMs from an <*cmfile*> (a large Rfam database, for example).

To enable very fast retrieval, index the <*cmfile*> first, using **cmfetch --index.** The index is a binary file named <*cmfile*>*.ssi.*

The default mode is to retrieve a single CM by name or accession, called the <*key*>*.* For example:

% cmfetch Rfam.cm tRNA

% cmfetch Rfam.cm RF00005

With the *-f* option, a <*keyfile*> containing a list of one or more keys is read instead. The first whitespace-delimited field on each non-blank non-comment line of the <*keyfile*> is used as a <*key*>*,* and any remaining data on the line is ignored. This allows a variety of whitespace delimited datafiles to be used as <*keyfile*>*s.*

When using *-f* and a <*keyfile*>*,* if <**cmfile**> has been indexed, the keys are retrieved in the order they occur in the <*keyfile*>*,* but if <**cmfile**> isn't indexed, keys are retrieved in the order they occur in the <**cmfile**>**.** This is a side effect of an implementation that allows multiple keys to be retrieved even if the <**cmfile**> is a nonrewindable stream, like a standard input pipe.

In normal use (without *--index* or *-f* options), <*cmfile*> may be '-' (dash), which means reading input from *stdin* rather than a file. With the *--index* option, <*cmfile*> may not be '-'; it does not make sense to index a standard input stream. With the *-f* option, either <*cmfile*> or <*keyfile*> (but not both) may be '-'. It is often particularly useful to read <*keyfile*> from standard input, because this allows use to use arbitrary command line invocations to create a list of CM names or accessions, then fetch them all to a new file, just with one command.

By default, the CM is printed to standard output in Infernal-1.1 format.

Options

-h Help; print a brief reminder of command line usage and all available options.

-f The second commandline argument is a <*keyfile*> instead of a single <*key*>*.* The first field on each line of the <*keyfile*> is used as a retrieval <*key*> (a CM name or accession). Blank lines and comment lines (that start with a # character) are ignored.

-o <*f*> Output CM(s) to file <*f*> instead of to standard output.

-O Output CM(s) to individual file(s) named <*key*> instead of standard output. With the **-f** option, this can result in many files being created.

--index Instead of retrieving one or more profiles from <*cmfile*>*,* index the <*cmfile*> for future retrievals. This creates a <*cmfile*>*.ssi* binary index file.

`cmpress` - prepare a covariance model database for cmscan

Synopsis

cmpress *[options]* <*cmfile*>

Description

Starting from a CM database <*cmfile*> in standard Infernal-1.1 format, construct binary compressed datafiles for **cmscan.** The *cmpress* step is required for **cmscan** to work.

The <*cmfile*> must be have already been calibrated with **cmcalibrate** for **cmpress** to work.

Four files are created: <*cmfile*>*.i1m,* <*cmfile*>*.i1i,* <*cmfile*>*.i1f,* and <*cmfile*>*.i1p.* The <*cmfile*>*.i1m* file contains the covariance models, associated filter p7 profile HMMs and their annotation in a binary format. The <*cmfile*>*.i1i* file is an SSI index for the <*cmfile*>*.i1m* file. The <*cmfile*>*.i1f* file contains precomputed data structures for the fast heuristic filter (the SSV filter) for the filter p7 profile HMMs in <*cmfile*>*.* The <*cmfile*>*.i1p* file contains precomputed data structures for the rest of each profile filter p7 HMM.

<*cmfile*> may not be '-' (dash); running **cmpress** on a standard input stream rather than a file is not allowed.

Options

-h Help; print a brief reminder of command line usage and all available options.

-F Force; overwrites any previous cmpress'ed datafiles. The default is to bitch about any existing files and ask you to delete them first.

`cmscan` - search sequence(s) against a covariance model database

Synopsis

cmscan *[options]* <*cmdb*> <*seqfile*>

Description

cmscan is used to search sequences against collections of covariance models. For each sequence in <*seqfile*>*,* use that query sequence to search the target database of CMs in <*cmdb*>*,* and output ranked lists of the CMs with the most significant matches to the sequence.

The <*seqfile*> may contain more than one query sequence. It can be in FASTA format, or several other common sequence file formats (genbank, embl, and among others), or in alignment file formats (stockholm, aligned fasta, and others). See the *--qformat* option for a complete list.

The <*cmdb*> needs to be press'ed using **cmpress** before it can be searched with **cmscan.** This creates four binary files, suffixed .i1{fimp}. Additionally, <*cmdb*> must have been calibrated for E-values with **cmcalibrate** before being press'ed with **cmpress.**

The query <*seqfile*> may be '-' (a dash character), in which case the query sequences are read from a <stdin> pipe instead of from a file. The <*cmdb*> cannot be read from a <stdin> stream, because it needs to have those four auxiliary binary files generated by **cmpress.**

The output format is designed to be human-readable, but is often so voluminous that reading it is impractical, and parsing it is a pain. The **--tblout** option saves output in a simple tabular format that is concise and easier to parse. The **--fmt** *2* option modifies the format of the tabular output by adding several fields, including markup of overlapping hits, as described in section 6 of the Infernal user guide. The **-o** option allows redirecting the main output, including throwing it away in /dev/null.

cmscan reexamines the 5' and 3' termini of target sequences using specialized algorithms for detection of *truncated* hits, in which part of the 5' and/or 3' end of the actual full length homologous sequence is missing in the target sequence file. These types of hits will be most common in sequence files consisting of unassembled sequencing reads. By default, any 5' truncated hit is required to include the first residue of the target sequence it derives from in <*seqfile*>*,* and any 3' truncated hit is required to include the final residue of the target sequence it derives from. Any 5' and 3' truncated hit must include the first and final residue of the target sequence it derives from. The **--anytrunc** option will relax the requirements for hit inclusion of sequence endpoints, and truncated hits are allowed to start and stop at any positions of target sequences. Importantly though, with **--anytrunc,** hit E-values will be less accurate because model calibration does not consider the possibility of truncated hits, so use it with caution. The **--notrunc** option can be used to turn off truncated hit detection. **--notrunc** will reduce the running time of **cmscan,** most significantly for target <*seqfile*> files that include many short sequences. Truncated hit detection is automatically turned off when the **--max, --nohmm, --qdb,** or **--nonbanded** options are used because it relies on the use of an accelerated HMM banded alignment strategy that is turned off by any of those options.

Options

-h Help; print a brief reminder of command line usage and all available options.

-g Turn on the *glocal* alignment algorithm, global with respect to the query model and local with respect to the target database. By default, the local alignment algorithm is used which is local with respect to both the target sequence and the model. In local mode, the alignment to span two or more subsequences if necessary (e.g. if the structures of the query model and target sequence are only partially shared), allowing certain large insertions and deletions in the structure to be penalized differently than normal indels. Local mode performs better on empirical benchmarks and is significantly more sensitive for remote homology detection. Empirically, glocal searches return many fewer hits than local searches, so glocal may be desired for some applications.

-Z *<x>* Calculate E-values as if the search space size was *<x>* megabases (Mb). Without the use of this option, the search space size changes for each query sequence, it is defined as the length of the current query sequence times 2 (because both strands of the sequence will be searched) times the number of CMs in *<cmdb>.*

--devhelp Print help, as with **-h,** but also include expert options that are not displayed with **-h.** These expert options are not expected to be relevant for the vast majority of users and so are not described in the manual page. The only resources for understanding what they actually do are the brief one-line descriptions output when **--devhelp** is enabled, and the source code.

Options for Controlling Output

-o *<f>* Direct the main human-readable output to a file *<f>* instead of the default stdout.

--tblout *<f>* Save a simple tabular (space-delimited) file summarizing the hits found, with one data line per hit. The format of this file is described in section 6 of the Infernal user guide.

--fmt *<n>* specify the format of the tabular output file specified with **--tblout** *<f>* be in format *<n>.* Possible values for *<n>* are 1 or 2. By default *<n>* is 1 when **--tblout** is used without **--fmt.** With **--fmt** *2* nine additional fields are added to the tabular output file, most of which pertain to the annotation of overlapping hits. See section 6 the Infernal user guide for a description of both formats.

--acc Use accessions instead of names in the main output, where available for profiles and/or sequences.

--noali Omit the alignment section from the main output. This can greatly reduce the output volume.

--notextw Unlimit the length of each line in the main output. The default is a limit of 120 characters per line, which helps in displaying the output cleanly on terminals and in editors, but can truncate target profile description lines.

--textw *<n>* Set the main output's line length limit to *<n>* characters per line. The default is 120.

--verbose Include extra search pipeline statistics in the main output, including filter survival statistics for truncated hit detection and number of envelopes discarded due to matrix size overflows.

Options Controlling Reporting Thresholds

Reporting thresholds control which hits are reported in output files (the main output and *--tblout)* Hits are ranked by statistical significance (E-value). By default, all hits with an E-value <= 10 are reported. The following options allow you to change the default E-value reporting thresholds, or to use bit score thresholds instead.

-E *<x>* In the per-target output, report target sequences with an E-value of <= *<x>.* The default is 10.0, meaning that on average, about 10 false positives will be reported per query, so you can see the top of the noise and decide for yourself if it's really noise.

-T *<x>* Instead of thresholding per-CM output on E-value, report target sequences with a bit score of >= *<x>.*

Options for Inclusion Thresholds

Inclusion thresholds are stricter than reporting thresholds. Inclusion thresholds control which hits are considered to be reliable enough to be included in a possible subsequent search round, or marked as significant ("!") as opposed to questionable ("?") in hit output.

--incE *<x>* Use an E-value of <= *<x>* as the hit inclusion threshold. The default is 0.01, meaning that on average, about 1 false positive would be expected in every 100 searches with different query sequences.

--incT *<x>* Instead of using E-values for setting the inclusion threshold, instead use a bit score of >= *<x>* as the hit inclusion threshold. By default this option is unset.

Options for Model-specific Score Thresholding

Curated CM databases may define specific bit score thresholds for each CM, superseding any thresholding based on statistical significance alone.

To use these options, the profile must contain the appropriate (GA, TC, and/or NC) optional score threshold annotation; this is picked up by **cmbuild** from Stockholm format alignment files. Each thresholding option has a score of <*x*> bits, and acts as if **-T** <*x*> **--incT** <*x*> has been applied specifically using each model's curated thresholds.

--cut_ga Use the GA (gathering) bit scores in the model to set hit reporting and inclusion thresholds. GA thresholds are generally considered to be the reliable curated thresholds defining family membership; for example, in Rfam, these thresholds define what gets included in Rfam Full alignments based on searches with Rfam Seed models.

--cut_nc Use the NC (noise cutoff) bit score thresholds in the model to set hit reporting and inclusion thresholds. NC thresholds are generally considered to be the score of the highest-scoring known false positive.

--cut_tc Use the TC (trusted cutoff) bit score thresholds in the model to set hit reporting and inclusion thresholds. TC thresholds are generally considered to be the score of the lowest-scoring known true positive that is above all known false positives.

Options Controlling the Acceleration Pipeline

Infernal searches are accelerated in a six-stage filter pipeline. The first five stages use a profile HMM to define envelopes that are passed to the stage six CM CYK filter. Any envelopes that survive all filters are assigned final scores using the the CM Inside algorithm.

The profile HMM filter is built by the **cmbuild** program and is stored in <*cmfile*>*.*

Each successive filter is slower than the previous one, but better than it at disciminating between subsequences that may contain high-scoring CM hits and those that do not. The first three HMM filter stages are the same as those used in HMMER3. Stage 1 (F1) is the local HMM SSV filter modified for long sequences. Stage 2 (F2) is the local HMM Viterbi filter. Stage 3 (F3) is the local HMM Forward filter. Each of the first three stages uses the profile HMM in local mode, which allows a target subsequence to align to any region of the HMM. Stage 4 (F4) is a glocal HMM filter, which requires a target subsequence to align to the full-length profile HMM. Stage 5 (F5) is the glocal HMM envelope definition filter, which uses HMMER3's domain identification heursitics to define envelope boundaries. After each stage from 2 to 5 a bias filter step (F2b, F3b, F4b, and F5b) is used to remove sequences that appear to have passed the filter due to biased composition alone. Any envelopes that survive stages F1 through F5b are then passed with the local CM CYK filter. The CYK filter uses constraints (bands) derived from an HMM alignment of the envelope to reduce the number of required calculations and save time. Any envelopes that pass CYK are scored with the local CM Inside algorithm, again using HMM bands for acceleration.

The default filter thresholds that define the minimum score required for a subsequence to survive each stage are defined based on the size of the search space (Z), which is defined as the length of the current query sequence times 2 (because both strands will be searched) times the number of profiles in <*cmdb*>*.* However, if either the **-Z** <*x*> or **--FZ** <*x*> options are used then the search space will be considered to be <*x*> for purposes of defining the filter thresholds.

For larger databases, the filters are more strict leading to more acceleration but potentially a greater loss of sensitivity. The rationale is that for larger databases, hits must have higher scores to achieve statistical significance, so stricter filtering that removes lower scoring insignificant hits is acceptable.

The P-value thresholds for all possible search space sizes and all filter stages are listed next. (A P-value threshold of 0.01 means that roughly 1% of the highest scoring nonhomologous subsequence are expected to pass the filter.) Z is defined as the number of nucleotides in the complete target sequence file times 2 because both strands will be searched with each model.

If Z is less than 2 Mb: F1 is 0.35; F2 and F2b are off; F3, F3b, F4, F4b and F5 are 0.02; F6 is 0.0001.

If Z is between 2 Mb and 20 Mb: F1 is 0.35; F2 and F2b are off; F3, F3b, F4, F4b and F5 are 0.005; F6 is 0.0001.

If Z is between 20 Mb and 200 Mb: F1 is 0.35; F2 and F2b are 0.15; F3, F3b, F4, F4b and F5 are 0.003; F6 is 0.0001.

If Z is between 200 Mb and 2 Gb: F1 is 0.15; F2 and F2b are 0.15; F3, F3b, F4, F4b, F5, and F5b are 0.0008; and F6 is 0.0001.

If Z is between 2 Gb and 20 Gb: F1 is 0.15; F2 and F2b are 0.15; F3, F3b, F4, F4b, F5, and F5b are 0.0002; and F6 is 0.0001.

If Z is more than 20 Gb: F1 is 0.06; F2 and F2b are 0.02; F3, F3b, F4, F4b, F5, and F5b are 0.0002; and F6 is 0.0001.

These thresholds were chosen based on performance on an internal benchmark testing many different possible settings.

There are five options for controlling the general filtering level. These options are, in order from least strict (slowest but most sensitive) to most strict (fastest but least sensitive): **--max, --nohmm, --mid, --default,** (this is the default setting) **--rfam.** and **--hmmonly.** With **--default** the filter thresholds will be database-size dependent. See the explanation of each of these individual options below for more information.

Additionally, an expert user can precisely control each filter stage score threshold with the **--F1, --F1b, --F2, --F2b, --F3, --F3b, --F4, --F4b, --F5, --F5b,** and **--F6** options. As well as turn each stage on or off with the **--noF1, --doF1b, --noF2, --noF2b, --noF3, --noF3b, --noF4, --noF4b, --noF5,** and **--noF6.** options. These options are only displayed if the **--devhelp** option is used to keep the number of displayed options with **-h** reasonable, and because they are only expected to be useful to a small minority of users.

As a special case, for any models in <*cmfile*> which have zero basepairs, profile HMM searches are run instead of CM searches. HMM algorithms are more efficient than CM algorithms, and the benefit of CM algorithms is lost for models with no secondary structure (zero basepairs). These profile HMM searches will run significantly faster than the CM searches. You can force HMM-only searches with the **--hmmonly** option. For more information on HMM-only searches see the user guide.

--max Turn off all filters, and run non-banded Inside on every full-length target sequence. This increases sensitivity somewhat, at an extremely large cost in speed.

--nohmm Turn off all HMM filter stages (F1 through F5b). The CYK filter, using QDBs, will be run on every full-length target sequence and will enforce a P-value threshold of 0.0001. Each subsequence that survives CYK will be passed to Inside, which will also use QDBs (but a looser set). This increases sensitivity somewhat, at a very large cost in speed.

--mid Turn off the HMM SSV and Viterbi filter stages (F1 through F2b). Set remaining HMM filter thresholds (F3 through F5b) to 0.02 by default, but changeable to <*x*> with **--Fmid** <*x*> sequence. This may increase sensitivity, at a significant cost in speed.

--default Use the default filtering strategy. This option is on by default. The filter thresholds are determined based on the database size.

--rfam Use a strict filtering strategy devised for large databases (more than 20 Gb). This will accelerate the search at a potential cost to sensitivity.

--hmmonly Only use the filter profile HMM for searches, do not use the CM. Only filter stages F1 through F3 will be executed, using strict P-value thresholds (0.02 for F1, 0.001 for F2 and 0.00001 for F3). Additionally a bias composition filter is used after the F1 stage (with P=0.02 survival threshold). Any hit that survives all stages and has an HMM E-value or bit score above the reporting threshold will be output. The user can change the HMM-only filter thresholds and options with **--hmmF1, --hmmF2, --hmmF3, --hmmnobias, --hmmnonull2,** and **--hmmmax.** By default, searches for any model with zero basepairs will be run in HMM-only mode. This can be turned off, forcing CM searches for these models with the **--nohmmonly** option.

--FZ <*x*> Set filter thresholds as the defaults used if the database were <**x**> megabases (Mb). If used with <**x**> greater than 20000 (20 Gb) this option has the same effect as **--rfam.**

--Fmid <*x*> With the **--mid** option set the HMM filter thresholds (F3 through F5b) to <*x*>. By default, <*x*> is 0.02.

Other Options

--notrunc Turn off truncated hit detection.

--anytrunc Allow truncated hits to begin and end at any position in a target sequence. By default, 5' truncated hits must include the first residue of their target sequence and 3' truncated hits must include the final residue of their target sequence. With this option you may observe fewer full length hits that extend to the beginning and end of the query CM.

--nonull3 Turn off the null3 CM score corrections for biased composition. This correction is not used during the HMM filter stages.

--mxsize <*x*> Set the maximum allowable CM DP matrix size to <*x*> megabytes. By default this size is 128 Mb. This should be large enough for the vast majority of searches, especially with smaller models. If **cmscan** encounters an envelope in the CYK or Inside stage that requires a larger matrix, the envelope will be discounted from consideration. This behavior is like an additional filter that prevents expensive (slow) CM DP calculations, but at a potential cost to sensitivity. Note that if **cmscan** is being run in <*n*> multiple threads on a multicore machine then each thread may have an allocated matrix of up to size <*x*> Mb at any given time.

--smxsize <*x*> Set the maximum allowable CM search DP matrix size to <*x*> megabytes. By default this size is 128 Mb. This option is only relevant if the CM will not use HMM banded matrices, i.e. if the **--max, --nohmm, --qdb, --fqdb, --nonbanded,** or **--fnonbanded** options are also used. Note that if **cmsearch** is being run in <*n*> multiple threads on a multicore machine then each thread may have an allocated matrix of up to size <*x*> Mb at any given time.

--cyk Use the CYK algorithm, not Inside, to determine the final score of all hits.

--acyk Use the CYK algorithm to align hits. By default, the Durbin/Holmes optimal accuracy algorithm is used, which finds the alignment that maximizes the expected accuracy of all aligned residues.

--wcx <*x*> For each CM, set the W parameter, the expected maximum length of a hit, to <*x*> times the consensus length of the model. By default, the W parameter is read from the CM file and was calculated based on the transition probabilities of the model by **cmbuild.** You can find out what the default W is for a model using **cmstat.** This option should be used with caution as it impacts the filtering pipeline at several different stages in nonobvious ways. It is only recommended for expert users searching for hits that are much longer than any of the homologs used to build the model in **cmbuild,** e.g. ones with large introns or other large insertions. It cannot be used in combination with the **--nohmm, --fqdb** or **--qdb** options because in those cases W is limited by query-dependent bands.

--toponly Only search the top (Watson) strand of target sequences in <*seqfile*>*.* By default, both strands are searched. This will halve the search space size (Z).

--bottomonly Only search the bottom (Crick) strand of target sequences in <*seqfile*>*.* By default, both strands are searched. This will halve the search space size (Z).

--qformat <*s*> Assert that the query sequence database file is in format <*s*>*.* Accepted formats include *fasta, embl, genbank, ddbj, stockholm, pfam, a2m, afa, clustal,* and *phylip* The default is to autodetect the format of the file.

--glist <*f*> Configure a subset of models from <*cmfile*> in glocal alignment mode, instead of local mode, namely the models listed in file <*f*>*.* Configure all other models (those not listed in <*f*>*)* in local mode. This option is incompatible with *-g.* File <*f*> must list valid names of models from <*cmfile*>*,* each separated by any whitespace character (e.g. a newline character).

--clanin <*f*> Read clan information on the models in <*cmfile*> from file <*f*>*.* Not all models in <*cmfile*> need to be a member of a clan. This option must be used in combination with **--fmt** *2* and **--tblout** because clan annotation is only output in format 2 of the tabular output file. See section 9 of the Infernal user guide for specifications on the format of the clan input file <*f*>*.*

--oclan Only mark overlaps between models in the same clan. This option must be used in combination with **--fmt** *2* , **--tblout** and **--clanin** because clan annotation is only output in format 2 of the tabular output file, and clan information can only be input using the **--clanin** option.

--oskip *<f>* Omit any hit h from the tabular output file that satisifies the following: another hit h2 overlaps with h and the E-value of h2 is lower than that of h. Hit h will not appear in the tabular output file, although it will still exist in the standard output. This option must be used in combination with **--fmt** *2* **--tblout** because overlap annotation is only output in format 2 of the tabular output file. When used in combination with **--oclan** only hits h that satisfy the following are omitted: another hit h2 overlaps with h, the E-value of h2 is lower than that of h, and both h and h2 are hits to models that are in the same clan.

--cpu *<n>* Set the number of parallel worker threads to *<n>.* By default, Infernal sets this to the number of CPU cores it detects in your machine - that is, it tries to maximize the use of your available processor cores. Setting *<n>* higher than the number of available cores is of little if any value, but you may want to set it to something less. You can also control this number by setting an environment variable, *INFERNAL_NCPU.* This option is only available if Infernal was compiled with POSIX threads support. This is the default, but it may have been turned off at compile-time for your site or machine for some reason.

--stall For debugging the MPI master/worker version: pause after start, to enable the developer to attach debuggers to the running master and worker(s) processes. Send SIGCONT signal to release the pause. (Under gdb: *(gdb)* signal SIGCONT) (Only available if optional MPI support was enabled at compile-time.)

--mpi Run in MPI master/worker mode, using *mpirun.* (Only available if optional MPI support was enabled at compile-time.)

`cmsearch` - search covariance model(s) against a sequence database

Synopsis

cmsearch *[options]* <*cmfile*> <*seqdb*>

Description

cmsearch is used to search one or more covariance models (CMs) against a sequence database. For each CM in <*cmfile*>*,* use that query CM to search the target database of sequences in <*seqdb*>*,* and output ranked lists of the sequences with the most significant matches to the CM. To build CMs from multiple alignments, see **cmbuild.**

The query <*cmfile*> must have been calibrated for E-values with **cmcalibrate.** As a special exception, any models in <*cmfile*> that have zero basepairs need not be calibrated. For these models, profile HMM search algorithms will be used instead of CM ones, as discussed further below.

The query <*cmfile*> may be '-' (a dash character), in which case the query CM input will be read from a <stdin> pipe instead of from a file. The <*seqdb*> may not be '-' because the current implementation needs to be able to rewind the database, which is not possible with *stdin* input.

The output format is designed to be human-readable, but is often so voluminous that reading it is impractical, and parsing it is a pain. The **--tblout** option saves output in a simple tabular format that is concise and easier to parse. The **-o** option allows redirecting the main output, including throwing it away in /dev/null.

cmsearch reexamines the 5' and 3' termini of target sequences using specialized algorithms for detection of *truncated* hits, in which part of the 5' and/or 3' end of the actual full length homologous sequence is missing in the target sequence file. These types of hits will be most common in sequence files consisting of unassembled sequencing reads. By default, any 5' truncated hit is required to include the first residue of the target sequence it derives from in <*seqdb*>*,* and any 3' truncated hit is required to include the final residue of the target sequence it derives from. Any 5' and 3' truncated hit must include the first and final residue of the target sequence it derives from. The **--anytrunc** option will relax the requirements for hit inclusion of sequence endpoints, and truncated hits are allowed to start and stop at any positions of target sequences. Importantly though, with **--anytrunc,** hit E-values will be less accurate because model calibration does not consider the possibility of truncated hits, so use it with caution. The **--notrunc** option can be used to turn off truncated hit detection. **--notrunc** will reduce the running time of **cmsearch,** most significantly for target <*seqdb*> files that include many short sequences.

Truncated hit detection is automatically turned off when the **--max, --nohmm, --qdb,** or **--nonbanded** options are used because it relies on the use of an accelerated HMM banded alignment strategy that is turned off by any of those options.

Options

-h Help; print a brief reminder of command line usage and all available options.

-g Turn on the *glocal* alignment algorithm, global with respect to the query model and local with respect to the target database. By default, the local alignment algorithm is used which is local with respect to both the target sequence and the model. In local mode, the alignment to span two or more subsequences if necessary (e.g. if the structures of the query model and target sequence are only partially shared), allowing certain large insertions and deletions in the structure to be penalized differently than normal indels. Local mode performs better on empirical benchmarks and is significantly more sensitive for remote homology detection. Empirically, glocal searches return many fewer hits than local searches, so glocal may be desired for some applications. With **-g,** all models must be calibrated, even those with zero basepairs.

-Z <*x*> Calculate E-values as if the search space size was <*x*> megabases (Mb). Without the use of this option, the search space size is defined as the total number of nucleotides in <*seqdb*> times 2, because both strands of each target sequence will be searched.

--devhelp Print help, as with **-h,** but also include expert options that are not displayed with **-h.** These expert options are not expected to be relevant for the vast majority of users and so are not described in the manual page. The only resources for understanding what they actually do are the brief one-line descriptions output when **--devhelp** is enabled, and the source code.

Options for Controlling Output

-o <*f*> Direct the main human-readable output to a file <*f*> instead of the default stdout.

-A <*f*> Save a multiple alignment of all significant hits (those satisfying *inclusion* thresholds) to the file <*f*>.

--tblout <*f*> Save a simple tabular (space-delimited) file summarizing the hits found, with one data line per hit. The format of this file is described in section 6 of the Infernal user guide.

--acc Use accessions instead of names in the main output, where available for profiles and/or sequences.

--noali Omit the alignment section from the main output. This can greatly reduce the output volume.

--notextw Unlimit the length of each line in the main output. The default is a limit of 120 characters per line, which helps in displaying the output cleanly on terminals and in editors, but can truncate target profile description lines.

--textw <*n*> Set the main output's line length limit to <*n*> characters per line. The default is 120.

--verbose Include extra search pipeline statistics in the main output, including filter survival statistics for truncated hit detection and number of envelopes discarded due to matrix size overflows.

Options Controlling Reporting Thresholds

Reporting thresholds control which hits are reported in output files (the main output and *--tblout)* Hits are ranked by statistical significance (E-value). By default, all hits with an E-value <= 10 are reported. The following options allow you to change the default E-value reporting thresholds, or to use bit score thresholds instead.

-E <*x*> In the per-target output, report target sequences with an E-value of <= <*x*>. The default is 10.0, meaning that on average, about 10 false positives will be reported per query, so you can see the top of the noise and decide for yourself if it's really noise.

-T <*x*> Instead of thresholding per-CM output on E-value, report target sequences with a bit score of >= <*x*>.

Options for Inclusion Thresholds

Inclusion thresholds are stricter than reporting thresholds. Inclusion thresholds control which hits are considered to be reliable enough to be included in an output alignment or in a possible subsequent search round, or marked as significant ("!") as opposed to questionable ("?") in hit output.

--incE <*x*> Use an E-value of <= <*x*> as the hit inclusion threshold. The default is 0.01, meaning that on average, about 1 false positive would be expected in every 100 searches with different query sequences.

--incT <*x*> Instead of using E-values for setting the inclusion threshold, instead use a bit score of >= <*x*> as the hit inclusion threshold. By default this option is unset.

Options for Model-specific Score Thresholding

Curated CM databases may define specific bit score thresholds for each CM, superseding any thresholding based on statistical significance alone.

To use these options, the profile must contain the appropriate (GA, TC, and/or NC) optional score threshold annotation; this is picked up by **cmbuild** from Stockholm format alignment files. Each thresholding option has a score of <*x*> bits, and acts as if **-T** <*x*> **--incT** <*x*> has been applied specifically using each model's curated thresholds.

--cut_ga Use the GA (gathering) bit scores in the model to set hit reporting and inclusion thresholds. GA thresholds are generally considered to be the reliable curated thresholds defining family membership; for example, in Rfam, these thresholds define what gets included in Rfam Full alignments based on searches with Rfam Seed models.

--cut_nc Use the NC (noise cutoff) bit score thresholds in the model to set hit reporting and inclusion thresholds. NC thresholds are generally considered to be the score of the highest-scoring known false positive.

--cut_tc Use the TC (trusted cutoff) bit score thresholds in the model to set hit reporting and inclusion thresholds. TC thresholds are generally considered to be the score of the lowest-scoring known true positive that is above all known false positives.

Options Controlling the Acceleration Pipeline

Infernal 1.1 searches are accelerated in a six-stage filter pipeline. The first five stages use a profile HMM to define envelopes that are passed to the stage six CM CYK filter. Any envelopes that survive all filters are assigned final scores using the the CM Inside algorithm. (See the user guide for more information.)

The profile HMM filter is built by the **cmbuild** program and is stored in <*cmfile*>.

Each successive filter is slower than the previous one, but better than it at disciminating between subsequences that may contain high-scoring CM hits and those that do not. The first three HMM filter stages are the same as those used in HMMER3. Stage 1 (F1) is the local HMM SSV filter modified for long sequences. Stage 2 (F2) is the local HMM Viterbi filter. Stage 3 (F3) is the local HMM Forward filter. Each of the first three stages uses the profile HMM in local mode, which allows a target subsequence to align to any region of the HMM. Stage 4 (F4) is a glocal HMM filter, which requires a target subsequence to align to the full-length profile HMM. Stage 5 (F5) is the glocal HMM envelope definition filter, which uses HMMER3's domain identification heursitics to define envelope boundaries. After each stage from 2 to 5 a bias filter step (F2b, F3b, F4b, and F5b) is used to remove sequences that appear to have passed the filter due to biased composition alone. Any envelopes that survive stages F1 through F5b are then passed with the local CM CYK filter. The CYK filter uses constraints (bands) derived from an HMM alignment of the envelope to reduce the number of required calculations and save time. Any envelopes that pass CYK are scored with the local CM Inside algorithm, again using HMM bands for acceleration.

The default filter thresholds that define the minimum score required for a subsequence to survive each stage are defined based on the size of the database in <*seqdb*> (or the size <*x*> in megabases (Mb) specified by the **-Z** <*x*> or **--FZ** <*x*> options). For larger databases, the filters are more strict leading to more acceleration but potentially a greater loss of sensitivity. The rationale is that for larger databases, hits must have higher scores to achieve statistical significance, so stricter filtering that removes lower scoring insignificant hits is acceptable.

The P-value thresholds for all possible search space sizes and all filter stages are listed next. (A P-value threshold of 0.01 means that roughly 1% of the highest scoring nonhomologous subsequence are expected to pass the filter.) Z is defined as the number of nucleotides in the complete target sequence file times 2 because both strands will be searched with each model.

If Z is less than 2 Mb: F1 is 0.35; F2 and F2b are off; F3, F3b, F4, F4b and F5 are 0.02; F6 is 0.0001.

If Z is between 2 Mb and 20 Mb: F1 is 0.35; F2 and F2b are off; F3, F3b, F4, F4b and F5 are 0.005; F6 is 0.0001.

If Z is between 20 Mb and 200 Mb: F1 is 0.35; F2 and F2b are 0.15; F3, F3b, F4, F4b and F5 are 0.003; F6 is 0.0001.

If Z is between 200 Mb and 2 Gb: F1 is 0.15; F2 and F2b are 0.15; F3, F3b, F4, F4b, F5, and F5b are 0.0008; and F6 is 0.0001.

If Z is between 2 Gb and 20 Gb: F1 is 0.15; F2 and F2b are 0.15; F3, F3b, F4, F4b, F5, and F5b are 0.0002; and F6 is 0.0001.

If Z is more than 20 Gb: F1 is 0.06; F2 and F2b are 0.02; F3, F3b, F4, F4b, F5, and F5b are 0.0002; and F6 is 0.0001.

These thresholds were chosen based on performance on an internal benchmark testing many different possible settings.

There are five options for controlling the general filtering level. These options are, in order from least strict (slowest but most sensitive) to most strict (fastest but least sensitive): **--max, --nohmm, --mid, --default,** (this is the default setting), **--rfam.** and **--hmmonly.** With **--default** the filter thresholds will be database-size dependent. See the explanation of each of these individual options below for more information.

Additionally, an expert user can precisely control each filter stage score threshold with the **--F1, --F1b, --F2, --F2b, --F3, --F3b, --F4, --F4b, --F5, --F5b,** and **--F6** options. As well as turn each stage on or off with the **--noF1, --doF1b, --noF2, --noF2b, --noF3, --noF3b, --noF4, --noF4b, --noF5,** and **--noF6.** options. These options are only displayed if the **--devhelp** option is used to keep the number of displayed options with **-h** reasonable, and because they are only expected to be useful to a small minority of users.

As a special case, for any models in <*cmfile*> which have zero basepairs, profile HMM searches are run instead of CM searches. HMM algorithms are more efficient than CM algorithms, and the benefit of CM algorithms is lost for models with no secondary structure (zero basepairs). These profile HMM searches will run significantly faster than the CM searches. You can force HMM-only searches with the **--hmmonly** option. For more information on HMM-only searches see the description of the **--hmmonly** option below, and the user guide.

--max Turn off all filters, and run non-banded Inside on every full-length target sequence. This increases sensitivity somewhat, at an extremely large cost in speed.

--nohmm Turn off all HMM filter stages (F1 through F5b). The CYK filter, using QDBs, will be run on every full-length target sequence and will enforce a P-value threshold of 0.0001. Each subsequence that survives CYK will be passed to Inside, which will also use QDBs (but a looser set). This increases sensitivity somewhat, at a very large cost in speed.

--mid Turn off the HMM SSV and Viterbi filter stages (F1 through F2b). Set remaining HMM filter thresholds (F3 through F5b) to 0.02 by default, but changeable to <*x*> with **--Fmid** <*x*> sequence. This may increase sensitivity, at a significant cost in speed.

--default Use the default filtering strategy. This option is on by default. The filter thresholds are determined based on the database size.

--rfam Use a strict filtering strategy devised for large databases (more than 20 Gb). This will accelerate the search at a potential cost to sensitivity. It will have no effect if the database is larger than 20 Gb.

--hmmonly Only use the filter profile HMM for searches, do not use the CM. Only filter stages F1 through F3 will be executed, using strict P-value thresholds (0.02 for F1, 0.001 for F2 and 0.00001 for F3). Additionally a bias composition filter is used after the F1 stage (with P=0.02 survival threshold). Any hit that survives all stages and has an HMM E-value or bit score above the reporting threshold will be output. The user can change the HMM-only filter thresholds and options with **--hmmF1, --hmmF2, --hmmF3, --hmmnobias, --hmmnonull2,** and **--hmmmax.** By default, searches for any model with zero basepairs will be run in HMM-only mode. This can be turned off, forcing CM searches for these models with the **--nohmmonly** option. These options are only displayed if the **--devhelp** option is used.

--FZ <*x*> Set filter thresholds as the defaults used if the database were <**x**> megabases (Mb). If used with <**x**> greater than 20000 (20 Gb) this option has the same effect as **--rfam.**

--Fmid <*x*> With the **--mid** option set the HMM filter thresholds (F3 through F5b) to <*x*>. By default, <*x*> is 0.02.

Other Options

--notrunc Turn off truncated hit detection.

--anytrunc Allow truncated hits to begin and end at any position in a target sequence. By default, 5' truncated hits must include the first residue of their target sequence and 3' truncated hits must include the final residue of their target sequence. With this option you may observe fewer full length hits that extend to the beginning and end of the query CM.

--nonull3 Turn off the null3 CM score corrections for biased composition. This correction is not used during the HMM filter stages.

--mxsize <*x*> Set the maximum allowable CM DP matrix size to <*x*> megabytes. By default this size is 128 Mb. This should be large enough for the vast majority of searches, especially with smaller models. If **cmsearch** encounters an envelope in the CYK or Inside stage that requires a larger matrix, the envelope will be discounted from consideration. This behavior is like an additional filter that prevents expensive (slow) CM DP calculations, but at a potential cost to sensitivity. Note that if **cmsearch** is being run in <*n*> multiple threads on a multicore machine then each thread may have an allocated matrix of up to size <*x*> Mb at any given time.

--smxsize <*x*> Set the maximum allowable CM search DP matrix size to <*x*> megabytes. By default this size is 128 Mb. This option is only relevant if the CM will not use HMM banded matrices, i.e. if the **--max, --nohmm, --qdb, --fqdb, --nonbanded,** or **--fnonbanded** options are also used. Note that if **cmsearch** is being run in <*n*> multiple threads on a multicore machine then each thread may have an allocated matrix of up to size <*x*> Mb at any given time.

--cyk Use the CYK algorithm, not Inside, to determine the final score of all hits.

--acyk Use the CYK algorithm to align hits. By default, the Durbin/Holmes optimal accuracy algorithm is used, which finds the alignment that maximizes the expected accuracy of all aligned residues.

--wcx <*x*> For each CM, set the W parameter, the expected maximum length of a hit, to <*x*> times the consensus length of the model. By default, the W parameter is read from the CM file and was calculated based on the transition probabilities of the model by **cmbuild.** You can find out what the default W is for a model using **cmstat.** This option should be used with caution as it impacts the filtering pipeline at several different stages in nonobvious ways. It is only recommended for expert users searching for hits that are much longer than any of the homologs used to build the model in **cmbuild,** e.g. ones with large introns or other large insertions. This option cannot be used in combination with the **--nohmm, --fqdb** or **--qdb** options because in those cases W is limited by query-dependent bands.

--toponly Only search the top (Watson) strand of target sequences in <*seqdb*>*.* By default, both strands are searched. This will halve the database size (Z).

--bottomonly Only search the bottom (Crick) strand of target sequences in <*seqdb*>*.* By default, both strands are searched. This will halve the database size (Z).

--tformat <*s*> Assert that the target sequence database file is in format <*s*>*.* Accepted formats include *fasta, embl, genbank, ddbj, stockholm, pfam, a2m, afa, clustal,* and *phylip* The default is to autodetect the format of the file.

--cpu <*n*> Set the number of parallel worker threads to <*n*>*.* By default, Infernal sets this to the number of CPU cores it detects in your machine - that is, it tries to maximize the use of your available processor cores. Setting <*n*> higher than the number of available cores is of little if any value, but you may want to set it to something less. You can also control this number by setting an environment variable, *INFERNAL_NCPU.* This option is only available if Infernal was compiled with POSIX threads support. This is the default, but it may have been turned off at compile-time for your site or machine for some reason.

--stall For debugging the MPI master/worker version: pause after start, to enable the developer to attach debuggers to the running master and worker(s) processes. Send SIGCONT signal to release the pause. (Under gdb: *(gdb)* signal SIGCONT) (Only available if optional MPI support was enabled at compile-time.)

--mpi Run in MPI master/worker mode, using *mpirun.* To use **--mpi,** the sequence file must have first been 'indexed' using the **esl-sfetch** program, which is included with Infernal,

in the *easel/miniapps/* subdirectory. (Only available if optional MPI support was enabled at compile-time.)

`cmstat` - summary statistics for a covariance model file

Synopsis

cmstat *[options]* <*cmfile*>

Description

The **cmstat** utility prints out a tabular file of summary statistics for each covariance model in <*cmfile*>.

<*cmfile*> may be '-' (a dash character), in which case CMs are read from a <stdin> pipe instead of from a file.

By default, **cmstat** prints general statistics of the model and the alignment it was built from, one line per model in a tabular format. The columns are:

idx The index of this profile, numbering each on in the file starting from 1.

name The name of the profile.

accession The optional accession of the profile, or "-" if there is none.

nseq The number of sequences that the profile was estimated from.

eff_nseq The effective number of sequences that the profile was estimated from, after Infernal applied an effective sequence number calculation such as the default entropy weighting.

clen The length of the model in consensus residues (match states).

W The expected maximum length of a hit to the model.

bps The number of basepairs in the model.

bifs The number of bifurcations in the model.

model What type of model will be used by default in **cmsearch** and **cmscan** for this profile, either "cm" or "hmm". For profiles with 0 basepairs, this will be "hmm" (unless the **--nohmmonly** option is used). For all other profiles, this will be "cm".

rel entropy, cm: Mean relative entropy per match state, in bits. This is the expected (mean) score per consensus position. This is what the default entropy-weighting method for effective sequence number estimation focuses on, so for default Infernal, this value will often reflect the default target for entropy-weighting. If the "model" field for this profile is "hmm", this field will be "-".

rel entropy, hmm: Mean relative entropy per match state, in bits, if the CM were transformed into an HMM (information from structure is ignored). The larger the difference between the CM and HMM relative entropy, the more the model will rely on structural conservation relative sequence conservation when identifying homologs.

If the model(s) in <*cmfile*> have been calibrated with **cmcalibrate** the **-E, -T,** and **-Z** <*n*> options can be used to invoke an alternative output mode, reporting E-values and corresponding bit scores for a specified database size of <*n*> megabases (Mb). If the model(s) have been calibrated and include Rfam GA, TC, and/or NC bit score thresholds the **--cut_ga, --cut_tc,** and/or **--cut_nc** options can be used to display E-values that correspond to the bit score thresholds. Seperate bit scores or E-values will be displayed for each of the four possible CM search algorithm and model configuration pairs: local Inside, local CYK, glocal Inside and glocal CYK.

For profiles with zero basepairs (those with "hmm" in the "model" field), any E-value and bit score statistics will pertain to the profile HMM filter, instead of to the CM. This is also true for all profiles if the **--hmmonly** option is used.

Options

-h Help; print a brief reminder of command line usage and all available options.

-E <*x1*> Report bit scores that correspond to an E-value of <*x1*> in a database of <*x*> megabases (Mb), where <*x*> is 10 by default but settable with the **-Z** <*x*> option.

-T *<x1>* Report E-values that correspond to a bit score of *<x1>* in a database of *<x>* megabases (Mb), where *<x>* is 10 by default but settable with the **-Z** *<x>* option.

-Z *<x>* With the **-E, -T, --cut_ga, --cut_nc,** and **--cut_tc** options, calculate E-values as if the target database size was *<x>* megabases (Mb). By default, *<x>* is 10.

--cut_ga Report E-values that correspond to the GA (Rfam gathering threshold) bit score in a database of *<x>* megabases (Mb), where *<x>* is 10 by default but settable with the **-Z** *<x>* option.

--cut_tc Report E-values that correspond to the TC (Rfam trusted cutoff) bit score in a database of *<x>* megabases (Mb), where *<x>* is 10 by default but settable with the **-Z** *<x>* option.

--cut_nc Report E-values that correspond to the NC (Rfam noise cutoff) bit score in a database of *<x>* megabases (Mb), where *<x>* is 10 by default but settable with the **-Z** *<x>* option.

--key *<s>* Only print statistics for CM with name or accession *<s>,* skip all other models in *<cmfile>.*

--hmmonly Print statistics on the profile HMM filters for all profiles, instead of the CMs. This can be useful if you plan to use the **--hmmonly** option to **cmsearch** or **cmscan.**

--nohmmonly Always print statistics on the CM for each profile, even for those with zero basepairs.

9 File and output formats

Infernal CM files

The file `tutorial/Cobalamin.c.cm` gives an example of an Infernal ASCII CM save file. An abridged version is shown here, where (. . .) mark deletions made for clarity and space:

```
INFERNAL1/a [1.1 | June 2012]
NAME     Cobalamin
ACC      RF00174
DESC     Cobalamin riboswitch
STATES   592
NODES    163
CLEN     191
W        460
ALPH     RNA
RF       no
CONS     yes
MAP      yes
DATE     Wed Jun 13 05:40:07 2012
COM      [1] ./cmbuild Cobalamin.cm ../tutorial/Cobalamin.sto
COM      [2] ./cmcalibrate Cobalamin.cm
PBEGIN   0.05
PEND     0.05
WBETA    1e-07
QDBBETA1 1e-07
QDBBETA2 1e-15
N2OMEGA  1.52588e-05
N3OMEGA  1.52588e-05
ELSELF   -0.08926734
NSEQ     431
EFFN     6.652168
CKSUM    2307274568
NULL     0.000  0.000  0.000  0.000
GA       39.00
TC       39.00
NC       38.79
EFP7GF   -9.3826 0.71319
ECMLC    0.69050    -9.55632    -0.82028     1600000      499982  0.002400
ECMGC    0.33713   -30.56949   -21.45119     1600000        8652  0.046232
ECMLI    0.68481    -7.98572     0.30786     1600000      351369  0.003415
ECMGI    0.38286   -21.23885   -13.16656     1600000        8796  0.045475
CM
                                             [ ROOT    0 ]      -      - - - - -
     S     0    -1 0     1     4     0     1   460   771  -8.175  -8.382  -0.025  -6.528
    IL     1     1 2     1     4    86   133   462   774  -1.686  -2.369  -1.117  -4.855                 0.000  0.000  0.000  0.000
    IR     2     2 3     2     3    86   133   462   774  -1.442  -0.798  -4.142                         0.000  0.000  0.000  0.000
                                             [ MATL    1 ]      1      - u - - -
    ML     3     2 3     5     3    86   132   461   772  -9.129  -0.009  -7.783                         0.192 -0.324 -0.320  0.331
     D     4     2 3     5     3    80   128   458   769  -6.226  -1.577  -0.618
    IL     5     5 3     5     3    85   132   461   773  -1.442  -0.798  -4.142                         0.000  0.000  0.000  0.000
(...)
                                             [ MATL   98 ]    151      - C - - -
    ML   588   587 3   590     2     1     1     1     1       *   0.000                                -3.022  1.825 -3.061 -2.226
     D   589   587 3   590     2     0     0     0     0       *   0.000
    IL   590   590 3   590     2     1     1    13    28  -1.823  -0.479                                 0.000  0.000  0.000  0.000
                                             [ END    99 ]      -      - - - - -
     E   591   590 3    -1     0     0     0     0     0
//
HMMER3/f [i1.1 | June 2012]
NAME  Cobalamin
ACC   RF00174
DESC  Cobalamin riboswitch
LENG  191
MAXL  565
ALPH  RNA
RF    no
MM    no
CONS  yes
CS    yes
MAP   yes
DATE  Wed Jun 13 05:40:08 2012
COM   [1] ./cmbuild Cobalamin.cm ../tutorial/Cobalamin.sto
NSEQ  431
EFFN  4.955421
CKSUM 2307274568
STATS LOCAL MSV       -10.2356  0.71319
STATS LOCAL VITERBI   -12.2484  0.71319
STATS LOCAL FORWARD    -3.9056  0.71319
HMM          A        C        G        U
            m->m     m->i     m->d     i->m     i->i     d->m     d->d
  COMPO   1.37169  1.39466  1.27962  1.51293
          1.38629  1.38629  1.38629  1.38629
          0.02747  4.30141  4.30141  1.46634  0.26236  0.00000        *
      1   1.24903  1.60847  1.61442  1.15831      1 u - - :
          1.38629  1.38629  1.38629  1.38629
          0.02747  4.30141  4.30141  1.46634  0.26236  1.09861  0.40547
(...)
    191   1.51542  1.17791  1.56046  1.33817    441 c - - :
          1.38629  1.38629  1.38629  1.38629
          0.01381  4.28939        *  1.46634  0.26236  0.00000        *
//
```

A CM file consists of one or more CMs and associated filter HMMs. Each CM is immediately followed by its filter HMM, this is mandatory. Each CM starts with a format version identifier (here, `INFERNAL1/a`) and ends with `//` on a line by itself. Each HMM also starts with a format version identifier (here, `HMMER3/f`) and ends with `//` on a line by

itself. The format version identifier allows backward compatibility as the Infernal software evolves: it tells the parser this file is from Infernal's save file format version a. The closing // allows Infernal to determine when a CM ends and its profile HMM begins, and allows multiple CM/filter HMM pairs to be concatenated together into a single file.

The CM format is divided into two regions. The first region contains textual information and miscalleneous parameters in a roughly tag-value scheme. This section ends with a line beginning with the keyword CM. The second region is a tabular, whitespace-limited format for the main model parameters.

All emission and transition model parameters are stored as log-odds scores in bits with three digits of precision to the right of the decimal point, rounded. The special case of a score of infinity, corresponding to an impossible emission or transition, is stored as '*'.

Spacing is arranged for human readability, but the parser only cares that fields are separated by at least one space character.

The CM format is described in more detail below, followed by a description of the HMMER3 HMM format for the CM's mandatory filter HMM filter.

CM header section

The header section is parsed line by line in a tag/value format. Each line type is either **mandatory** or **optional** as indicated.

INFERNAL1/a Unique identifier for the save file format version; the /a means that this is INFERNAL1 CM file format version a. When INFERNAL changes its save file format, the revision code advances. This way, parsers may easily remain backwards compatible. The remainder of the line after the INFERNAL1/a tag is free text that is ignored by the parser. INFERNAL currently writes its version number and release date in brackets here, e.g. [1.1 | June 2012] in this example. **Mandatory.**

NAME <s> Model name; <s> is a single word containing no spaces or tabs. The name is normally picked up from the #=GF ID line from a Stockholm alignment file. If this is not present, the name is created from the name of the alignment file by removing any file type suffix. For example, an otherwise nameless CM built from the alignment file tRNA.sto would be named tRNA. **Mandatory.**

ACC <s> Accession number; <s> is a one-word accession number. This is picked up from the #=GF AC line in a Stockholm format alignment. **Optional.**

DESC <s> Description line; <s> is a one-line free text description. This is picked up from the #=GF DE line in a Stockholm alignment file. **Optional.**

STATES <d> Number of states; <d>, a positive nonzero integer, is the number of states in the model. **Mandatory.**

CLEN <d> Consensus model length; <d>, a positive nonzero integer, is the number of consensus positions in the model, which equals the number of MATL nodes plus the number of MATR nodes plus two times the number of MATP nodes. **Mandatory.**

W <d> Window length; <d>, a positive nonzero integer, is the length in residues of the maximum expected size of a hit to this model. This is calculated based on the transition probabilities of the model* **Mandatory.**

ALPH <s> Symbol alphabet type. Currently this will necessarily be RNA for RNA sequence analysis models. The symbol alphabet size K is set to 4 and the symbol alphabet to "ACGU". **Mandatory.**

RF <s> Reference annotation flag; <s> is either no or yes (case insensitive). If yes, the reference annotation character field(s) for each match state in the main model (see below) is valid; if no, these characters are ignored. Reference column annotation is picked up from a Stockholm alignment file's #=GC RF line. by cmbuild. It is propagated to alignment outputs, and also may optionally be used to define consensus match columns in CM construction. **Optional**; assumed to be no if not present.

CONS <s> Consensus residue annotation flag; <s> is either no or yes (case insensitive). If yes, the consensus residue field(s) for each match state in the main model (see below) is valid. If no, these characters are ignored. Consensus residue annotation is determined when models are built. For models of single sequences, the consensus is the same as the query sequence. For models of multiple alignments, the consensus is the highest scoring residue or basepair for each match state. Upper case MATL_ML and MATR_MR (single stranded) residues indicate that the model emission's score for the consensus

*Specifically, W is set as the *dmax* value for the ROOT_S state (state 0) from the QDB algorithm using β equal to the WBETA value (Nawrocki and Eddy, 2007).

residue is $\geq$ to 1.0 bit. Upper case MATP_MP basepairs indicate that the model emission's score for the consensus basepair is $\geq$ to 3.0 bits.

MAP <s> Map annotation flag; `<s>` is either `no` or `yes` (case insensitive). If set to `yes`, the map annotation field in the main model (see below) is valid; if `no`, that field will be ignored. The CM/alignment map annotates each match state with the index of the alignment column from which it came. It can be used for quickly mapping any subsequent CM alignment back to the original multiple alignment, via the model. **Optional**; assumed to be no if not present.

DATE <s> Date the model was constructed; `<s>` is a free text date string. This field is only used for logging purposes.[†] **Optional.**

COM [<n>] <s> Command line log; `<n>` counts command line numbers, and `<s>` is a one-line command. There may be more than one `COM` line per save file, each numbered starting from $n = 1$. These lines record every Infernal command that modified the save file. This helps us reproducibly and automatically log how Rfam models have been constructed, for example. **Optional.**

PBEGIN <f> Local begin probability; The aggregate probability of a local begin into any internal entry state is `<f>`. The probability of a local begin into any single internal entry state is `<f>` divided by the number of internal entry states in the model. All MATP_MP, MATL_ML, MATR_MR, and BIF_B states, except for any in the the second node of the model (first non-ROOT node), are internal entry states. The local begin probability does not affect any of the emission/transition parameters in the CM file, which correspond to the CM in *global* search/alignment mode, but it does affect the calibration of E-value parameters for local search by `cmcalibrate`. `cmsearch` and `cmscan` therefore need to read this probability from the CM file in order to use the same local begin probabilities used during calibration and report appropriate E-values. **Optional**; assumed to be 0.05 if not present.

PEND <f> Local end probability; The aggregate probability of a local end out of any internal exit state is `<f>`. The probability of a local end out of any single internal entry state is `<f>` divided by the number of internal exit states in the model. All MATP_MP, MATL_ML, MATR_MR, BEGL_S, and BEGR_S states, except for any for which the following node is an END node, are internal exit states. The local end probability does not affect any of the emission/transition parameters in the CM file, which correspond to the CM in *global* search/alignment mode, but it does affect the calibration of E-value parameters for local search by `cmcalibrate`. `cmsearch` and `cmscan` therefore need to read this probability from the CM file in order to use the same local end probabilities used during calibration and report appropriate E-values. **Optional**; assumed to be 0.05 if not present.

WBETA <f> Tail loss probability for calculating window length (W); The QDB algorithm (Nawrocki and Eddy, 2007) was used to determine the maximum expected length of a hit (W) using a tail loss probability of `<f>`, W was set as the *dmax* value for the ROOT_S state (state 0). **Mandatory.**

QDBBETA1 <f> Tail loss probability for calculating the tighter of the two sets of query-dependent bands (QDBs); The QDB algorithm (Nawrocki and Eddy, 2007) was used to determine the minimum and maximum subsequence lengths allowed to align to the subtree rooted at each state of the model, using a tail loss β probability of `<f>`. These minimum and maximum values for each state are included in each state line in the main model section, described below. below. The `<f>` value for `QDBBETA2` will be less than or equal to the `<f>` value for `QDBBETA1`. **Mandatory.**

QDBBETA2 <f> Tail loss probability for calculating the looser of the two sets of query-dependent bands (QDBs); The QDB algorithm (Nawrocki and Eddy, 2007) was used to determine the minimum and maximum subsequence lengths allowed to align to the subtree rooted at each state of the model, using a tail loss β probability of `<f>`. These minimum and maximum values for each state are included in each state line in the main model section, described below. The `<f>` value for `QDBBETA2` will be less than or equal to the `<f>` value for `QDBBETA1`. **Mandatory.**

N2OMEGA <f> The prior probability for the alternative "null2" model for biased composition sequences. **Mandatory**; but only relevant in `cmsearch` and `cmscan` if the `--null2` option is used.

N3OMEGA <f> The prior probability for the alternative "null3" model for biased composition sequences. **Mandatory**; the null3 model is used by default in `cmcalibrate`, `cmsearch` and `cmscan`.

[†]Infernal does not use dates for any purpose other than human-readable annotation, so it is no more prone than you are to Y2K, Y2038, or any other date-related eschatology.

NSEQ <d> Sequence number; `<d>` is a nonzero positive integer, the number of sequences that the CM was trained on, i.e. the number of sequences in the input alignment used to create the CM in `cmbuild`. This field is only used for logging purposes. **Optional.**

EFFN <f> Effective sequence number; `<f>` is a nonzero positive real, the effective total number of sequences determined by `cmbuild` during sequence weighting, for combining observed counts with Dirichlet prior information in parameterizing the model. This field is only used for logging purposes. **Optional.**

CKSUM <d> Training alignment checksum; `<d>` is a nonnegative unsigned 32-bit integer. This number is calculated from the training sequence data, and used in conjunction with the alignment map information to verify that a given alignment is indeed the alignment that the map is for. **Optional.**

NULL <f> <f> <f> <f> null model emission scores for each alphabet symbol. By default, these are all `0.0` but may not be if the `--null` option was used in `cmbuild` to define alternative null model scores. Because only RNA CMs can be built by `cmbuild` there will be four values on this line, one each for "A", "C", "G", and "U". **Mandatory.**

GA <f> Rfam GA gathering threshold bit score. The GA bit score threshold is normally picked up from the `#=GF GA` line from a Stockholm alignment file. GA thresholds are generally considered to be the reliable curated thresholds defining family membership; for example, in Rfam, these thresholds define what gets included in Rfam Full alignments based on searches with Rfam Seed models. **Optional.**

NC <f> Rfam NC noise cutoff bit score. The NC bit score threshold is normally picked up from the `#=GF NC` line from a Stockholm alignment file. NC thresholds are generally considered to be the score of the highest-scoring known false positive found by searches during preparation of the Rfam database. **Optional.**

TC <f> Rfam TC trusted cutoff bit score. The TC bit score threshold is normally picked up from the `#=GF TC` line from a Stockholm alignment file. TC thresholds are generally considered to be the score of the lowest scoring believed true positive that is above all known false positives found by searches during preparation of the Rfam database. **Optional.**

EFP7GF <f1> <f2> Statistical parameters for filter HMM E-value calculations in glocal mode for the Forward algorithm. `<f1>` and `<f2>` are τ and λ for exponential tails for glocal Forward filter HMM scores. This line is necessary in the CM file section rather than the filter HMM file because glocal HMM searches are not normally performed in HMMER3, but are part of the HMM filter pipeline in Infernal. **Mandatory.**

ECMLC <f1> <f2> <f3> <d1> <d2> <f4> Statistical parameters needed for E-value calculations for the CM CYK algorithm in local mode. This line, along with the next three, with tags `ECMGC`, `ECMLI`, and `ECMGI`, must either all be present or none of them must be present. If present, the model is considered calibrated for E-value statistics. These lines will not be present in a model after it is created by `cmbuild` but will be added by the `cmcalibrate` program. `<f1>` and `<f2>` are λ and τ, the slope and location parameters for exponential tails for local CYK scores. λ values must be positive. The remaining values were computed in `cmcalibrate` during model calibration: `<f3>` is a different τ value computed for the full histogram of all hits; `<d1>` is the database size in residues; `<d2>` is the total number of non-overlapping hits of any score found; `<f4>` is the fraction of the high-scoring histogram tail fit to an exponential tail. Of these parameters, only `<f1>`, `<f2>` and `<d2>` are used, the others are only stored in the CM file for record keeping purposes.

ECMGC <f1> <f2> <f3> <d1> <d2> <f4> Statistical parameters analogous to those described above in the `ECMLC` line, except that these pertain to CM CYK scores in glocal mode.

ECMLI <f1> <f2> <f3> <d1> <d2> <f4> Statistical parameters analogous to those described above in the `ECMLC` line, except that these pertain to CM Inside scores in local mode.

ECMGI <f1> <f2> <f3> <d1> <d2> <f4> Statistical parameters analogous to those described above in the `ECMLC` line, except that these pertain to CM Inside scores in glocal mode.

CM Flags the start of the main model section. **Mandatory.**

CM main model section

All the remaining fields are **mandatory**.

The model section consistes of two types of lines: node lines and state lines. Each node line is immediately followed by one or more state lines, one each for each state within the node.

Node line Each node line begins with 45 spaces, and includes ten fields.

The first field is always a `[` character.

The second is the node type, one of ROOT, MATP, MATL, MATR, BIF, BEGL, BEGR, or END.

The next field is the index of the node in the model, greater than or equal to 0. Node indices are not always in increasing order, e.g. node 200 may come on a line before node 100.

The fourth field is always a `]` character.

The next two fields are the `MAP` annotation for this node. If `MAP` was `yes` in the header and the node is a MATP (match pair) node, then these fields will both be positive integers, representing the alignment column indices for the left and right halves of this match pair state, respectively. If `MAP` was `yes` and the node is a MATL (match left) node, then the first field will be the alignment column for this match state and the second field will be '-'. If `MAP` was `yes` and the node is a MATR (match right) node, then the first field will be '-' and the second field will be the alignment column for this match state. If the node is any other type, or if the `MAP` was `no` in the header, then both fields will be '-'.

The next two fields are the `CONS` consensus residue(s) for this node. If `CONS` was `yes` in the header and the node is a MATP (match pair) node, then these fields will both be characters, the consensus residues for the left and right halves of this match pair state, respectively. If `CONS` was `yes` and the node is a MATL (match left) node, then the first field will be the consensus residue for this state and the second field will be '-'. If `CONS` was `yes` and the node is a MATR (match right) node, then the first field will be '-' and the second field will be the consensus residue for this state. If the node is any other type, or if the `CONS` was `no` in the header, then both fields will be '-'.

The final two fields are the `RF` annotation for this node. this node. If `RF` was `yes` in the header and the node is a MATP (match pair) node, then these fields will both be characters, the reference annotation character for the left and right halves of this match pair state, respectively. If `RF` was `yes` and the node is a MATL (match left) node, then the first field will be the reference annotation character for this state and the second field will be '-'. If `RF` was `yes` and the node is a MATR (match right) node, then the first field will be '-' and the second field will be the reference annotation character for this state. If the node is any other type, or if the `CONS` was `no` in the header, then both fields will be '-'.

Each node line is followed by 1 to 6 state lines depending on the node type. ROOT, MATL, and MATR node lines are followed by 3 state lines. BIF, BEGL, and END nodes are followed by 1 state line. BEGLR node lines have 2 state lines after them, and MATP node lines are followed by 6 state lines.

State line The number of fields on a state line is variable depending on the state type and the number of possible transitions from the state. The first field is the state type, either "MP", "ML", "MR", "IL", "IR", "D", "B", "S", or "E".

The next field is the state index, these are in increasing order starting with 0 (i.e. lower numbered states always occur earlier in the file than higher numbered ones).

The next field is the index of the highest numbered "parent" state for the current state, where state a is a parent of state b if state a can transition to state b.

The next field is the number of parent states for the current state. A set of parent states are always contiguously numbered. For example, if state a is the highest numbered parent state of b and b has 3 parent states, then $a-2$, $a-1$, and a are the three parent states of b.

The next field is the index of the lowest numbered "child" state for the current state, where state c is a child of state b if b can transition to state c.

The next field is the number of child states for the current state. A set of child states are always contiguously numbered. For example, if state c is the lowest numbered parent state of b and b has 3 parent states, then c, $c+1$, and $c+2$ are the three child states of b. As a special case, for "B" (bifurcation) states this field is the state index of the "BEGR_S" state to which the "B" state necessarily transitions with probability 1.0.

The next four fields <n1>, <n2>, <n3>, and <n4> are query dependent band values for the current state. These are integers. <n1> is the minimum expected subsequence length to align at the subtree rooted at this state calculated with the QDB algorithm (Nawrocki and Eddy, 2007) using a β tail loss probability value given in the header in the `QDBBETA2` line. <n2> is the same, but calculated with β equal to the value from the `QDBBETA1` header line. <n3> is the maximum expected subsequence length to align at the subtree rooted at this state calculated with the QDB algorithm (Nawrocki and Eddy, 2007) using a β tail loss probability value given in the header in the `QDBBETA1` line. <n4> is the same, but calculated with β equal to the value from the `QDBBETA2` header line. These values should be in increasing order: $< n1 > \leq < n2 > \leq < n3 > \leq < n4 >$,

although Infernal does not enforce this to be true. The QDB values will only be used by `cmsearch` and `cmscan` if certain option combinations are used (see the manual page for those programs); by default they are not used.

After the four QDB values, the next set of fields are log-odds bit scores for possible transitions out of this state to all child states of the current states. The number of child states is given earlier on the line as the sixth field. It varies depending on the state type and the node type of the *next* node in the model. For a list of all possible sets of transitions for each possible state type/next node combination see Table 1 of (Nawrocki and Eddy, 2007). As a special case, "B" (bifurcation) states have zero transition score fields, they necessarily transition to their child "BEGL_S" and "BEGR_S" states with a probability of 1.0 (score of 0 bits).

After the transition scores are the emission scores. "MP" state lines have 16 emission log-odds bit scores. All other types of emitting states ("ML", "MR", "IL", "IR") will have four emission scores. All other types of states will have no emission scores. For "MP" states, the sixteen scores are for the sixteen possible non-degenerate RNA basepairs: "AA", "AC", "AG", "AU", "CA", "CC", "CG", "CU", "GA", "GC", "GG", "GU", "UA", "UC", "UG", "UU", in that order. For the other emitting states the four scores are for "A", "C", "G", and "U", in that order.

Finally, the last line of the format is the "//" record separator. After the CM comes its associated filter HMM in HMMER3 format, described below.

HMMER3 filter HMM format

As with the CM, the HMM format is divided into two regions. The first region contains textual information and miscalleneous parameters in a roughly tag-value scheme. This section ends with a line beginning with the keyword `HMM`. The second region is a tabular, whitespace-limited format for the main model parameters.

All HMM probability parameters are all stored as negative natural log probabilities with five digits of precision to the right of the decimal point, rounded. For example, a probability of 0.25 is stored as $-\log 0.25 = 1.38629$. The special case of a zero probability is stored as '*'.

Spacing is arranged for human readability, but the parser only cares that fields are separated by at least one space character.

A more detailed description of the format follows[‡].

HMM header section

The header section is parsed line by line in a tag/value format. Each line type is either **mandatory** or **optional** as indicated.

HMMER3/f Unique identifier for the save file format version; the `/b` means that this is HMMER3 HMM file format version b. When HMMER3 changes its save file format, the revision code advances. This way, parsers may easily remain backwards compatible. The remainder of the line after the `HMMER3/b` tag is free text that is ignored by the parser. HMMER currently writes its version number and release date in brackets here, e.g. `[3.0b2 | June 2009]` in this example. **Mandatory.**

NAME <s> Model name; `<s>` is a single word containing no spaces or tabs. The name is normally picked up from the `#=GF ID` line from a Stockholm alignment file. If this is not present, the name is created from the name of the alignment file by removing any file type suffix. For example, an otherwise nameless HMM built from the alignment file `rrm.slx` would be named `rrm`. **Mandatory.**

ACC <s> Accession number; `<s>` is a one-word accession number. This is picked up from the `#=GF AC` line in a Stockholm format alignment. **Optional.**

DESC <s> Description line; `<s>` is a one-line free text description. This is picked up from the `#=GF DE` line in a Stockholm alignment file. **Optional.**

LENG <d> Model length; `<d>`, a positive nonzero integer, is the number of match states in the model. **Mandatory.**

[‡]This section is nearly identical to one from the HMMER3 user's guide Eddy (2009). It is included here, instead of just providing reference to the HMMER guide, for convenience.

ALPH <s> Symbol alphabet type. For biosequence analysis models, <s> is `amino`, `DNA`, or `RNA` (case insensitive). There are also other accepted alphabets for purposes beyond biosequence analysis, including `coins`, `dice`, and `custom`. This determines the symbol alphabet and the size of the symbol emission probability distributions. If `amino`, the alphabet size K is set to 20 and the symbol alphabet to "ACDEFGHIKLMNPQRSTVWY" (alphabetic order); if `DNA`, the alphabet size K is set to 4 and the symbol alphabet to "ACGT"; if `RNA`, the alphabet size K is set to 4 and the symbol alphabet to "ACGU". **Mandatory.**

RF <s> Reference annotation flag; <s> is either `no` or `yes` (case insensitive). If `yes`, the reference annotation character field for each match state in the main model (see below) is valid; if `no`, these characters are ignored. Reference column annotation is picked up from a Stockholm alignment file's `#=GC RF` line. It is propagated to alignment outputs, and also may optionally be used to define consensus match columns in profile HMM construction. **Optional**; assumed to be no if not present.

CONS <s> Consensus residue annotation flag; <s> is either `no` or `yes` (case insensitive). If `yes`, the consensus residue field for each match state in the main model (see below) is valid. If `no`, these characters are ignored. Consensus residue annotation is determined when models are built. For models of single sequences, the consensus is the same as the query sequence. For models of multiple alignments, the consensus is the maximum likelihood residue at each position. Upper case indicates that the model's emission probability for the consensus residue is $\geq$ an arbitrary threshold (0.5 for protein models, 0.9 for DNA/RNA models).

CS <s> Consensus structure annotation flag; <s> is either `no` or `yes` (case insensitive). If `yes`, the consensus structure character field for each match state in the main model (see below) is valid; if `no` these characters are ignored. Consensus structure annotation is picked up from a Stockholm file's `#=GC SS_cons` line, and propagated to alignment displays. **Optional**; assumed to be no if not present.

MAP <s> Map annotation flag; <s> is either `no` or `yes` (case insensitive). If set to `yes`, the map annotation field in the main model (see below) is valid; if `no`, that field will be ignored. The HMM/alignment map annotates each match state with the index of the alignment column from which it came. It can be used for quickly mapping any subsequent HMM alignment back to the original multiple alignment, via the model. **Optional**; assumed to be no if not present.

DATE <s> Date the model was constructed; <s> is a free text date string. This field is only used for logging purposes. **Optional.**

COM [<n>] <s> Command line log; <n> counts command line numbers, and <s> is a one-line command. There may be more than one `COM` line per save file, each numbered starting from $n = 1$. These lines record every HMMER command that modified the save file. This helps us reproducibly and automatically log how Pfam models have been constructed, for example. **Optional.**

NSEQ <d> Sequence number; <d> is a nonzero positive integer, the number of sequences that the HMM was trained on. This field is only used for logging purposes. **Optional.**

EFFN <f> Effective sequence number; <f> is a nonzero positive real, the effective total number of sequences determined by `hmmbuild` during sequence weighting, for combining observed counts with Dirichlet prior information in parameterizing the model. This field is only used for logging purposes. **Optional.**

CKSUM <d> Training alignment checksum; <d> is a nonnegative unsigned 32-bit integer. This number is calculated from the training sequence data, and used in conjunction with the alignment map information to verify that a given alignment is indeed the alignment that the map is for. **Optional.**

STATS <s1> <s2> <f1> <f2> Statistical parameters needed for E-value calculations. <s1> is the model's alignment mode configuration: currently only `LOCAL` is recognized. <s2> is the name of the score distribution: currently `MSV`, `VITERBI`, and `FORWARD` are recognized. <f1> and <f2> are two real-valued parameters controlling location and slope of each distribution, respectively;

μ and λ for Gumbel distributions for MSV and Viterbi scores, and τ and λ for exponential tails for Forward scores. λ values must be positive. All three lines or none of them must be present: when all three are present, the model is considered to be calibrated for E-value statistics. **Optional.**

HMM Flags the start of the main model section. Solely for human readability of the tabular model data, the symbol alphabet is shown on the `HMM` line, aligned to the fields of the match and insert symbol emission distributions in the main model below. The next line is also for human readability, providing column headers for the state transition probability fields in the main model section that follows. Though unparsed after the `HMM` tag, the presence of two header lines is **mandatory:** the parser always skips the line after the `HMM` tag line.

COMPO <f>*K The first line in the main model section may be an optional line starting with **COMPO**: these are the model's overall average match state emission probabilities, which are used as a background residue composition in the "filter null" model. The K fields on this line are log probabilities for each residue in the appropriate biosequence alphabet's order. **Optional.**

HMM main model section

All the remaining fields are **mandatory**.

The first two lines in the main model section are atypical.[§] They contain information for the core model's BEGIN node. This is stored as model node 0, and match state 0 is treated as the BEGIN state. The begin state is mute, so there are no match emission probabilities. The first line is the insert 0 emissions. The second line contains the transitions from the begin state and insert state 0. These seven numbers are: $B \rightarrow M_1$, $B \rightarrow I_0$, $B \rightarrow D_1$; $I_0 \rightarrow M_1$, $I_0 \rightarrow I_0$; then a 0.0 and a '*', because by convention, nonexistent transitions from the nonexistent delete state 0 are set to $\log 1 = 0$ and $\log 0 = -\infty =$ '*'.

The remainder of the model has three lines per node, for M nodes (where M is the number of match states, as given by the `LENG` line). These three lines are (K is the alphabet size in residues):

Match emission line The first field is the node number ($1 \dots M$). The parser verifies this number as a consistency check (it expects the nodes to come in order). The next K numbers for match emissions, one per symbol, in alphabetic order.

The next field is the `MAP` annotation for this node. If `MAP` was `yes` in the header, then this is an integer, representing the alignment column index for this match state (1..alen); otherwise, this field is '-'.

The next field is the `CONS` consensus residue for this node. If `CONS` was `yes` in the header, then this is a single character, representing the consensus residue annotation for this match state; otherwise, this field is '-'.

The next field is the `RF` annotation for this node. If `RF` was `yes` in the header, then this is a single character, representing the reference annotation for this match state; otherwise, this field is '-'.

The next field is the `CS` annotation for this node. If `CS` was `yes`, then this is a single character, representing the consensus structure at this match state; otherwise this field is '-'.

Insert emission line The K fields on this line are the insert emission scores, one per symbol, in alphabetic order.

State transition line The seven fields on this line are the transitions for node k, in the order shown by the transition header line: $M_k \rightarrow M_{k+1}, I_k, D_{k+1}$; $I_k \rightarrow M_{k+1}, I_k$; $D_k \rightarrow M_{k+1}, D_{k+1}$.

For transitions from the final node M, match state $M+1$ is interpreted as the END state E, and there is no delete state $M+1$; therefore the final $M_k \rightarrow D_{k+1}$ and $D_k \rightarrow D_{k+1}$ transitions are always * (zero probability), and the final $D_k \rightarrow M_{k+1}$ transition is always 0.0 (probability 1.0).

Finally, the last line of the format is the "//" record separator.

[§] That is, the first two lines after the optional COMPO line. Don't be confused by the presence of an optional COMPO line here. The COMPO line is placed in the model section, below the residue column headers, because it's an array of numbers much like residue scores, but it's not really part of the model.

RNA secondary structures: WUSS notation

Infernal annotates RNA secondary structures using a linear string representation called “WUSS notation” (Washington University Secondary Structure notation).

The symbology is extended from the common bracket notation for RNA secondary structures, where open- and close-bracket symbols (or parentheses) are used to annotate base pairing partners: for example, `((((...))))` indicates a four-base stem with a three-base loop. Bracket notation is difficult for humans to interpret, for anything much larger than a simple stem-loop. WUSS notation makes it somewhat easier to interpret the annotation for larger structures.

The following figure shows an example with the key elements of WUSS notation. At the top left is an example RNA structure. At the top right is the same structure, with different RNA structural elements marked. Below both structure pictures : the WUSS notation string for the structure.

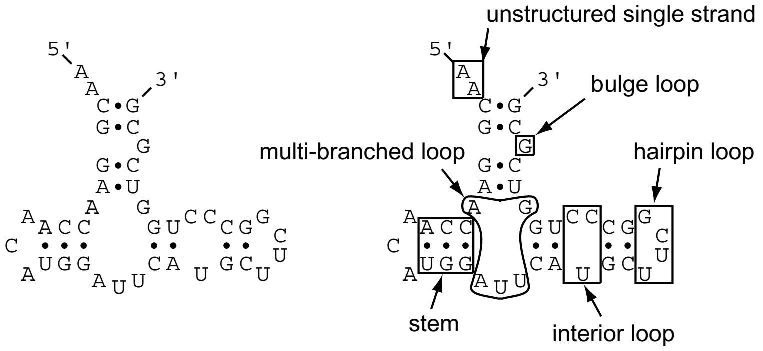


```
::((((,<<<___>>>,,,<<-<<____>>-->>,))-))
AACGGAACCAACAUGGAUUCAUGCUUCGGCCCUGGUCGCG
```

Full (output) WUSS notation

In detail, symbols used by WUSS notation in *output* structure annotation strings are as follows:

Base pairs Base pairs are annotated by nested matching pairs of symbols `<>`, `()`, `[]`, or `{}`. The different symbols indicate the “depth” of the helix in the RNA structure as follows: `<>` are used for simple terminal stems; `()` are used for “internal” helices enclosing a multifurcation of all terminal stems; `[]` are used for internal helices enclosing a multifurcation that includes at least one annotated `()` stem already; and `{}` are used for all internal helices enclosing deeper multifurcations.

Hairpin loops Hairpin loop residues are indicated by underscores, `_`. Simple stem loops stand out as, e.g. `<<<<____>>>>`.

Bulge, interior loops Bulge and interior loop residues are indicated by dashes, `-`.

Multifurcation loops Multifurcation loop residues are indicated by commas, `,`. The mnemonic is “stem 1, stem2”, e.g. `<<<___>>>,,<<<___>>>`.

External residues Unstructured single stranded residues completely outside the structure (unenclosed by any base pairs) are annotated by colons, `:`.

Insertions Insertions relative to a known structure are indicated by periods, `.`. Regions where local structural alignment was invoked, leaving regions of both target and query sequence unaligned, are indicated by tildes, `~`. These symbols only appear in alignments of a known (query) structure annotation to a target sequence of unknown structure.

Pseudoknots WUSS notation allows pseudoknots to be annotated as pairs of upper case/lower case letters: for example, `<<<<_AAAA____>>>>aaaa` annotates a simple pseudoknot; additional pseudoknotted stems could be annotated by `Bb`, `Cc`, etc. Infernal cannot handle pseudoknots, however; pseudoknot notation never appears in Infernal output; it is accepted in input files, but ignored.

An example of WUSS notation for a complicated structure (*E. coli* RNase P) is shown in Figure 5. An example of WUSS notation for a local Infernal alignment of *B. subtilis* RNase P to *E. coli* RNase P, illustrating the use of local alignment annotation symbols, is in Figure 6.

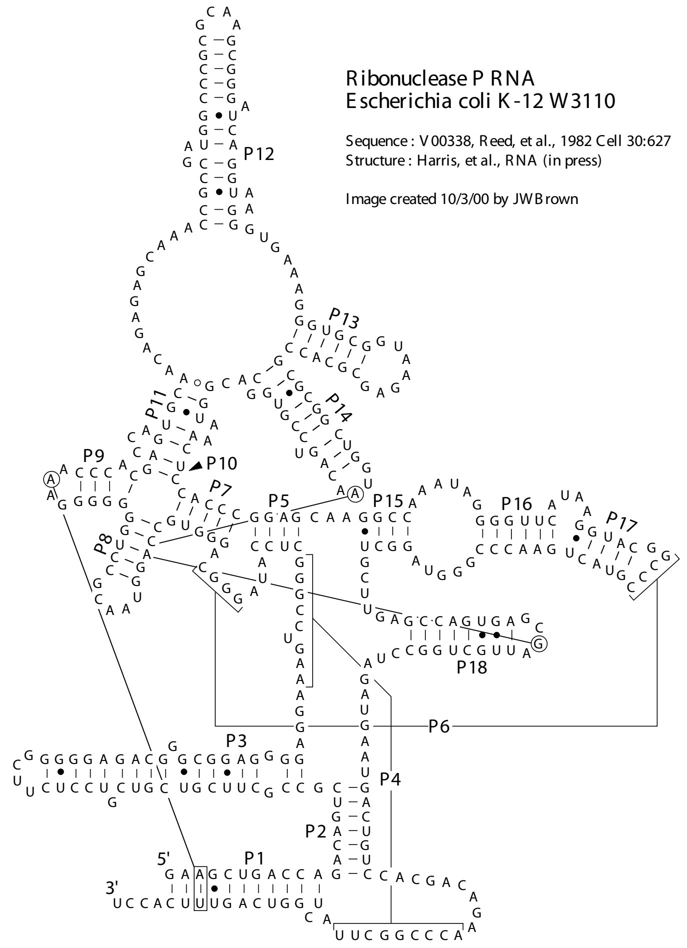


```
    {{{{{{{{{{{{{{{{{{,<<<<<<<<<<<<<-<<<<<____>>>>>>>>>->>>>>>>>
  1 GAAGCUGACCAGACAGUCGCCGCUUCGUCGUCGUCCUCUUCGGGGGAGACGGGCGGAGGG 60

    >,,,,,,,,,,,,,[[[[--------[[[[[<<<<<_____>>>>><<<<____>>>->(
 61 GAGGAAAGUCCGGGCUCCAUAGGGCAGGGUGCCAGGUAACGCCUGGGGGGGAAACCCACG 120

    (---(((((,,,,,,,,,,,,<<<<<--<<<<<<<<____>>>>>->>>>>>-->>,,,,
121 ACCAGUGCAACAGAGAGCAAACCGCCGAUGGCCCGCGCAAGCGGGAUCAGGUAAGGGUGA 180

    ,,,<<<<<<_______>>>>>><<<<<<<<<____>>>->>>>>->,,)))--))))]]]
181 AAGGGUGCGGUAAGAGCGCACCGCGCGGCUGGUAACAGUCCGUGGCACGGUAAACUCCAC 240

    ]]]]]],,,<<<<------<<<<<<----<<<<<_____>>>>>>>>>>>----->>>>,
241 CCGGAGCAAGGCCAAAUAGGGGUUCAUAAGGUACGGCCCGUACUGAACCCGGGUAGGCUG 300

    ,,,,,<<<<<<<<____>>>>>>>>,,,,,,,,,,}}}}}}}------------------
301 CUUGAGCCAGUGAGCGAUUGCUGGCCUAGAUGAAUGACUGUCCACGACAGAACCCGGCUU 360

    -}-}}}}}}}}}}::::
361 AUCGGUCAGUUUCACCU 377
```

Figure 5: **Example of WUSS notation.** Top: Secondary structure of *E. coli* RNase P, from Jim Brown's RNase P database (Brown, 1999). Bottom: WUSS notation for the same structure, annotating the *E. coli* RNase P sequence. The P4 and P6 pseudoknots are not annotated in this example.

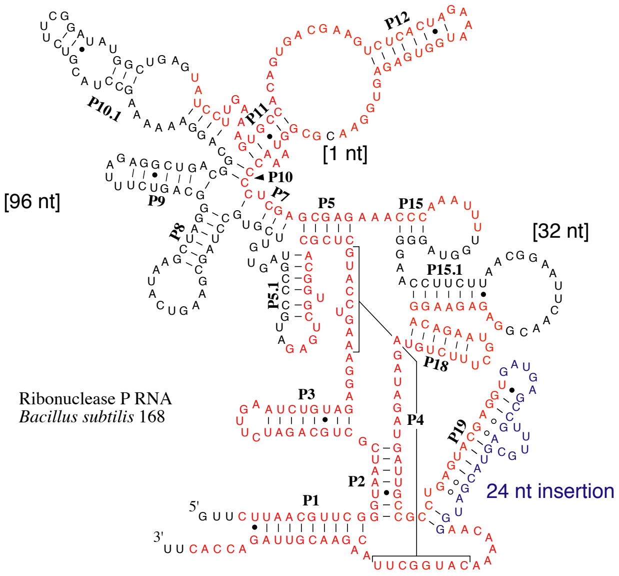


```
>> M13175.1
 rank     E-value  score  bias mdl mdl from   mdl to       seq from      seq to       acc trunc   gc
 ----   --------- ------ ----- --- -------- --------    ----------- -----------      ---- ----- ----
  (1) !   2.2e-20   58.0   0.0  cm        1      367 []           4         399 + ..    0.77    no 0.49

                    v                           v      v                                 vv         vvvvv    vvvv vv NC
                    {{{{{{{{{{{{{{{{{,<<<<<<<<<_______>>>>>>>>>,,,,,,,,,,,,,[[[[.--------[[[[[~~~~~~<<<<<____>>>>->( CS
  RNaseP_bact_a   1 cgagccggccgggcggucGCgcccccccuuaaaagggggggcGAGGAAAGUCCGGgCUcC.AcAGGgCAggguG*[15]*cggggGugAccccAgG 104
                       ::CG::CGGG:::UCGC::C:::: U      ::::G::GAGGAAAGUCC  GCUC  AC G GC   G:G               +++C +
       M13175.1   4 CUUAACGUUCGGGUAAUCGCUGCAGAUCU---UGAAUCUGUAGAGGAAAGUCCAUGCUCGcACGGUGCU--GAG*[96]*---------UAUCCUU 175
                    **************************974...4579**********************98788777776..555...8...........3444468 PP

                                                                                           v   vv        vvvv        NC
                    (---(((((,,,,,,,,,,,<<<<<<<<<<<_____>>>>>>>>>-->>,,,,,,~~~~~~,,)))--))))]]]]].]]]],,,<<<<-----   CS
  RNaseP_bact_a 105 GAaAGugCcACAGAAAaaAgACCgCccgccccuuaaggggcggGcAAGGGUGAAA*[43]*uagGcAAaCCCCaccc.GgAGCAAggccAAAUA   234
                    GAAAGU:CCACAG +A  A+ :C   :::C:: +AA::G:::     G:GUG AA        GG:AAACC C:C +  GAG AA  C+AA U
       M13175.1 176 GAAAGUGCCACAGUGACGAAGUC---UCACUAGAAAUGGUGA-----GAGUGGAA*[ 1]*GCGGUAAACCCCUCGAgCGAGAAACCCAAAUUU   256
                    99*****************8866...4455558888555444.....66999987...6..99*********9766557789988876544332   PP

                          vvvv                                                                                       NC
                    ~~~~~~>>>>,,,,,,<<<<<<.<<____>>>>>>>>,,,,,,,,,,}}}}}}}---------------........................--- CS
  RNaseP_bact_a 235 *[39]*ggccGCUuGAGccggc.cgGuAAcggccggCCuAGAugAAUgaccgcccucuuguuaaauuuu........................aAC 338
                              G     :::: : C:G AA:G: :::: UAGAU++AUGA:::CC  CU + UA  +  U                        AAC
       M13175.1 257 *[32]*----GAG---AGAAGGaCAG-AAUGCUUUCUGUAGAUAGAUGAUUGCCGCCUGAGUACGAGGUgaugagccguuugcaguacgauggAAC 370
                    ...3......222...2222221222.335555566699***************99988888888888899999********************** PP

                                            v     NC
                    -------------}-}}}}}}}}}}:::: CS
  RNaseP_bact_a 339 AGAAcCCGGCUUAcaggccggcucgucuu 367
                    A AAC  GGCUUACAG::CG::    C+
       M13175.1 371 AAAACAUGGCUUACAGAACGUUAGACCAC 399
                    ***************************** PP
```

Figure 6: **Local alignment annotation example.** Top: Secondary structure of *B. subtilis* RNase P, from Jim Brown's RNase P database (Brown, 1999). Residues in red are those that Infernal aligns to a CM of *E. coli* type RNase P's (the RNase P bacterial type A model built from the Rfam 10.1 RF00010 seed alignment using default Infernal 1.1 `cmbuild` and `cmcalibrate`). The local structural alignment is in four pieces; three regions of the structure (96, 1, and 32 nt long) are skipped over (i.e. not aligned to the type A model). One additional stem is treated as a 24 nt insertion. Bottom: the Infernal `cmsearch` output showing the RNase P type A query model, which corresponds closely to the *E. coli* structure, aligned to the *B. subtilis* sequence. The three skipped regions (96, 1, and 32 nt long) of the *B. subtilis* structure from the top of the figure are "local end" emissions which skip 15, 43, and 39 consensus positions of the type A model, respectively.

Shorthand (input) WUSS notation

While WUSS notation makes it easier to visually interpret Infernal *output* structural annotation, it would be painful to be required to *input* all structures in full WUSS notation. Therefore when Infernal reads input secondary structure annotation, it uses simpler rules:

Base pairs Any matching nested pair of `()`, `()`, `[]`, `{}` symbols indicates a base pair; the exact choice of symbol has no meaning, so long as the left and right partners match up.

Single stranded residues All other symbols `_-,:.~` indicate single stranded residues. The choice of symbol has no special meaning. Annotated pseudoknots (nested matched pairs of upper/lower case alphabetic characters) are also interpreted as single stranded residue in Infernal input.

Thus, for instance, `<<<<....>>>>` and `((((____))))` and `<(<(._._)>)>` all indicate a four base stem with a four base loop (the last example is legal but weird).

Remember that the key property of canonical (nonpseudoknotted) RNA secondary structure is that the pairs are *nested*. `((<<....))>>` is not a legal annotation string: the pair symbols don't match up properly. Infernal will reject such an annotation and report an input format error, suspecting a problem with your annotation. If you want to annotate pseudoknots, WUSS notation allows alphabetic symbols Aa, Bb, etc. see above; but remember that Infernal ignores pseudoknotted stems and treats them as single stranded residues.

Because many other RNA secondary structure analysis programs use a simple bracket notation for annotating structure, Infernal's ability to input this format makes it easier to use data generated by other RNA software packages. Conversely, converting Infernal output WUSS notation to simple bracket notation is a matter of a simple Perl or sed script, substituting the symbols appropriately.

Stockholm, the recommended multiple sequence alignment format

The Rfam and Pfam Consortiums have developed a multiple sequence alignment format called "Stockholm format" that allows rich and extensible annotation.

Crucially for Infernal, Stockholm alignments support the annotation of a consensus secondary structure, which is why `cmbuild` requires its input alignment files to be in Stockholm format. Here is a minimal Stockholm file with consensus secondary structure annotation in shorthand WUSS notation (described earlier in this section).

```
# STOCKHOLM 1.0

seq1           ACCGUC...GCAA...GG
seq2           ACCGUC...GCAA...GG
seq3           .CCUUCGUCGGAUGACGA
#=GC SS_cons   ...<<<.........>>

seq1           CGAUAC
seq2           CG..AC
seq3           ACAUCC
#=GC SS_cons   >.....
//
```

The first line in the file must be `# STOCKHOLM 1.x`, where `x` is a minor version number for the format specification (and which currently has no effect on my parsers). This line allows a parser to instantly identify the file format.

In the alignment, each line contains a name, followed by the aligned sequence. A dash, period, underscore, or tilde (but not whitespace) denotes a gap. If the alignment is too long to fit on one line, the alignment may be split into multiple blocks, with blocks separated by blank lines, as this example is. The number of sequences, their order, and their names must be the same in every block. Within a given block, each (sub)sequence (and any associated `#=GR` and `#=GC` markup, such as the `SS_cons` lines, see below) is of equal length, called the *block length*. Block lengths may differ from block to block. The block length must be at least one residue, and there is no maximum.

Other blank lines are ignored. You can add comments anywhere to the file (even within a block) on lines starting with a `#`.

The `SS_cons` line defines the consensus secondary structure in shorthand WUSS notation, as described earlier in this section.

All other annotation is added using a tag/value comment style. The tag/value format is inherently extensible, and readily made backwards-compatible; unrecognized tags will simply be ignored. Extra annotation includes individual sequence RNA or protein secondary structure, sequence weights, a reference coordinate system for the columns, and database source information including name, accession number, and coordinates (for subsequences extracted from a longer source sequence) See below for details.

syntax of Stockholm markup

There are four types of Stockholm markup annotation, for per-file, per-sequence, per-column, and per-residue annotation:

#=GF <tag> <s> Per-file annotation. <s> is a free format text line of annotation type <tag>. For example, `#=GF DATE April 1, 2000`. Can occur anywhere in the file, but usually all the #=GF markups occur in a header.

#=GS <seqname> <tag> <s> Per-sequence annotation. <s> is a free format text line of annotation type tag associated with the sequence named <seqname>. For example, `#=GS seq1 SPECIES_SOURCE Caenorhabditis elegans`. Can occur anywhere in the file, but in single-block formats (e.g. the Pfam distribution) will typically follow on the line after the sequence itself, and in multi-block formats (e.g. Infernal output), will typically occur in the header preceding the alignment but following the #=GF annotation.

#=GC <tag> <..s..> Per-column annotation. <..s..> is an aligned text line of annotation type <tag>. #=GC lines are associated with a sequence alignment block; <..s..> is aligned to the residues in the alignment block, and has the same length as the rest of the block. Typically #=GC lines are placed at the end of each block.

#=GR <seqname> <tag> <..s..> Per-residue annotation. <..s..> is an aligned text line of annotation type <tag>, associated with the sequence named <seqname>. #=GR lines are associated with one sequence in a sequence alignment block; <..s..> is aligned to the residues in that sequence, and has the same length as the rest of the block. Typically #=GR lines are placed immediately following the aligned sequence they annotate.

semantics of Stockholm markup

Any Stockholm parser will accept syntactically correct files, but is not obligated to do anything with the markup lines. It is up to the application whether it will attempt to interpret the meaning (the semantics) of the markup in a useful way. At the two extremes are the Belvu alignment viewer and the Infernal and HMMER software packages.

Belvu simply reads Stockholm markup and displays it, without trying to interpret it at all. The tag types (#=GF, etc.) are sufficient to tell Belvu how to display the markup: whether it is attached to the whole file, sequences, columns, or residues.

Infernal uses Stockholm markup to pick up a variety of information from the Rfam multiple alignment database. The Rfam consortium therefore agrees on additional syntax for certain tag types, so Infernal can parse some markups for useful information. This additional syntax is imposed by Rfam, Pfam, Infernal, HMMER, and other software from our lab, not by Stockholm format per se. You can think of Stockholm as akin to XML, and what our software reads as akin to an XML DTD, if you're into that sort of structured data format lingo.

The Stockholm markup tags that are parsed semantically by Infernal are as follows:

recognized #=GF annotations

ID <s> Identifier. **<s>** is a name for the alignment; e.g. "RNaseP". One word. Unique in file.

AC <s> Accession. **<s>** is a unique accession number for the alignment; e.g. "RF00001". Used by the Rfam database, for instance. Often a alphabetical prefix indicating the database (e.g. "RF") followed by a unique numerical accession. One word. Unique in file.

DE <s> Description. **<s>** is a free format line giving a description of the alignment; e.g. "Ribonuclease P RNA". One line. Unique in file.

AU <s> Author. **<s>** is a free format line listing the authors responsible for an alignment; e.g. "Bateman A". One line. Unique in file.

GA <f> Gathering threshold. The Infernal bit score cutoff used in gathering the members of Rfam full alignments.

NC <f> Noise cutoff. The Infernal bit score cutoff, set according to the highest scores seen for nonhomologous sequence hits when gathering members of Rfam full alignments.

TC <f> Trusted cutoff. The Infernal bit score cutoff, set according to the lowest scores seen for true homologous sequence hits that were above the GA gathering thresholds, when gathering members of Rfam full alignments.

recognized #=GS annotations

WT <f> Weight. **<f>** is a positive real number giving the relative weight for a sequence, usually used to compensate for biased representation by downweighting similar sequences. Usually the weights average 1.0 (e.g. the weights sum to the number of sequences in the alignment) but this is not required. Either every sequence must have a weight annotated, or none of them can.

AC <s> Accession. **<s>** is a database accession number for this sequence. (Compare the `#=GF AC` markup, which gives an accession for the whole alignment.) One word.

DE <s> Description. **<s>** is one line giving a description for this sequence. (Compare the `#=GF DE` markup, which gives a description for the whole alignment.)

recognized #=GC annotations

RF Reference line. Any character is accepted as a markup for a column. The intent is to allow labeling the columns with some sort of mark. `cmbuild` uses this annotation to determine which columns are consensus versus insertion if the `--hand` option is used; insertion columns are annotated by a gap symbol, and consensus columns by any non-gap symbol.

SS_cons Secondary structure consensus. When this line is generated by Infernal, it is generated in full WUSS notation. When it is read by `cmbuild`, it is interpreted more loosely, in shorthand (input) WUSS notation: pairs of symbols `<>`, `()`, `[]`, or `[]` mark consensus base pairs, and symbols `:_-,.~` mark single stranded columns (see the section on WUSS format above for details).

recognized #=GR annotations

SS Secondary structure consensus. See `#=GC SS_cons` above.

PP Posterior probability for an aligned residue. This represents the probability that this residue is assigned to the CM state corresponding to this alignment column, as opposed to some other state. The posterior probability is encoded as 11 possible characters `0-9*`: $0.0 \leq p < 0.05$ is coded as 0, $0.05 \leq p < 0.15$ is coded as 1, (... and so on ...), $0.85 \leq p < 0.95$ is coded as 9, and $0.95 \leq p \leq 1.0$ is coded as '*'. Gap characters appear in the PP line where no residue has been assigned.

Sequence files: FASTA format

FASTA is probably the simplest of formats for unaligned sequences. FASTA files are easily created in a text editor. Each sequence is preceded by a line starting with >. The first word on this line is the name of the sequence. The rest of the line is a description of the sequence (free format). The remaining lines contain the sequence itself. You can put as many letters on a sequence line as you want. For example:

```
>seq1 This is the description of my first sequence.
AGTACGTAGTAGCTGCTGCTACGTGCGCTAGCTAGTACGTCA CGACGTAGATGCTAGCTGACTCGATGC
>seq2 This is a description of my second sequence.
CGATCGATCGTACGTCGACTGATCGTAGCTACGTCGTACGTAG CATCGTCAGTTACTGCATGCTCG
CATCAGGCATGCTGCTGACTGATCGTACG
```

For better or worse, FASTA is not a documented standard. Minor (and major) variants are in widespread use in the bioinformatics community, all of which are called "FASTA format". My software attempts to cater to all of them, and is tolerant of common deviations in FASTA format. Certainly anything that is accepted by the database formatting programs in NCBI BLAST or WU-BLAST (e.g. setdb, pressdb, xdformat) will also be accepted by my software. Blank lines in a FASTA file are ignored, and so are spaces or other gap symbols (dashes, underscores, periods) in a sequence. Other non-amino or non-nucleic acid symbols in the sequence are also silently ignored, mostly because some people seem to think that "*" or "." should be added to protein sequences to (redundantly) indicate the end of the sequence. The parser will also accept unlimited line lengths, which allows it to accomodate the enormous description lines in the NCBI NR databases.

(On the other hand, any FASTA files *generated* by my software adhere closely to community standards, and should be usable by other software packages (BLAST, FASTA, etc.) that are more picky about parsing their input files. That means you can run a sloppy FASTA file thru the `sreformat` utility program to clean it up.)

Partly because of this tolerance, the software may have a difficult time dealing with files that are *not* in FASTA format, especially if you're relying on file format autodetection (the "Babelfish"). Some (now mercifully uncommon) file formats are so similar to FASTA format that they be erroneously called FASTA by the Babelfish and then quietly and lethally misparsed. An example is the old NBRF file format. If you're afraid of this, you can use the `--informat fasta` option to bypass the Babelfish and improve robustness. However, it is still possible to construct files perversely similar to FASTA that will still confuse the parser. (The gist of these caveats applies to all formats, not just FASTA.)

Null model file format

The Infernal source distribution includes an example null model file, `src/rna.null`. This null model is identical to the hardcoded default prior used by Infernal, all four RNA nucleotides are equiprobable in the null, background model.

A null model file must contain exactly four non-comment lines. A comment line begins with a "# ", that is a # followed by a single space. Each of the four non-comment lines must contain a single floating point number, the four of which sum to 1.0. The first non-comment line is interpreted as the background probability of an "A" residue, the second, third, and fourth non-comment lines are interpreted as the background probabilities of a "C", "G" and "U" respectively.

Clan input file format for cmscan

The `cmscan` program has a `--clanin <f>` option that allows the user to supply an input file `<f>` with information on clan membership for models in the CM file. This option must be used in combination with the `--tblout` and `--fmt 2` options. An example clan input file is included with the Infernal source distribution, `tutorial/Rfam.12.1.clanin`. This file specifies the clan membership for the 2474 models in the Rfam 12.1 release, of which 311 models belong to 104 clans. This file should be used in combination with the Rfam.cm file for Rfam 12.1, available for download as a gzipped file here: `ftp://ftp.ebi.ac.uk/pub/databases/Rfam/12.1/Rfam.cm.gz`. Note that many of the Rfam models are not members of a clan; the clan input file does not need to specify clan membership for all models in the CM file.

A clan input file contains one line per clan. Each line must contain at least two space-delimited tokens. The first token is the name of the clan (this name cannot contain spaces). Each token after the first is the name of a model that is a member of the clan named in the first token. These tokens must be valid names of models in the file CM file you are using with `cmscan`. These tokens cannot be the accessions of models. Valid model names cannot contain spaces (enforced by `cmbuild` during model construction). To determine the names of models in a CM file, use `cmstat`.

For example, in the file `tutorial/Rfam.12.1.clanin` the first token of the first line is "CL00001" and tokens two through five are "tRNA", "cyano_tmRNA", "tRNA-Sec", "mt-tmRNA", indicating that these four models are members of the CL00001 clan. `cmscan` will output the clan name of models in clans in its tabular output file specified with `--tblout` when the `--fmt 2` option is also used. Furthermore, you can specify that only overlapping hits between models of the same clan are annotated (as opposed to all overlapping hits) in the tabular output file by additionally using the `--oclan` option. Finally, you can specify that lower scoring overlaps within clans are not output by additionally using the `--oskip` and the `--oclan` options.

Dirichlet prior files

A prior file is parsed into a number of whitespace-delimited, non-comment fields. These fields are then interpreted in order. The order and number of the fields is important. This is not a robust, tag-value save file format.

All whitespace is ignored, including newlines. The number of fields per line is unimportant.

Comments begin with a `#` character. The remainder of any line following a `#` is ignored.

The Infernal source distribution includes an example prior file, `default.pri`. This prior is identical to the hard-coded default prior used by Infernal. The following text may only make sense if you're looking at that example while you read.

The order of the fields in the prior file is as follows:

Strategy. The first field is the keyword **`Dirichlet`**. Currently Dirichlet priors (mixture or not) are the only prior strategy used by Infernal.

Transition prior section. The next field is the number **74**, the number of different types of transition distributions. (See Figure 7 for an explanation of where the number 74 comes from.) Then, for each of these 74 distributions:

<from-uniqstate> <to-node>: Two fields give the transition type: from a unique state identifier, to a node identifier. Example: **MATP_MP MATP**.

<n>: One field gives the number of transition probabilities for this transition type; that is, the number of Dirichlet parameter vector $\alpha_1^q..\alpha_n^q$ for each mixture component q.

<nq>: One field gives the number of mixture Dirichlet components for this transition type's prior. Then, for each of these **nq** Dirichlet components:

p(q): One field gives the mixture coefficient $p(q)$, the prior probability of this component q. For a single-component "mixture", this is always 1.0.

$\alpha_\mathbf{1}^\mathbf{q}..\alpha_\mathbf{n}^\mathbf{q}$: The next n fields give the Dirichlet parameter vector for this mixture component q.

Base pair emission prior section. This next section is the prior for MATP_MP emissions. One field gives **<K>**, the "alphabet size" – the number of base pair emission probabilities – which is always 16 (4x4), for RNA. The next field gives **<nq>**, the number of mixture components. Then, for each of these **nq** Dirichlet components:

p(q): One field gives the mixture coefficient $p(q)$, the prior probability of this component q. For a single-component "mixture", this is always 1.0.

$\alpha_\mathbf{AA}^\mathbf{q}..\alpha_\mathbf{UU}^\mathbf{q}$: The next 16 fields give the Dirichlet parameter vector for this mixture component, in alphabetical order (AA, AC, AG, AU, CA ... GU, UA, UC, UG, UU).

Consensus singlet base emission prior section. This next section is the prior for MATL_ML and MATR_MR emissions. One field gives **<K>**, the "alphabet size" – the number of singlet emission probabilities – which is always 4, for RNA. The next field gives **<nq>**, the number of mixture components. Then, for each of these **nq** Dirichlet components:

p(q): One field gives the mixture coefficient $p(q)$, the prior probability of this component q. For a single-component "mixture", this is always 1.0.

$\alpha_\mathbf{A}^\mathbf{q}..\alpha_\mathbf{U}^\mathbf{q}$: The next 4 fields give the Dirichlet parameter vector for this mixture component, in alphabetical order (A, C, G, U).

Nonconsensus singlet base emission prior section. This next section is the prior for insertions (MATP_IL, MATP_IR, MATL_IL, MATR_IR, ROOT_IL, ROOT_IR, BEGR_IL) as well as nonconsensus singlets (MATP_ML, MATP_MR). One field gives **<K>**, the "alphabet size" – the number of singlet emission probabilities – which is always 4, for RNA. The next field gives **<nq>**, the number of mixture components. Then, for each of these **nq** Dirichlet components:

p(q): One field gives the mixture coefficient $p(q)$, the prior probability of this component q. For a single-component "mixture", this is always 1.0.

$\alpha_\mathbf{A}^\mathbf{q}..\alpha_\mathbf{U}^\mathbf{q}$: The next 4 fields give the Dirichlet parameter vector for this mixture component, in alphabetical order (A, C, G, U).

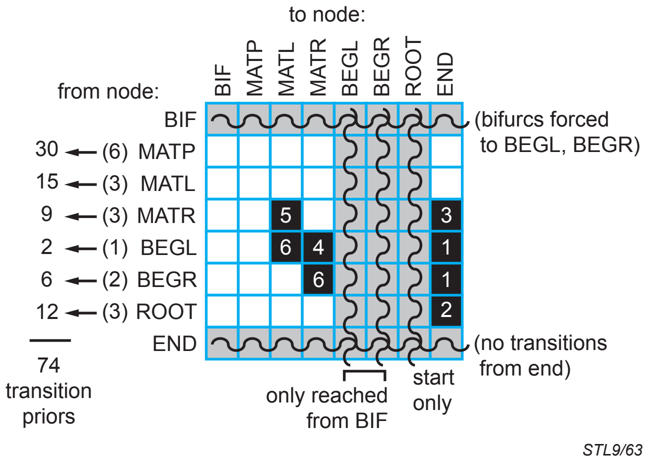

Figure 7: **Where does the magic number of 74 transition distribution types come from?** The transition distributions are indexed in a 2D array, from a unique statetype (20 possible) to a downstream node (8 possible), so the total conceivable number of different distributions is $20 \times 8 = 160$. The grid represents these possibilities by showing the 8×8 array of all node types to all node types; each starting node contains 1 or more unique states (number in parentheses to the left). Two rows are impossible (gray): bifurcations automatically transit to determined BEGL, BEGR states with probability 1, and end nodes have no transitions. Three columns are impossible (gray): BEGL and BEGR can only be reached by probability 1 transitions from a bifurcation, and the ROOT node is special and can only start a model. Eight individual cells of the grid are unused (black) because of the way `cmbuild` (almost) unambiguously constructs a guide tree from a consensus structure. These cases are numbered as follows. (1) BEGL and BEGR never transit to END; this would imply an empty substructure. A bifurcation is only used if both sides of the split contain at least one consensus pair (MATP). (2) ROOT never transits to END; this would imply an alignment with zero consensus columns. Infernal models assume ≥ 1 consensus columns. (3) MATR never transits to END. Infernal always uses MATL for unpaired columns whenever possible. MATR is only used for internal loops, multifurcation loops, and 3' bulges, so MATR must always be followed by a BIF, MATP, or another MATR. (4) BEGL never transits to MATR. The single stranded region between two bifurcated stems is unambiguously assigned to MATL nodes on the right side of the split, not to MATR nodes on the left. (5) MATR never transits to MATL. The only place where this could arise (given that we already specified that MATL is used whenever possible) is in an interior loop; there, by unambiguous convention, MATL nodes precede MATR nodes. (6) BEGL nodes never transit to MATL, and BEGR nodes never transit to MATR. By convention, at any bifurcated subsequence i, j, i and j are paired but not to each other. That is, the smallest possible subsequence is bifurcated, so that any single stranded stretches to the left and right are assigned to MATL and MATR nodes above the bifurcation, instead of MATL nodes below the BEGL and MATR nodes below the BEGR. Thus, the total number 74 comes from multiplying, for each row, the number of unique states in each starting node by the number of possible downstream nodes (white), and summing these up, as shown to the left of the grid.

10 Acknowledgements

Infernal relies heavily on HMMER and Easel, originally created by Sean Eddy. Several others have helped develop these two packages as well, including Steve Johnson, Alex Coventry, Dawn Brooks, Sergi Castellano, Michael Farrar, Travis Wheeler, and Elena Rivas. In particular, the improved speed of Infernal 1.1 is enabled by research and development for the HMMER3 project, mainly from Sean, Travis and Michael. Further, many of the changes made for Infernal 1.1 mirror features in HMMER3, and were implemented frequently by stealing and slightly modifying code. Even this guide is based heavily on HMMER3's guide, and some analogous sections are identical or near identical. Additionally, the RSEARCH program (Klein and Eddy, 2003) from Robbie Klein has also had an important impact on Infernal, which still includes some of its code.

Sean created and was the lone developer of Infernal up through the version 0.55 release in 2003. Two graduate students, Diana Kolbe and Eric Nawrocki, focused on improvements to Infernal for their graduate work, beginning in 2004. Their efforts combined with Sean's led to versions 0.56 through 1.0.2. Diana has moved onto a postdoc, but included a snapshot of the codebase in between the 1.0.2 and 1.1 releases as supplementary material with her thesis. Eric continues to develop Infernal and is responsible for most of the changes in the 1.1 release.

The concept of HMM banded SCFG alignment implemented in Infernal derives from Michael Brown's RNACAD software, developed while he was working with David Haussler at UC Santa Cruz (Brown, 2000). HMM filtering for CMs was pioneered by Zasha Weinberg and Larry Ruzzo at the University of Washington (Weinberg and Ruzzo, 2004a,b, 2006). The CP9 HMMs in Infernal are a reimplementation of a profile HMM architecture introduced by Weinberg.

Infernal testing requires *a lot* of compute power, and we are extremely fortunate to have access to a highly reliable and state-of-the-art computing cluster, thanks to Jesse Becker, Ron Patterson and others at NCBI.

Infernal is primarily developed on GNU/Linux and Apple Macintosh machines, but is tested on a variety of hardware. Over the years, Compaq, IBM, Intel, Sun Microsystems, Silicon Graphics, Hewlett-Packard, Paracel, and nVidia have provided generous hardware support that makes this possible. We owe a large debt to the free software community for the development tools we use: an incomplete list includes GNU gcc, gdb, emacs, and autoconf; the amazing valgrind; the indispensable Subversion; the ineffable perl; LaTeX and TeX; PolyglotMan; and the UNIX and Linux operating systems.

References

Bernhart, S. H., Hofacker, I. L., Will, S., Gruber, A. R., and Stadler, P. F. (2008). RNAalifold: improved consensus structure prediction for RNA alignments. *BMC Bioinformatics*, 9:474.

Brown, J. W. (1999). The ribonuclease P database. *Nucl. Acids Res.*, 27:314.

Brown, M. P. (2000). Small subunit ribosomal RNA modeling using stochastic context-free grammars. *Proc. Int. Conf. Intell. Syst. Mol. Biol.*, 8:57–66.

Cole, J. R., Wang, Q., Cardenas, E., Fish, J., Chai, B., Farris, R. J., Kulam-Syed-Mohideen, A. S., McGarrell, D. M., Marsh, T., Garrity, G. M., and Tiedje, J. M. (2009). The Ribosomal Database Project: Improved alignments and new tools for rRNA analysis. *Nucl. Acids Res.*, 37:D141–D145.

Durbin, R., Eddy, S. R., Krogh, A., and Mitchison, G. J. (1998). *Biological Sequence Analysis: Probabilistic Models of Proteins and Nucleic Acids*. Cambridge University Press, Cambridge UK.

Eddy, S. R. (1996). Hidden Markov models. *Curr. Opin. Struct. Biol.*, 6:361–365.

Eddy, S. R. (1998). Profile hidden Markov models. *Bioinformatics*, 14:755–763.

Eddy, S. R. (2002). A memory-efficient dynamic programming algorithm for optimal alignment of a sequence to an RNA secondary structure. *BMC Bioinformatics*, 3:18.

Eddy, S. R. (2006). Computational analysis of RNAs. *Cold Spring Harbor Symp. Quant. Biol.*, 71:117–128.

Eddy, S. R. (2008). A probabilistic model of local sequence alignment that simplifies statistical significance estimation. *PLOS Comput. Biol.*, 4:e1000069.

Eddy, S. R. (2009). The HMMER3 user's guide. [http://hmmer.org/].

Eddy, S. R. (2011). Accelerated profile HMM searches. *PLOS Comp. Biol.*, 7:e1002195.

Eddy, S. R. and Durbin, R. (1994). RNA sequence analysis using covariance models. *Nucl. Acids Res.*, 22:2079–2088.

Gardner, P. P., Daub, J., Tate, J., Moore, B. L., Osuch, I. H., Griffiths-Jones, S., Finn, R. D., Nawrocki, E. P., Kolbe, D. L., Eddy, S. R., and Bateman, A. (2011). Rfam: Wikipedia, clans and the "decimal" release. *Nucl. Acids Res.*, 39:D141–D145.

Gerstein, M., Sonnhammer, E. L. L., and Chothia, C. (1994). Volume changes in protein evolution. *J. Mol. Biol.*, 235:1067–1078.

Giegerich, R. (2000). Explaining and controlling ambiguity in dynamic programming. In Giancarlo, R. and Sankoff, D., editors, *Proceedings of the 11th Annual Symposium on Combinatorial Pattern Matching*, number 1848, pages 46–59, Montréal, Canada. Springer-Verlag, Berlin.

Henikoff, S. and Henikoff, J. G. (1994). Position-based sequence weights. *J. Mol. Biol.*, 243:574–578.

Hofacker, I. L., Fekete, M., and Stadler, P. F. (2002). Secondary structure prediction for aligned RNA sequences. *J. Mol. Biol.*, 319:1059–1066.

Holmes, I. (1998). *Studies in Probabilistic Sequence Alignment and Evolution*. PhD thesis, University of Cambridge.

Karplus, K., Barrett, C., and Hughey, R. (1998). Hidden Markov models for detecting remote protein homologies. *Bioinformatics*, 14:846–856.

Klein, R. J. (2003). *Finding Noncoding RNA Genes in Genomic Sequences*. PhD thesis, Washington University School of Medicine.

Klein, R. J. and Eddy, S. R. (2003). RSEARCH: finding homologs of single structured RNA sequences. *BMC Bioinformatics*, 4:44.

Kolbe, D. L. (2010). *Novel Algorithms for Structural Alignment of Non-Coding RNAs*. PhD thesis, Washington University School of Medicine.

Kolbe, D. L. and Eddy, S. R. (2009). Local RNA structure alignment with incomplete sequence. *Bioinformatics*, 25:1236–1243.

Kolbe, D. L. and Eddy, S. R. (2011). Fast filtering for RNA homology search. *Bioinformatics*, 27:3102–3109.

Krogh, A. (1998). An introduction to hidden Markov models for biological sequences. In Salzberg, S., Searls, D., and Kasif, S., editors, *Computational Methods in Molecular Biology*, pages 45–63. Elsevier.

Krogh, A., Brown, M., Mian, I. S., Sjölander, K., and Haussler, D. (1994). Hidden Markov models in computational biology: Applications to protein modeling. *J. Mol. Biol.*, 235:1501–1531.

Lari, K. and Young, S. J. (1990). The estimation of stochastic context-free grammars using the inside-outside algorithm. *Computer Speech and Language*, 4:35–56.

Nawrocki, E. P. (2009). *Structural RNA Homology Search and Alignment Using Covariance Models*. PhD thesis, Washington University School of Medicine.

Nawrocki, E. P., Burge, S. W., Bateman, A., Daub, J., Eberhardt, R. Y., Eddy, S. R., Floden, E. W., Gardner, P. P., Jones, T. A., Tate, J., and Finn, R. D. (2015). Rfam 12.0: updates to the RNA families database. *Nucl. Acids Res.*, 43:D130–D137.

Nawrocki, E. P. and Eddy, S. R. (2007). Query-dependent banding (QDB) for faster RNA similarity searches. *PLOS Comput. Biol.*, 3:e56.

Nawrocki, E. P., Kolbe, D. L., and Eddy, S. R. (2009). Infernal 1.0: Inference of RNA alignments. *Bioinformatics*, 25:1335–1337.

Rabiner, L. R. (1989). A tutorial on hidden Markov models and selected applications in speech recognition. *Proc. IEEE*, 77:257–286.

Randau, L., Münch, R., Hohn, M. J., Jahn, D., and Söll, D. (2005). Nanoarchaeum equitans creates functional tRNAs from separate genes for their 5' and 3'-halves. *Nature*, 433:537–541.

Sakakibara, Y., Brown, M., Hughey, R., Mian, I. S., Sjölander, K., Underwood, R. C., and Haussler, D. (1994). Stochastic context-free grammars for tRNA modeling. *Nucl. Acids Res.*, 22:5112–5120.

Sjölander, K., Karplus, K., Brown, M., Hughey, R., Krogh, A., Mian, I. S., and Haussler, D. (1996). Dirichlet mixtures: A method for improving detection of weak but significant protein sequence homology. *Comput. Applic. Biosci.*, 12:327–345.

Torarinsson, E. and Lindgreen, S. (2008). WAR: webserver for aligning structural RNAs. *Nucleic Acids Res.*, 36:W79–W84.

Tringe, S. G., von Mering, C., Kobayashi, A., Salamov, A. A., Chen, K., Chang, H. W., Podar, M., Short, J. M., Mathur, E. J., Detter, J. C., Bork, P., Hugenholtz, P., and Rubin, E. M. (2005). Comparative metagenomics of microbial communities. *Science*, 308:554–557.

Weinberg, Z. and Ruzzo, W. L. (2004a). Exploiting conserved structure for faster annotation of non-coding RNAs without loss of accuracy. *Bioinformatics*, 20 Suppl. 1:I334–I341.

Weinberg, Z. and Ruzzo, W. L. (2004b). Faster genome annotation of non-coding RNA families without loss of accuracy. *RECOMB '04*, pages 243–251.

Weinberg, Z. and Ruzzo, W. L. (2006). Sequence-based heuristics for faster annotation of non-coding RNA families. *Bioinformatics*, 22:35–39.
